# Genomic history of coastal societies from eastern South America

**DOI:** 10.1038/s41559-023-02114-9

**Published:** 2023-07-31

**Authors:** Tiago Ferraz, Ximena Suarez Villagran, Kathrin Nägele, Rita Radzevičiūtė, Renan Barbosa Lemes, Domingo C. Salazar-García, Verônica Wesolowski, Marcony Lopes Alves, Murilo Bastos, Anne Rapp Py-Daniel, Helena Pinto Lima, Jéssica Mendes Cardoso, Renata Estevam, Andersen Liryo, Geovan M. Guimarães, Levy Figuti, Sabine Eggers, Cláudia R. Plens, Dionne Miranda Azevedo Erler, Henrique Antônio Valadares Costa, Igor da Silva Erler, Edward Koole, Gilmar Henriques, Ana Solari, Gabriela Martin, Sérgio Francisco Serafim Monteiro da Silva, Renato Kipnis, Letícia Morgana Müller, Mariane Ferreira, Janine Carvalho Resende, Eliane Chim, Carlos Augusto da Silva, Ana Claudia Borella, Tiago Tomé, Lisiane Müller Plumm Gomes, Diego Barros Fonseca, Cassia Santos da Rosa, João Darcy de Moura Saldanha, Lúcio Costa Leite, Claudia M. S. Cunha, Sibeli Aparecida Viana, Fernando Ozorio Almeida, Daniela Klokler, Henry Luydy Abraham Fernandes, Sahra Talamo, Paulo DeBlasis, Sheila Mendonça de Souza, Claide de Paula Moraes, Rodrigo Elias Oliveira, Tábita Hünemeier, André Strauss, Cosimo Posth

**Affiliations:** 1 Institute of Biosciences, Genetics Department, University of São Paulo, São Paulo, Brazil; 2 Museum of Archaeology and Ethnology, University of São Paulo, São Paulo, Brazil; 3 Department of Archaeogenetics, Max Planck Institute for Evolutionary Anthropology, Leipzig, Germany; 4 Departament de Prehistòria, Arqueologia i Història Antiga, Universitat de València, València, Spain; 5 Department of Geological Sciences, University of Cape Town, Cape Town, South Africa; 6 Departamento de Antropologia, Museu Nacional, Universidade Federal do Rio de Janeiro, Rio de Janeiro, Brazil; 7 Federal University of Western Pará (UFOPA), Pará, Brazil; 8 Museu Paraense Emílio Goeldi, Pará, Brazil; 9 Géosciences Environnement Toulouse, Observatoire Midi Pyrénées, UMR 5563, CNRS, Toulouse, France; 10 National Museum, Federal University of Rio de Janeiro, Rio de Janeiro, Brazil; 11 Grupo de Pesquisa em Educação Patrimonial e Arqueologia (Grupep), Universidade do Sul de Santa Catarina, Santa Catarina, Brazil; 12 Natural History Museum of Vienna, Vienna, Austria; 13 Laboratory of Archaeological Studies, Department of History, Federal University of São Paulo, São Paulo, Brazil; 14 Federal University of Espirito Santo, Vitória, Brazil; 15 Independent researcher, Belo Horizonte, Brazil; 16 Fundação Museu do Homem Americano, Piauí, Brazil; 17 Departamento de Arqueologia, Universidade Federal de Pernambuco, Recife, Brazil; 18 Scientia Consultoria Científica, São Paulo, Brazil; 19 Department of Archaeology, Max Planck Institute for the Science of Human History, Jena, Germany; 20 Instituto Goiano de Pré-história e Arqueologia, Pontifícia Universidade Católica de Goiás, Goiânia, Brazil; 21 Universidade Federal do Amazonas, Manaus, Brazil; 22 Universidade Federal de Minas Gerais, Belo Horizonte, Brazil; 23 Secretaria de Estado de Educação do Pará, Belém, Brazil; 24 Museu do Estado Pará (Secult), Pará, Brazil; 25 Universidade de Évora, Évora, Portugal; 26 Instituto de Pesquisas Científicas e Tecnológicas do Estado do Amapá (IEPA), Macapá, Brazil; 27 Federal University of Piauí, Piauí, Brazil; 28 Centro de Investigação em Antropologia e Saúde, Universidade de Coimbra, Coimbra, Portugal; 29 Programa de Pós-Graduação em Arqueologia, Universidade Federal de Sergipe, Sergipe, Brazil; 30 Departamento de Arqueologia, Universidade do Estado do Rio de Janeiro, Rio de Janeiro, Brazil; 31 Departamento de Antropologia e Arqueologia, Universidade Federal de Minas Gerais, Belo Horizonte, Brazil; 32 Programa de Pós-Graduação em Arqueologia e Patrimônio Cultural, Universidade Federal do Recôncavo da Bahia, Bahia, Brazil; 33 Department of Chemistry G. Ciamician, Alma Mater Studiorum, University of Bologna, Bologna, Italy; 34 Department of Human Evolution, Max Planck Institute for Evolutionary Anthropology, Leipzig, Germany; 35 Escola Nacional de Saúde Pública Sergio Arouca, Fundação Oswaldo Cruz, Rio de Janeiro, Brazil; 36 Institut de Biologia Evolutiva, CSIC/Universitat Pompeu Fabra, Barcelona, Spain; 37 Archaeo- and Palaeogenetics, Institute for Archaeological Sciences, Department of Geosciences, University of Tübingen, Tübingen, Germany; 38 Senckenberg Centre for Human Evolution and Palaeoenvironment, University of Tübingen, Tübingen, Germany

**Keywords:** Archaeology, Population genetics, Biological anthropology

## Abstract

Sambaqui (shellmound) societies are among the most intriguing archaeological phenomena in pre-colonial South America, extending from approximately 8,000 to 1,000 years before present (yr bp) across 3,000 km on the Atlantic coast. However, little is known about their connection to early Holocene hunter-gatherers, how this may have contributed to different historical pathways and the processes through which late Holocene ceramists came to rule the coast shortly before European contact. To contribute to our understanding of the population history of indigenous societies on the eastern coast of South America, we produced genome-wide data from 34 ancient individuals as early as 10,000 yr bp from four different regions in Brazil. Early Holocene hunter-gatherers were found to lack shared genetic drift among themselves and with later populations from eastern South America, suggesting that they derived from a common radiation and did not contribute substantially to later coastal groups. Our analyses show genetic heterogeneity among contemporaneous Sambaqui groups from the southeastern and southern Brazilian coast, contrary to the similarity expressed in the archaeological record. The complex history of intercultural contact between inland horticulturists and coastal populations becomes genetically evident during the final horizon of Sambaqui societies, from around 2,200 yr bp, corroborating evidence of cultural change.

## Abstract (web summary)

The authors report genome-wide data from 34 ancient individuals spanning the Holocene, from what is now Brazil, illuminating connections and disjoints between Sambaqui shellmound societies and preceding and later peoples.

## Main

The settlement of the Atlantic coast by maritime societies is a central topic in South American archaeology. Across ~3,000 km of the coast of Brazil, semi-sedentary populations, with seemingly large demography, produced thousands of shellmounds and shell middens, locally known as sambaquis (heaps of shell, in the Tupi language), for over 7,000 years. Subsistence was based on a mixed economy, combining aquatic resources and plants, complemented by hunting of terrestrial mammals and horticulture^1–8^. Sambaquis are the product of planned and long-term deposition of shells, fish remains, plants, artefacts, combustion debris and local sediments, and they were used as territorial markers, dwellings, cemeteries and/or ceremonial sites. On the southern Brazilian coast, funerary shellmounds can reach monumental heights (of up to 30 metres) and often contain hundreds of human burials, suggesting a high demographic density unparalleled in the South American lowlands^3,6,9–11^. In a singular enclave south of São Paulo State, further inland from the coast (Vale do Ribeira de Iguape), sambaqui sites are within the Atlantic Forest^12–15^. Here there is evidence of early Holocene settlement in the riverine sambaqui of Capelinha, as revealed by a male individual directly dated to ~10,400 years before present (yr bp) (we identify all analysed individuals by rounding the mean calibrated age in years bp)^10^. This individual was named ‘Luzio’, as a reference to ‘Luzia’, a final Pleistocene female skeleton found in the Lagoa Santa region in east-central Brazil^10,16,17^. Both individuals are at the centre of long-lasting debates for exhibiting the so-called paleoamerican cranial morphology that differs from that of present-day indigenous peoples^10,18^. The earliest evidence of human settlement on the Atlantic coast starts between ~8,700 and 7,000 yr bp with an intensification of sambaqui construction between 5,500 yr bp and 2,200 yr bp^2,4,6,19^. The relationship between riverine and coastal sambaquis is still a matter of debate, although bioarchaeological studies point towards a biological link^20–23^, and some researchers suggest a late Pleistocene/early Holocene cultural connection that faded through time^24–27^.

The disappearance of Sambaqui societies started 2,000 years ago, when funerary fishmounds replaced shellmounds in the territory where they previously thrived^4,28–31^. This abrupt change in the archaeological record is concomitant with environmental and ecological changes related to coastal regression and climatic events^32–36^ that had an irreversible impact on the availability of key resources. Between 1,200 and 900 years ago, thin-walled non-decorated pottery (Taquara-Itararé tradition) appeared for the first time on the southern Brazilian coast^2,6,9,11,29,37–39^. The makers of Taquara-Itararé ceramics were horticulturists that arrived in the southern Brazilian highlands about 3,000 years ago, lived in pit houses and cremated their dead in funerary mounds. They are considered to be the ancestors of present-day Jê-speaking indigenous peoples of southern Brazil (Kaingang, Xonkleng, Laklãnõ and the extinct Kimdá and Ingáin), a language family of the Macro-Jê stock^38,40–43^. The dispersal of Taquara-Itarare ceramics on the southern coast was first interpreted as resulting from the demographic expansion of inland horticulturists. However, evidence points to a complex scenario of social interaction between inland and coastal populations, with changes in funerary practices and post-marital residence patterns after the introduction of ceramics, biological continuity and maintenance of mobility patterns (with local variations), persistence in the exploitation of aquatic resources, and development of sophisticated fishing technologies^2,4,11,21,23,39,44–49^. Ceramics appear in the southeast coast about 2,000 years ago but are associated with the Una tradition, also probably produced by speakers of the Macro-Jê language stock^50,51^.

Shortly after the appearance of southern proto-Jê ceramics, another major transformation occurred on the Atlantic coast. This is documented by the arrival of speakers of the Tupi-Guarani language family (of the Tupi stock), a forest-farming culture who migrated from southern Amazonia more than 2,500 years ago in one of the largest expansion events in the indigenous history of South America. Although still a matter of debate, the Tupi-Guarani would have dispersed southwards from southwestern Amazonia (homeland of the Tupi stock) across the core of South America, reaching the La Plata basin, and almost simultaneously from southeastern Amazonia across the Atlantic coast of Brazil^38,42,52–57^. While on the southern coast of Brazil a late Tupi-Guarani chronology is well defined^38,52^, on the southeast coast a much earlier arrival (~3,000 years ago) has been proposed on the basis of the archaeological record of the Araruama region (Rio de Janeiro State)^58–60^. European colonists encountered thousands of Tupi-Guarani peoples both on the Atlantic coast and along major rivers and their tributaries in southern Brazil and northeastern Argentina (Paraná, Paraguay and Uruguay river basins). The Tupi-Guarani produced painted ceramics (red and black on white painting), applied a diversity of plastic decorations and made pots with complex and composite contours that are archaeologically defined as Tupiguarani, Tupinambá and Guarani, depending on the geographical location^42,53,61^.

Ancient DNA data from Brazil are still very sparse, with only 19 published individuals with analysable genomic coverage^62,63^. Early Holocene individuals from Lapa do Santo in the Lagoa Santa region, dated between ~9,800 and 9,200 yr bp, carried a distinct affinity to the oldest North American genome, which is associated with the Clovis cultural complex (Anzick-1, ~12,800 yr bp)^63,64^. A genetic signal of 3–5% Australasian ancestry—known as the Population Y signal—was found in present-day indigenous individuals from southwestern Amazonia, Central Brazil and the northwestern South American coast^65,66^ and in one early Holocene individual from Lapa do Sumidouro (Sumidouro 5, dated to c. 10,400 yr bp)^62^. However, this signal was not detected in the early Holocene burials from Lapa do Santo, located only four kilometres from Lapa do Sumidouro^63^. The complete absence of ancient DNA data for Amazonia and Northeast Brazil and the low-coverage data from the south/southeast Brazilian coast have prevented an assessment of whether the Population Y signal survived in those regions through time.

Regarding Sambaqui societies, three previously published middle Holocene individuals from Laranjal and Moraes (both riverine shellmounds from the southeast coast of Brazil) and five individuals from the late Holocene site of Jabuticabeira II (one of the largest coastal shellmounds in southern Brazil) showed some level of genetic continuity with present-day indigenous populations^63^. The analysed Jabuticabeira II individuals carried a significant affinity to present-day Kaingang (Jê speaking) from the southern Brazilian highlands. Although based on low-coverage genome-wide data, this supports a shared ancestry between the Sambaqui societies and the speakers of proto-Jê^63^.

The long-term permanence, cultural similarity and rapid disappearance of Sambaqui societies, plus their archaeological and seemingly genetic disconnection from early Holocene hunter-gatherers, raise numerous questions about their origins and demographic history. First, were Sambaqui individuals genetically different from hunter-gatherers from the hinterland (for example, east-central and northeastern Brazil)? Second, were the riverine Sambaqui groups genetically related to the ones on coastal sites? Third, was there genetic homogeneity across Sambaqui groups from the south and southeast coast of Brazil? Fourth, was the demise of sambaqui construction after 2,000 yr bp and the appearance of ceramics associated with an intensification of contacts with inland populations? Finally, are there genetic connections between Sambaqui groups and other archaeological and present-day indigenous populations from Amazonia and central and northeastern Brazil?

## Results

### Dataset and ancient DNA authenticity

**Fig. 1:**
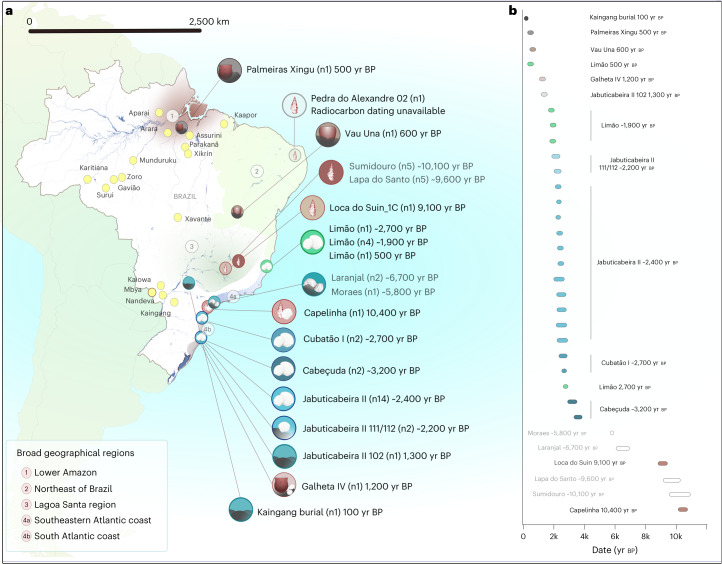
Geographic and temporal distribution of analysed genome-wide data from Brazil. **a**, The archaeological sites analysed in this study, with the number of analysed individuals reported in brackets. Sites with newly reported genome-wide data are shown in black font, and those with previously published genome-wide data are shown in grey (this color scheme is maintained in all main text figures). The symbols used for each site refer to the associated archaeological cultures (see the legend in Extended Data Fig. 8). The shaded areas represent the broad geographic regions analysed in this work: (1) lower Amazon, (2) northeastern Brazil, (3) Lagoa Santa, (4a) southeastern Atlantic coast and (4b) southern Atlantic coast. The Kaingang burial is geographically closer to the southeastern Atlantic coast but was included in the southern Atlantic group due to its specific genetic affinity. The locations of present-day indigenous groups are represented with yellow dots. **b**, The calibrated ages (coloured bars) of single directly dated individuals with new genomic data and, in black font, the mean calibrated ages for the respective groups/individuals. For the previously published ancient genome-wide data^62,63^, the mean calibrated ages for the respective groups/individuals are reported in grey, whereas the white bars represent the temporal range of all directly dated individuals included in each group. Figure related to Supplementary Data 1.

**Extended Data Fig. 1:**
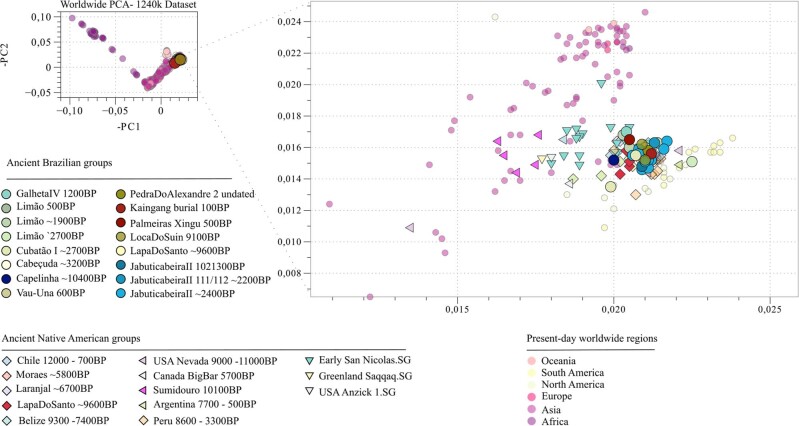
Genetic map of ancient and present-day individuals. Principal component analysis on the 1240k dataset where the principal components were calculated based on the genetic variation of modern-day worldwide populations onto which ancient samples were projected. All newly reported ancient individuals from Brazil fall into a cluster with previously published ancient genome-wide data from South America, except the Lapa do Sumidouro samples that are slightly displaced from the main cluster.

**Extended Data Fig. 2:**
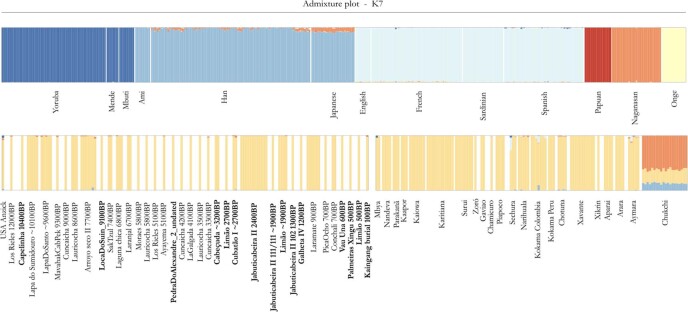
Genetic clustering of ancient and present-day individuals. Graphic representation of the clustering analysis performed with unsupervised ADMIXTURE at K = 7 on the Human Origins dataset. All newly reported ancient individuals from Brazil carry the same ancestry component as previously published ancient and modern genome-wide data from the Americas, without substantial levels of non-Native American ancestry.

To understand the genetic structure of pre-colonial Brazilian groups and assess their potential genetic transformations through time, we attempted to retrieve ancient DNA from 82 individuals from 24 archaeological sites across four regions: the southeastern and southern Atlantic coast, Lagoa Santa, the lower Amazon, and northeastern Brazil (Supplementary Information and Supplementary Data 1). After applying established criteria for ancient DNA authentication, we obtained a final dataset of genome-wide data from 34 individuals from 11 archaeological sites spanning the past ~10,000 years (Fig. 1 and Supplementary Data 1). We produced genome-wide data via in-solution capture by enriching for a targeted set of ~1.24 million single nucleotide polymorphisms (SNPs) across the human genome (1240k SNP capture)^67^. We also captured the entire mitochondrial genome (mtDNA) to assign mtDNA haplogroups and to estimate contamination levels, which were found to be low for all cases (<2%). Nuclear DNA contamination estimated for 20 male individuals on the basis of X-chromosome heterozygosity levels^68^ was also low (<3.5%). Principal component analysis (PCA) and a cluster analysis including worldwide populations further confirmed that all individuals fall within Native American genetic diversity (Extended Data Figs. 1 and 2). For population genetic analyses, we combined the newly authenticated ancient Brazilian genome-wide dataset with previously published ones^62,63^. Individuals were grouped on the basis of archaeological site, radiocarbon date and genetic affinities established through *f*_3_ outgroup statistics (Methods and Supplementary Data 1).

### Early Holocene hunter-gatherer radiation

**Extended Data Fig. 3:**
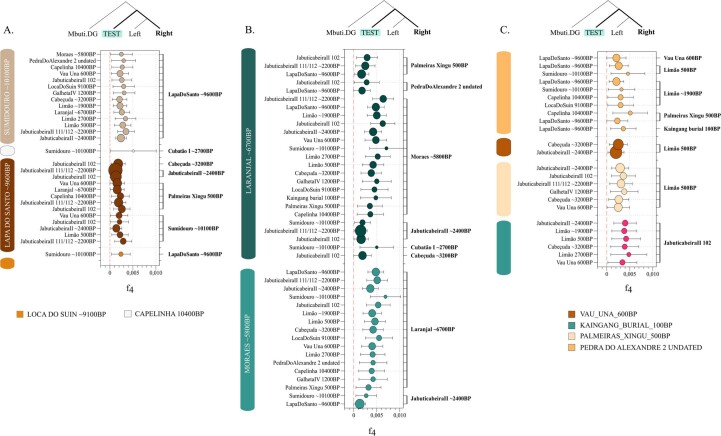
*f*_*4*_-statistics among ancient Brazilian groups/individuals from 10,000 BP to 100 BP. Graphical representation of the significant f_4_-statistics (Z-score >3) polarized to positive values performed on ancient Brazilian genome-wide data in the form *f*_*4*_ (Mbuti; TEST; Ancient Brazilian - left; Ancient Brazilian - right) to test allele sharing among the different groups using the 1240k dataset. In the sub-panels TEST refers to A) early Holocene individuals; B) middle Holocene riverine Sambaqui individuals; C) post-1,000 BP or undated individuals. The point sizes refer to the number of SNPs used to compute the f_4_ tests (at least 20,000 SNPs) and colors correspond to the TEST ancient group/individual. Bars represent f_4_-statistics +/− 3 standard errors for all tests. Figure related to Supplementary Data 2.

The oldest human presence in southeastern Brazil is directly attested by the ‘Luzio’ individual, a skeleton buried in the riverine shellmound of Capelinha genetically analysed here (Capelinha_10400BP). The morphological similarity of this male individual to paleoamerican features observed in early Holocene groups from the Lagoa Santa region, and the chronological gap of almost 3,000 years with other burials from the same site, call into question his association with riverine Sambaqui societies^10^. We investigated the genetic affinities of Capelinha_10400BP to other ancient Brazilian individuals using *f*_4_ statistics of the form *f*_4_(Mbuti, Capelinha_10400BP; ancient Brazilians—left, ancient Brazilians—right) (Extended Data Fig. 3a and Supplementary Data 2). None of the tested ancient individuals show a higher allele sharing with Capelinha_10400BP, even when the temporally close and phenotypically similar Lagoa Santa groups are considered. The same pattern is observed when Capelinha_10400BP is compared with an early Holocene hunter-gatherer from Loca do Suin, dated to ~9,100 yr bp and located 200 km southwest of the Lagoa Santa region (Loca do Suin_9100BP). Conversely, the Lapa do Santo_9600BP and Sumidouro_10100BP groups share a higher genetic affinity with each other than with any other ancient Brazilian group (significance considered at *Z* > |3|, if not differently indicated) (Extended Data Fig. 3a). These results indicate that Capelinha_10400BP does not represent an early occupation of the southeast coast by inland groups carrying Lagoa Santa–related ancestry and suggest that his population did not leave a substantial genetic contribution in the later Brazilian individuals analysed here.

We then used qpWave^69^ to estimate the minimum number of streams of ancestry necessary to explain the genetic variation observed among early Holocene hunter-gatherers across South America. Our results show that Capelinha_10400BP and Loca do Suin_9100BP cannot be distinguished from other early Holocene populations as part of a distinct wave of ancestry (*P* > 0.01) (Supplementary Data 3). To the limit of our resolution, the lack of close affinity among early Holocene individuals from different South American sites suggests that they derived from a rapid radiation event^63^.

**Extended Data Fig. 4:**
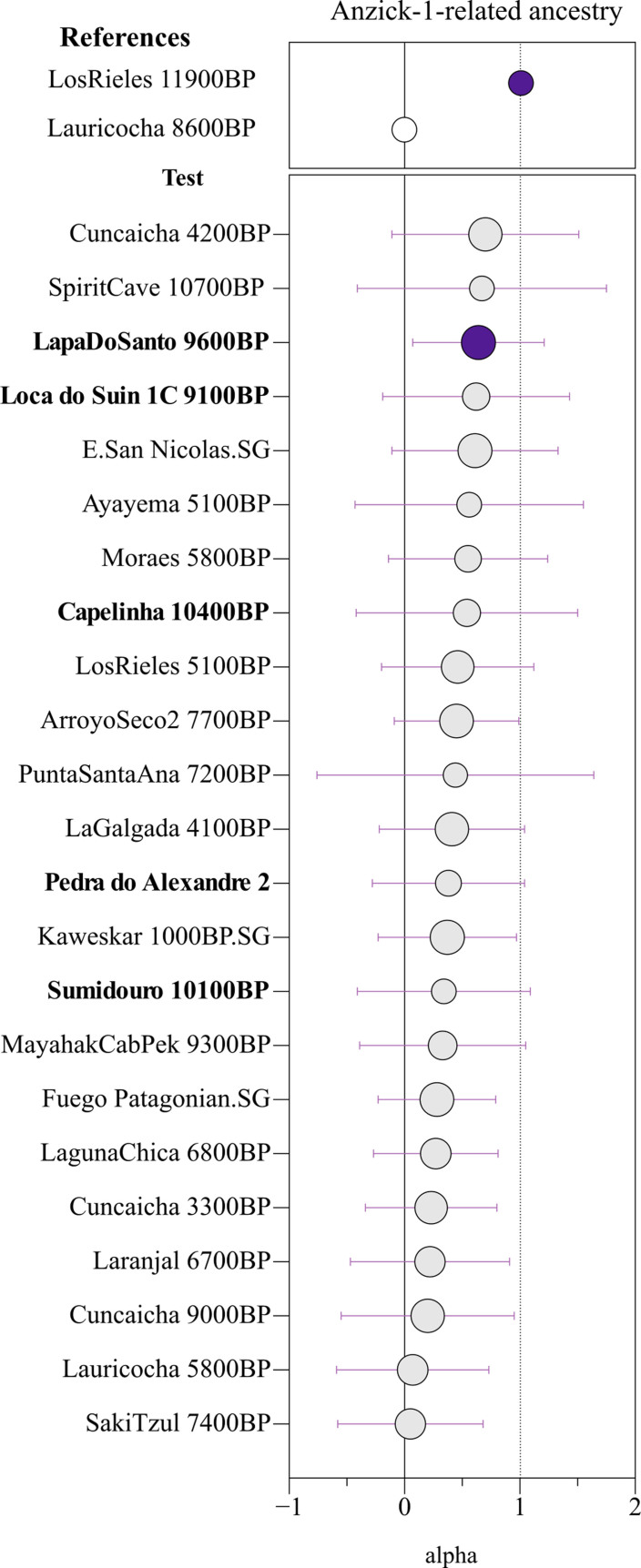
*F*_*4*_-ratio test for the *Anzick-1*-related ancestry. Indirect estimates of *Anzick-1*-related ancestry proportions setting *Los Rieles_11900BP* and *Lauricocha_8600BP* as the early Holocene reference individuals with the highest and lowest amount of *Anzick-1*-related ancestry, respectively. ‘Test’ refers to the ancient Native American individuals/groups included in the calculation and highlighted in bold are samples from Brazil. The alpha values were calculated using the *f4*-ratio formula reported in the Methods section. The size of the points represents the number of SNPs used to compute comparisons (at least 400,000 SNPs). Bars represent *f4*-ratio ± 3 standard errors for all tests. The *F*_*4*_ ratio estimates are sorted from the highest to the lowest alpha values. The *Lapa do Santo_9600BP* group (purple dot) shows a significantly higher *Anzick-1*-related ancestry than *Lauricocha_8600BP*.

A previous study also revealed that the oldest South American genomes, Los Rieles_11900BP from Chile and Lapa do Santo_9600BP from Brazil, carried a higher affinity to the Clovis-associated Anzick-1 individual from North America than Lauricocha_8600BP from Peru did^63^. With *f*_4_ statistics, we could show that while Capelinha_10400BP and Sumidouro_10100BP do not have a lower affinity to Anzick-1 than Los Rieles_11900BP and Lapa do Santo_9600BP do, they also do not show a higher affinity to Anzick-1 than Lauricocha_8600BP does (Supplementary Data 4). To measure the relative proportion of the Anzick-1-related ancestry in ancient South American groups, we performed an *f*_4_-ratio test^70^ (Methods), using Los Rieles_11900BP and Lauricocha_8600BP as the reference individuals with the maximum and minimum amount of such ancestry in early Holocene South America, respectively. Our results corroborate that Lapa do Santo_9600BP carry a significantly higher amount of Anzick-1-related ancestry than Lauricocha_8600BP (*Z* = 3.31), while the other tested groups show different proportions without reaching significance (Extended Data Fig. 4 and Supplementary Data 4). This trend suggests a genetic gradient of Anzick-1-related contribution in early South American hunter-gatherers rather than a scenario of two isolated migration waves with and without Anzick-1-related ancestry.

### Shellmound societies from the middle to the late Holocene

To investigate the affinities between riverine and coastal Sambaqui groups, we analysed our newly produced data alongside previously published individuals from the riverine sambaquis Laranjal (*n* = 2, ~6,700 yr bp) and Moraes (*n* = 1, ~5,800 yr bp)^63^ (Extended Data Fig. 3b). The southeast coast shellmounds are represented by the sambaqui do Limão (*n* = 6, ~2,700–500 yr bp), located in the State of Espírito Santo. The south coast Sambaqui are represented by individuals from three shellmounds—Jabuticabeira II (*n* = 17, ~2,500–1,300 yr bp), Cabeçuda (*n* = 2, ~3,200 yr bp) and Cubatão I (*n* = 2, ~2,700–2,600 yr bp)—and one individual from the fishmound Galheta IV (~1,200 yr bp), representing the final horizon of Sambaqui societies.

Our analyses confirm the strong local genetic affinity between the riverine Sambaqui individuals compared with all other ancient Brazilian groups in our dataset (*f*_4_(Mbuti, Laranjal_6700BP; ancient Brazilian group, Moraes_5800BP) > 0). Individuals from the riverine sites also show genetic similarities to individuals from the southern coastal sambaquis of Cubatão I (CubatãoI_2700BP), Cabeçuda (Cabeçuda_3200BP) and Jabuticabeira II (JabuticabeiraII_~2400BP), indicating some level of genetic continuity through time between riverine shellmound builders and Sambaqui societies from the southern coast. Interestingly, this genetic similarity is not observed between the riverine shellmounds and the sambaqui do Limão, located further north (Fig. 1 and Extended Data Fig. 3b).

To improve our knowledge on the genetic interactions among Sambaqui groups, we co-analysed all individuals from the five coastal sites, which are located up to 1,500 km apart along the southeast and south coasts (Fig. 1). The archaeological site with the largest number of analysed genome-wide data is Jabuticabeira II. The 17 individuals from this site cluster in three genetically distinct groups, as revealed through *f*_3_ and *f*_4_ tests (Supplementary Data 2): (1) a main cluster, composed of 14 individuals dated to ~2,500–2,300 yr bp (JabuticabeiraII_~2400BP—we identify genetic groups by rounding the mean calibrated age for all dated individuals; Supplementary Data 1), of which 12 are not first degree related and are grouped together for analysis; (2) two first-degree-related individuals dated to ~2,200–2,100 yr bp (JabuticabeiraII_111/112_~2200BP, only one individual used for analysis); and (3) the most recent individual, dated to ~1,300 yr bp (JabuticabeiraII_102_1300BP). This skeleton was found in the topmost shell deposit and exhibited a different funerary pattern from the older burials, including an extended rather than a flexed position and the absence of grave goods (Supplementary Data 1). In *f*_4_ statistics, we found a higher genetic affinity between the three groups from Jabuticabeira II compared with all other ancient Brazilian groups (Supplementary Data 2). The temporally intermediate individual appears to be genetically intermediate to the preceding and succeeding individuals, as indicated by *f*_4_(Mbuti, JabuticabeiraII_111/112_~2200BP; JabuticabeiraII_~2400, JabuticabeiraII_1300BP) ~ 0 (*Z* = 0.47).

**Fig. 2:**
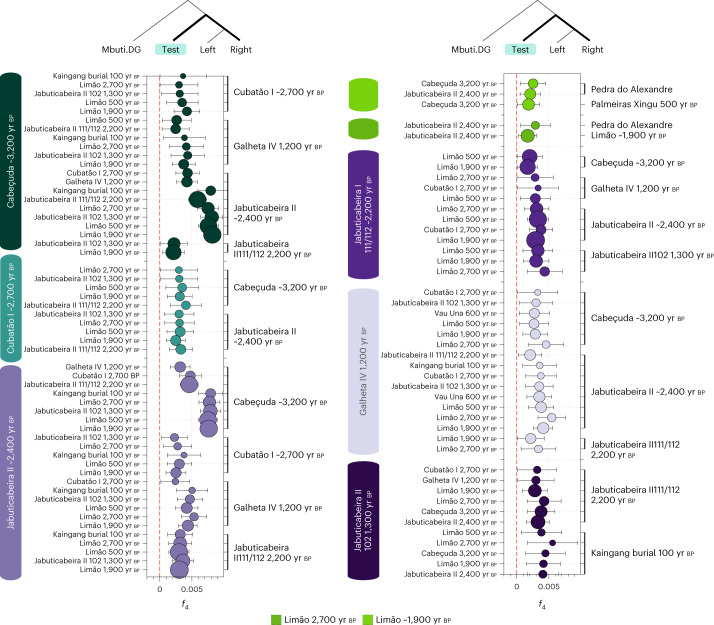
*f*_4_ statistics among ancient Brazilian groups and/or individuals from the southern and southeastern coasts dated between 3,000 yr bp and 1,000 yr bp. Significant *f*_4_ statistics (*Z* > 3) polarized to positive values performed on ancient Brazilian genome-wide data in the form *f*_4_(Mbuti, TEST; ancient Brazilians—left, ancient Brazilians—right) to test allele sharing among the different groups using the 1240k dataset. The point sizes refer to the number of SNPs used to compute the *f*_4_ tests (at least 20,000 SNPs), and the colours correspond to the TEST group/individual. The bars represent *f*_4_ statistics ± 3 standard errors. Figure related to Supplementary Data 2.

Intersite comparisons showed higher allele sharing between the JabuticabeiraII_~2400BP group, JabuticabeiraII_111/112_~2200BP, Cabeçuda_3200BP and GalhetaIV_1200BP, to the exclusion of other ancient Brazilian groups. The CubatãoI_~2700BP group shows genetic connections to the other southern shellmound groups such as JabuticabeiraII_~2400BP and Cabeçuda_3200BP. The affinities between these four shellmound and fishmound individuals thus reveal the presence of a late Holocene genetic cluster in the southern coast of Brazil (Fig. 2).

On the southeastern coast, the genetic similarities ascertained through *f*_3_ outgroup and *f*_4_ statistics revealed three distinct groups at the sambaqui do Limão: (1) the oldest individual (Limão_2700BP), (2) a cluster of four temporally intermediate individuals (Limão_~1900BP) and (3) the most recent individual (Limão_500BP). The Limão_~1900BP group shows the highest genetic affinities to Limão_2700BP and to a possibly early to middle Holocene hunter-gatherer from the northeastern site of Pedra do Alexandre (Pedra Do Alexandre2_undated)^71^ (Fig. 2). This result demonstrates genetic connections between Sambaqui individuals from the southeast coast and hunter-gatherer groups from northeastern Brazil. When combined with the results obtained from the south coast sambaquis, our analyses indicate that shellmound societies from the south and southeast (that is, Santa Catarina and Espírito Santo states, respectively) do not constitute a genetically homogenous population, as previously suggested by the analyses of cranial and dental morphological variation^21,23^.

### The final horizons of shellmound societies

The significance of Taquara-Itararé ceramics (associated with proto-Jê speakers) at coastal sites after the final horizon of sambaqui construction has been at the centre of recent academic debates. According to some scholars, an intensification in contacts with proto-Jê-speaking groups after ~2,000 yr bp, even before the appearance of ceramics at the coast, would have led to the demise of Sambaqui societies^9^^,11,30^. In this work, the post-2,000 yr bp horizon is represented by JabuticabeiraII_102_1300BP (Fig. 2), buried at the top of the shell deposit, and by an individual from Galheta IV, a fishmound with Taquara-Itararé pottery (GalhetaIV_1200BP) (Fig. 2). To further investigate the genetic connections between individuals in sambaquis and fishmounds, proto-Jê-speaking groups, and present-day indigenous peoples, we merged our ancient genomic data with two published present-day genomic datasets: (1) the Illumina dataset assembled in Reich et al.^72^, combined with 1240k SNP capture data generated in this study from an early twentieth-century southeastern Kaingang individual from the state of São Paulo (Kaingang burial_100BP), showing distinctive affinity with present-day southern Kaingang; and (2) the Human Origins dataset^54,66,69,73^.

**Fig. 3:**
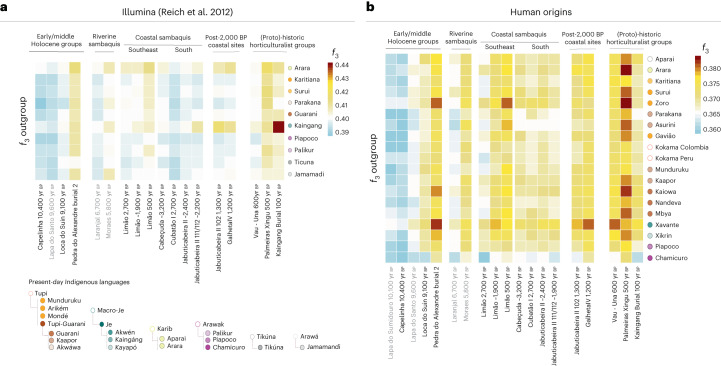
Heat map of *f*_3_ outgroup statistics. **a**, Comparisons between ancient and present-day groups/individuals using the Illumina dataset with the statistics *f*_3_(present-day indigenous groups Brazil, ancient Brazilians; Mbuti). **b**, Same statistics as in **a** but using the Human Origins dataset. In both heat maps, warmer colours represent higher genetic affinities while cooler colours represent lower genetic affinities. The dot colours indicate the languages of the tested present-day populations, as shown in the bottom-left legend.

**Extended Data Fig. 5:**
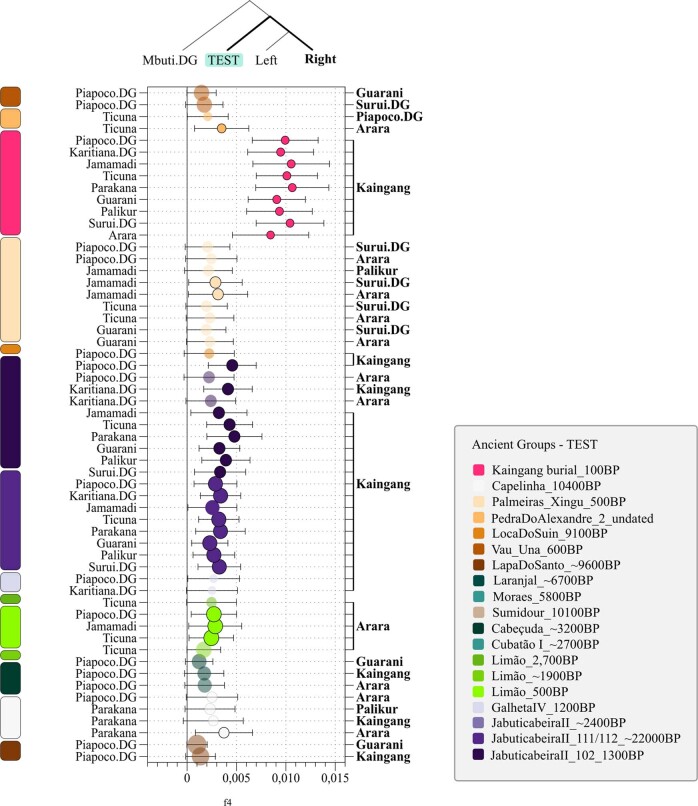
*f*_*4*_-statistics between ancient Brazilian groups/individuals and present-day South American populations. Graphical representation of the f_4_-statistics polarized to positive values (Z-score> 2.5) performed on the ancient Brazilians genome-wide data and present-day indigenous groups from South America using SNPs overlapping with the Illumina dataset - f_4_ (Mbuti; TEST; present-day indigenous groups - left, present-day indigenous group - right). Size of the points represents the number of SNPs used to compute comparisons (at least 20,000 SNPs), points with black outlines indicate tests with Z > 3 and colors correspond to the TEST ancient group/individual. Bars represent f_4_-statistics ± 3 standard errors. Figure related to Supplementary Data 5.

Using the Illumina dataset, we observed patterns of shared genetic drift between some Sambaqui groups and present-day Kaingang (Fig. 3a and Extended Data Fig. 5). To formally test this affinity, we performed the following *f*_4_ tests: (1) *f*_4_(Mbuti, ancient coastal group; Kaingang, other present-day indigenous groups) and (2) *f*_4_(Mbuti, Kaingang; ancient coastal group A, ancient coastal group B). The results from the first test reveal an excess of genetic similarity between present-day Kaingang and JabuticabeiraII_102_1300BP. The second test expands this finding by showing that JabuticaberiaII_111/112_~2200BP and even more so JabuticabeiraII_102_1300BP are genetically closer to present-day and twentieth-century Kaingang, when compared with not only the JabuticabeiraII_~2400BP group but also the Taquara-Itararé-pottery-associated GalhetaIV_1200BP individual (Supplementary Data 5 and Extended Data Fig. 5). This genetic link between Kaingang and the younger Jabuticabeira II individuals corroborates the hypothesis of an intensification of contacts between proto-Jê-speaking groups and Sambaqui societies of the southern coast, at least from ~2,200 yr bp.

The analysis of the stable isotope ratio ^87^Sr/^86^Sr in the tooth enamel of JabuticabeiraII_102_1300BP (0.7111) also points at a different provenance for this female individual, possibly from another coastal location, when compared with the JabuticabeiraII_~2400BP group (0.7095 ± 0.000096; *n* = 7) (Supplementary Table 16). This could also indicate a dietary change, since a mixed marine and C_3_-resource diet has already been described for JabuticabeiraII_102_1300BP, in contrast to the high marine protein intake of older individuals^74^. Instead, the absence of a distinctive Jê-related signal in GalhetaIV_1200BP, considered to be the typical Jê site on the coast, points at a certain level of demic continuity with Sambaqui groups after the arrival of ceramics and the end of shellmound construction. Therefore, it suggests that cultural diffusion might have also been an important mechanism in the spread of ceramics across the Atlantic coast of Brazil, as indicated by previous studies^2,21,23,75^.

**Fig. 4:**
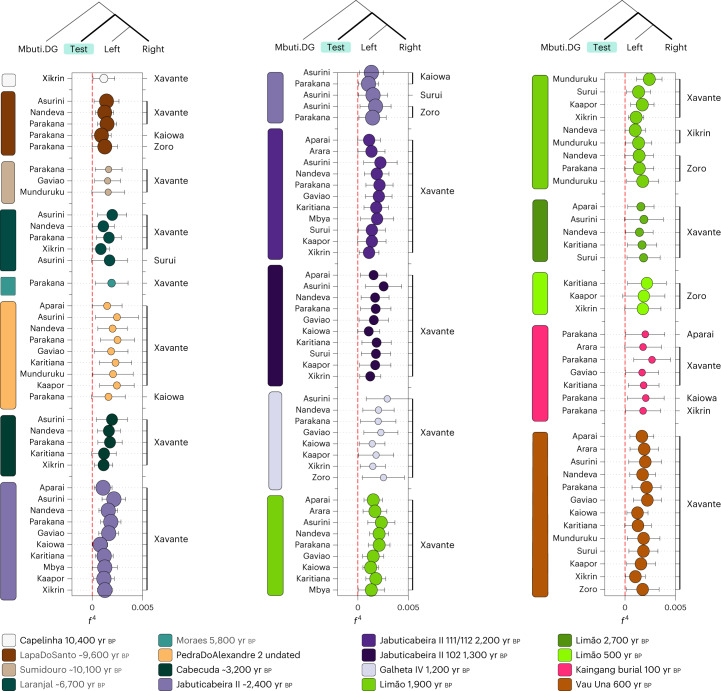
*f*_4_ statistics between ancient groups/individuals and present-day Brazilian groups. The *f*_4_ statistics polarized to positive values (*Z* > 3) performed on ancient Brazilian genome-wide data and present-day indigenous groups using the Human Origins dataset for *f*_4_(Mbuti, TEST; present-day indigenous groups Brazil—left, present-day indigenous group Brazil—right). The point sizes represent the number of SNPs used to compute the *f*_4_ tests (at least 20,000 SNPs). The colours correspond to the TEST ancient group/individual. The bars represent *f*_4_ statistics ± 3 standard errors. Figure related to Supplementary Data 5.

With the Human Origins dataset, we first expanded the previous findings using *f*_3_ outgroup statistics (Fig. 3b). Moreover, *f*_4_ statistics of the form *f*_4_(Mbuti, Brazilian ancient groups; present-day indigenous groups—left, present-day indigenous groups—right) revealed that all Sambaqui individuals show a significant genetic attraction to the Xavánte (Jê-speaking) in contrast to the other available indigenous populations (Fig. 4). To investigate whether the influence of Jê-related ancestry in the Sambaqui individuals from the southern coast can be attributed specifically to either Kaingang or Xavánte, we performed the test *f*_4_(Tanzania_3000BP, Sambaqui groups; Xavánte, Kaingang_burial_100BP) (Supplementary Data 5). Here we used ancient African individuals^73^ to mitigate biases due to attraction between ancient DNA samples. Our results show that all Jabuticabeira II individuals are equally associated with both tested sources of Jê ancestry (Kaingang and Xavánte) (|Z| < 1.71). This suggests that the specific Jê-related ancestry contributing to southern Sambaqui groups is missing in our ancient and present-day genetic dataset. More genomic data from other Jê-speaking groups are needed to accurately assign a specific genetic contribution.

The Limão_~1900BP individuals also show genetic affinity to the present-day Jê-speaking groups from central Brazil (Xavánte) when compared with other linguistic families, such as Karib (Arara and Aparai) or Tupi (Mondé, Arikém and Tupi-Guarani). Interestingly, we observed a genetic link between the latest burial at the site, Limão_500BP, and the Zoró (a population related to the Tupi-Mondé language) in comparison to other present-day Tupi-speaking peoples (that is, Nandeva, Gavião, Karitiana and Parakanã) (Fig. 4 and Supplementary Data 5 and 6). This specific affinity might represent the first direct genetic evidence for the arrival on the southeast coast of Tupi-Guarani speakers, who are thought to have originated in southeast Amazonia^54,55,59^. While we cannot determine the exact arrival time of this ancestry, its absence in the older groups from sambaqui do Limão (Limão_2700BP and Limão_~1900BP) indicates that it occurred after the initial settlement of the site by Sambaqui groups.

### Links with ceramists from Amazonia and northeastern Brazil

To investigate the chronological depth of the shared ancestry between Sambaqui and Jê-, Tupi- and Karib-speaking groups, we sequenced individuals from late Holocene archaeological sites in the Cerrado of northeastern Brazil and the lower Amazon Forest. The former is associated with the Una tradition (Vau_Una_600BP), a ceramic type made by horticulturists that occupied a vast territory in central and northeastern Brazil^76^, and the latter is associated with the Koriabo tradition (Palmeiras Xingu_500BP), a late pre-colonial/early colonial archaeological culture (~1,200–1,600 CE) that may represent the southernmost Karib expansion in South America^57,59,62,63^.

The genetic patterns obtained by performing *f*_4_ tests on Vau_Una_600BP and present-day Native American populations from the Human Origins dataset show strong evidence of genetic similarities between the Una-context individual and Xavánte, when compared with Tupi (Tupi Mondé, Arikén and Tupi-Guarani) and Karib populations (Arara and Apalai) (Fig. 4). This provides direct evidence for the association of Jê-speaking populations with pottery makers of the Una tradition. The results of the *f*_4_ test performed on Brazilian indigenous populations included in the Illumina dataset show that Palmeiras Xingu_500BP shares genetic affinities with Arara, a Karib-speaking group from the lower Amazon, and with the Tupi-speaking Surui Paiter (Extended Data Fig. 5).

In comparison to all Sambaqui individuals analysed here, both Vau_Una_600BP and Palmeiras Xingu_500BP present a genetic attraction to the latest burial from the sambaqui do Limão (Limão_500BP), indicating some level of shared genetic drift in the most recent past.

### The Population Y signal

We investigated the presence of the Population Y signal in the newly produced data with *f*_4_ statistics of the form *f*_4_(Mbuti, Papuan/Onge/Australian; present-day Mexicans, ancient Brazilians)^65^. The only ancient Brazilian group showing significant affinity to Onge, compared with present-day Mexicans, is the JabuticabeiraII_~2400BP group. The signal is mainly driven by one individual (JBT009—burial 38), but it remains for the entire group even after the exclusion of JBT009. Similarly, there is significant genetic attraction between Onge and one individual from the Cabeçuda_3200BP group (CBE004—burial 15), while all other tests do not reach values close to significance (Supplementary Data 7). However, no evidence of the Population Y signal is found in the recent Amazonian individual Palmeiras Xingu_500BP, despite the fact that this ancestry was first described in present-day Amazonian populations; or in Capelinha_10400BP, despite its association with the paleoamerican cranial morphology^10,65^. We further tested the presence of differential affinity of ancient Brazilian individuals to present-day Papuans, Onge and Australians, as well as the 40,000-year-old Tianyuan genome-wide data from China^77^ using *f*_4_ statistics of the form *f*_4_(Mbuti, Papuan/Onge/Australian/Tianyuan; Ancient Brazilian A, Ancient Brazilian B). Only the JabuticabeiraII_~2400BP group reaches significant attraction to both Onge and Papuans, and only in comparison to LapaDoSanto_9600BP (ref. ^63^). This suggests either that the Population Y signal is equally widespread in most tested ancient individuals from Brazil or that previously reported attractions to non-American ancestries^62,77,78^ are exacerbated by the use of present-day Mexican populations in comparison to ancient groups (Supplementary Data 7).

### Uniparental markers, genetic diversity and runs of homozygosity

All males in our dataset belong to Y-chromosome haplogroup Q1b, which has the highest frequency in present-day South Americans. To the limit of the available SNP coverage, the male individuals from Jabuticabeira II carry either the common haplogroup Q1b1a1a-M3 or the currently rare haplogroup Q1a2a1b-CTS1780, confirming its higher frequency in ancient South Americans^63^ (Supplementary Data 1).

**Extended Data Fig. 6:**
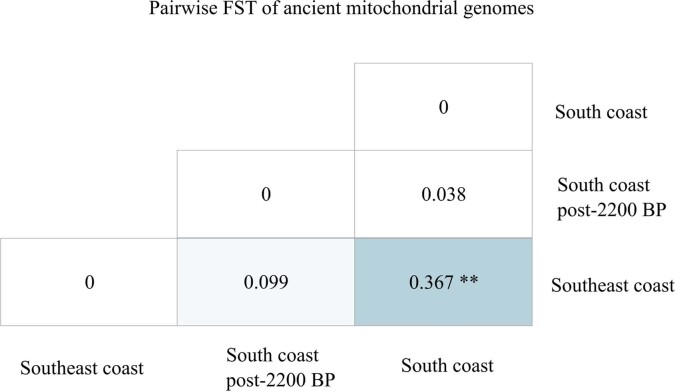
Heatmap of mtDNA pairwise genetic differentiation (FST) among coastal Sambaqui groups. The stars (**) represents a *p*-value < 0.01 in the Amova test. Sambaqui individuals are grouped into south coast, southeast coast, and 2,200-1,200 BP south coast.

The mtDNA analysis shows that all newly studied individuals belong to American-specific mtDNA haplogroups (A2, B2, C1b, C1c, C1d1 and D1) (Supplementary Data 1). An exception is individual Loca Do Suin_9100BP, who carries the extremely rare and primarily North American mtDNA haplogroup C4c. Finding this mtDNA lineage in Brazil during the early Holocene provides additional support to the possibility that haplogroup C4c entered the Americas during early peopling events^79^. On the basis of the mtDNA diversity, we tested the presence of sub-structure among Sambaqui groups. Our results show a level of differentiation between Sambaqui individuals from the south coast and those from the southeast coast (Extended Data Fig. 6).

**Fig. 5:**
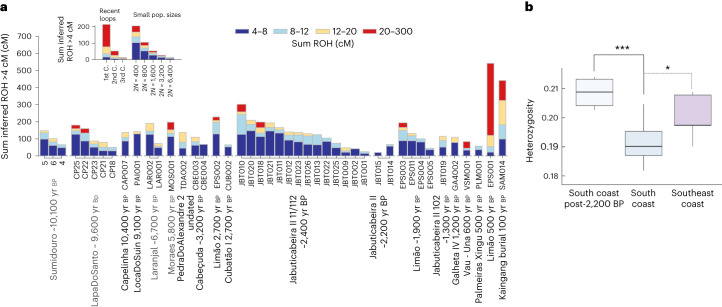
ROH profiles and heterozygosity of the ancient coastal groups. **a**, Sum of ROH fragments higher than 4 cM for each individual with more than 190,000 SNPs sorted by population name and in chronological order. The insert provides a legend of individual ROH profiles for recent loops (parents from 1st to 3rd cousins (C.)) and small population sizes. **b**, Heterozygosity distribution among the tested groups. This was calculated on the basis of the pseudo-diploid genotypes of three Sambaqui groups: south coast (*n* = 17), southeast coast (*n* = 5) and 2,200–1,200 yr bp individuals from the south coast (*n* = 4). In the box plots, the central line represents the median, the box edges represent the 25th and 75th percentiles, and the whiskers show the distribution of the remaining variation. The connectors mark the significant results obtained with the non-parametric Kruskal–Wallis test (*P* = 0.001), followed by the post hoc Conover’s test for multiple comparisons using the false discovery rate correction method (*P = 0.01914; ***P = 0.00089).

**Extended Data Fig. 7:**
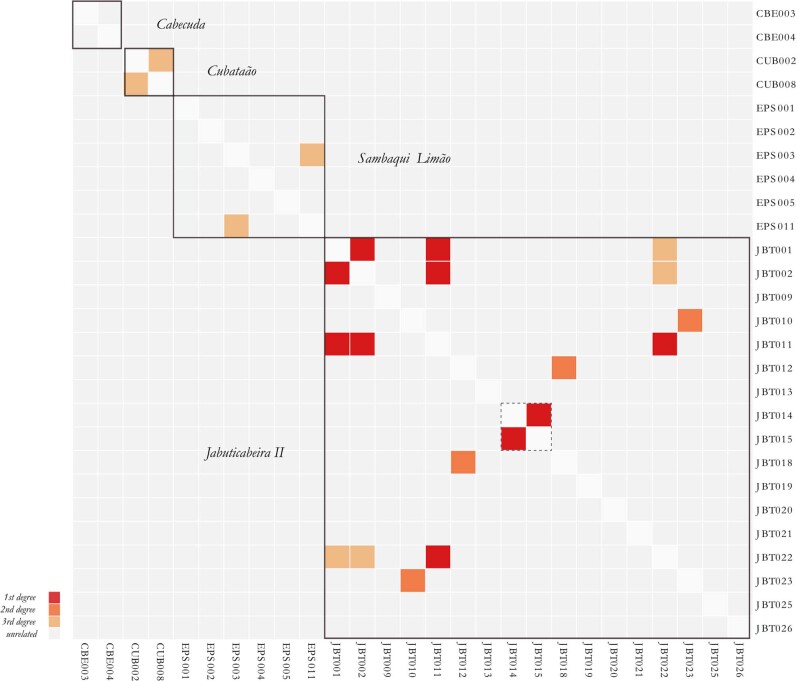
Biological kinship between Sambaqui individuals. Estimates of relatedness based on genome-wide pairwise mismatch rate (PMR). At the site Jabuticabeira II a total of five first-degree, two second-degree and two third-degree kinships were identified. Figure related to Supplementary Data 9.

At the Jabuticabeira II site, 16 individuals share the same mtDNA haplogroup C1c with a maximum of one nucleotide distance among all mtDNA sequences. The only exception is represented by JabuticabeiraII_102_1300BP, who carries mtDNA haplogroup B2 (ref. ^63^). This pattern of uniparental markers, considered alongside the generally low pairwise mismatch rate, could be compatible with a scenario of consanguinity among Jabuticabeira II individuals (Extended Data Fig. 7). To test this, we calculated runs of homozygosity (ROH)^80^. Those results revealed a large number of short ROH (4–8 cM) in the JabuticabeiraII_~2400BP group, suggesting a smaller effective population size (2*n* of ~400 to ~1,600 individuals contributing to the next generation) than for younger burials from the same site (Fig. 5a). Therefore, rather than recent consanguinity, this genetic pattern is consistent with a bottlenecked population and calls into question the expectation of large demography in Sambaqui societies. Studies of pre-contact subsistence fisheries using data from the Cubatão I site have also indicated a lower-than-expected population size among southern Sambaqui groups^81^. Contemporaneous individuals from the sambaqui do Limão present a similar ROH profile, while the Limao_500BP individual shows a pattern consistent with first-cousin consanguinity (Fig. 5a).

Finally, the south coast Sambaqui groups (JabuticabeiraII_~2400BP, Cabeçuda_3200BP and CubatãoI_~2700BP) show lower heterozygosity levels than those at the southeast coast site (sambaqui do Limão) and even lower than late Sambaqui individuals from the south coast (JabuticabeiraII_111/112_~2200BP, JabuticabeiraII_102_1300BP and GalhetaIV_1200BP) (Fig. 5b). The increase in heterozygosity through time in southern Sambaqui groups is probably associated with gene flow of Jê-related ancestry from the inland detected here by 2,200 yr bp.

## Discussion

The oldest individual newly sequenced in this study, Capelinha_10400BP, does not carry a distinct genetic similarity to any other early Holocene or younger populations but shows a generalized affinity to ancient Brazilian and present-day South American groups. This suggests that his source population had a basal placement among the initial radiation event into South America. Moreover, both Capelinha_10400BP and Sumidouro_10100BP lack a significant affinity to Anzick-1-related ancestry (Supplementary Data 4 and Extended Data Fig. 4). These individuals predate by more than a thousand years the earliest occurrence of South American individuals without evidence of this ancestry (Cuncaicha_9000BP and Lauricocha_8600BP), challenging the scenario of two subsequent waves of expansion into South America, the first one with and the second one without Anzick-1-related ancestry^63^. However, we caution that this result could be affected by the lack of statistical power, and another potential scenario would involve early South American settlers carrying different proportions of this genetic component. Additional genomes from other regions of South America would be necessary to assess whether populations carrying Anzick-1-related ancestry were replaced by or intermixed with other early Holocene groups.

**Extended Data Fig. 8:**
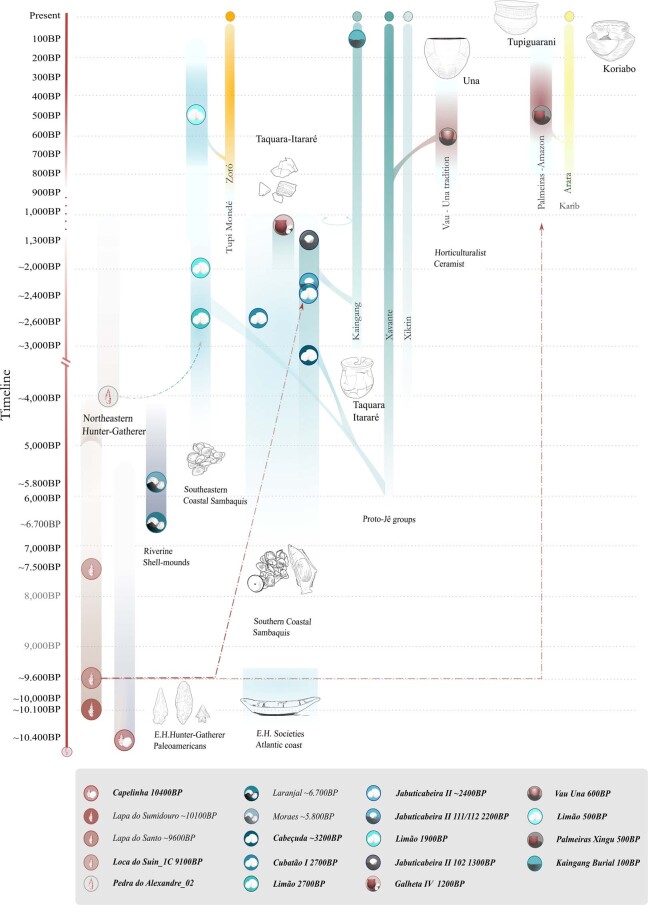
Summary of the genetic affinities reconstructed through the analysis of genome-wide data from ancient Brazilian groups. The shaded vertical bars indicate ancestries represented by site-related symbols that are ordered chronologically in the legend. Genetic connections between groups/individuals are represented by shaded lines. Dashed lines indicate the extra affinity between the ancestry found in *Lapa do Santo_9600BP* and *Pedra Do Alexandre2_undated* and other ancient groups. The archaeological and radiocarbon dates for the genetically analysed individuals are indicated in the timeline on the left.

The genetic distinctiveness between early Holocene individuals from the Lagoa Santa region, Capelinha_10400BP and Loca Do Suin_9100BP, also indicates greater genetic variation among early Brazilian hunter-gatherers than previously expected. Within the Lagoa Santa region, early Holocene individuals mostly derived from a common ancestral group, as shown by the high genetic affinity between the Sumidouro_10100BP and Lapa do Santo_9600BP groups. We also detected two distinct genetic attractions between Lapa do Santo_9600BP and late Holocene groups. The first signal was observed with the southern Sambaqui JabuticabeiraII_~2400BP group and Cabeçuda_3200BP, and the second with the Amazonian individual Palmeiras Xingu_500BP (Extended Data Fig. 3a). The genetic connection between individuals separated by thousands of kilometres and thousands of years might indicate the survival of this ancestry through time (Extended Data Fig. 8).

The Population Y signal related to Andamanese and Australasian populations could not be detected in the early Holocene Capelinha_10400BP individual or in the Amazonian Palmeiras Xingu_500BP individual. However, we report this signal in individuals from the southern sambaqui sites of Cabeçuda_3200BP and JabuticabeiraII_~2400BP. The latter is the only pre-colonial group exhibiting higher affinity to non-American ancestries even in direct comparison to another ancient Brazilian group (Supplementary Data 7). If confirmed, the sporadic identification of the Population Y signal in ancient individuals with different ancestries, locations and time periods across Brazil—where this signal was first described—suggests a higher probability that it derives from genetic structure in the founding Native American population^65,77^ than from multiple independent migrations into the Americas^62,82^.

Middle Holocene riverine Sambaqui individuals (Laranjal_6700BP and Moraes_5800BP) are strongly related, confirming a local genetic structure^63^, which might correspond to a distinct genetic group when compared with coastal Sambaqui populations. Individuals from Laranjal and Moraes also show a higher affinity with south coast than with southeast coast Sambaqui groups, suggesting potential genetic links between geographically closer populations. However, the two sites represent only a small portion of the riverine sambaquis, and additional individuals should be genetically analysed to confirm this pattern.

**Extended Data Fig. 9:**
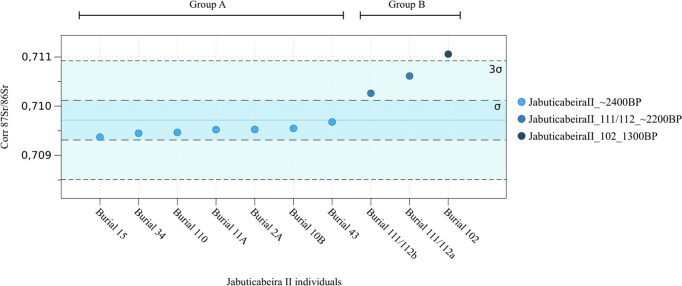
Strontium isotopic signature of individuals from the Jabuticabeira II site. Estimates of local origin based on ^87^Sr/^86^Sr values. We performed the Wilcoxon (two-sided) and t tests (two-sided) to investigate the significance of the differences between Group A (JabuticabeiraII_~2400BP burials) and Group B (Jabuticabeira II burials post-2200 BP). *p*-values = 0.017 and 0.036, respectively. σ and 3σ refer to 1 and 3 standard deviations from the mean of Group A.

The coastal Sambaqui groups Cabeçuda_3200BP and JabuticabeiraII_~2400BP showed high genetic affinity with each other (Fig. 2 and Supplementary Data 2). Both sites, only 20 km apart, exhibit genetic similarities to contemporaneous individuals from Cubatão I, about 200 km further north. The late burials from Jabuticabeira II (~2,200 yr bp and ~1,300 yr bp) display an incremental genetic attraction to southern Jê ancestry represented by both recent and present-day Kaingang (Figs. 2b and 3a, Extended Data Fig. 5 and Supplementary Data 5). JabuticabeiraII_102_1300BP has an ^87^Sr/^86^Sr isotope ratio above the range observed for older individuals at Jabuticabeira II (Extended Data Fig. 9) and could thus be a non-local individual who spent earlier years in continental areas (that is, the Santa Catarina highlands) or in a different location on the coast^45^. The presence of a non-local individual after 2,000 yr bp coincides with changes in the post-marital residence patterns^47^ and with dietary changes revealed by isotopic analyses^44,83^. The strong genetic affinity between Kaingang and JabuticabeiraII_102_1300BP demonstrates a genetic relationship between proto-Jê groups from the southern Brazilian highlands and post-2,000 yr bp coastal groups. However, this evidence precedes the arrival of Taquara-Itararé ceramics on the coast by around a hundred years^39^. Considering that Kaingang ancestry is already detected in Sambaqui individuals before the 2,000 yr bp horizon of cultural change, as indicated by JabuticabeiraII_111/112_~2200BP, our results show that the intensification of contacts between inland and coastal populations was concomitant with a sharp decline in shellmound construction^39^ and shortly before the appearance of fishmounds. This indicates that cultural contacts associated with genetic interactions at a time of unprecedented environmental and ecological changes may have influenced the end of shellmound architecture. Our results also show that one individual from Galheta IV (Galheta IV_1200BP), a fishmound with Taquara-Itararé ceramics, is genetically similar to the JabuticabeiraII_~2400BP group and Cabeçuda_3200BP. This suggests some level of demic continuity after the arrival of ceramics in the region (Fig. 2 and Supplementary Data 2 and 5).

On the southeast coast, the sambaqui do Limão individuals carry at least two distinct genetic ancestries. The Limão_2700BP individual and the Limão_~1900BP group show a significant affinity to the northeastern hunter-gatherer from Pedra do Alexandre2_undated and to the Amazonian individual Palmeiras Xingu_500BP. Despite cultural similarities, we do not observe an extra genetic affinity between individuals from the sambaqui do Limão and sambaqui sites on the southern coast (Fig. 2 and Extended Data Fig. 3c). The genetic link between the older sambaqui do Limão individuals and hunter-gatherers from northeast Brazil as well as present-day Xávante from central Brazil may explain their separation from contemporaneous groups on the southern coast. Furthermore, the high affinity of Limão_500BP with Tupi-speaking Zoro provides the first ancient genomic evidence for the spread of Tupi-related ancestry to the Brazilian southeast coast. The Tupi-Guarani expansion from southeastern Amazonia across the Atlantic coast of Brazil is a well-known demographic phenomenon^38,42,53–57^, and our results reveal an arrival of Tupi-related ancestry on the coast of Espírito Santo by at least 500 yr bp (Supplementary Data 6).

In conclusion, our results demonstrate that Sambaqui societies from the south and southeast coasts were not a genetically homogenous population. Both regions had different demographic trajectories, possibly due to the low mobility of coastal groups^2,21,29^. This contrasts with the cultural similarities described in the archaeological record and highlights the need to perform more regional and micro-scale studies to improve our understanding of the genomic history of eastern South America.

## Methods

### Archaeological sampling and ethical aspects

Permits for exporting the material for ancient DNA analysis were obtained from the Instituto do Patrimônio Histórico e Artístico Nacional, and sampling access was granted by the local curators at the following housing institutions: Museu de Arqueologia e Etnologia da Universidade de São Paulo (MAE-USP), Instituto de Biociências da Universidade de São Paulo, Superintendência no Espírito Santo do Instituto do Patrimônio Histórico e Artístico Nacional, Universidade Federal do Amapá, Museu Amazônico da Universidade Federal do Amazonas, Museu Paraense Emílio Goeldi, Scientia Consultoria Científica, Museu de Arqueologia do Xingó da Universidade Federal de Sergipe, Museu Arqueológico do Carste do Alto São Francisco, Grupo de Pesquisa em Educação Patrimonial e Arqueologia, Instituto Goiano de Pré-História e Antropologia da Pontifícia Universidade Católica de Goiás, Museu Histórico de Lins, and Universidade Federal de Pernambuco.

For the early twentieth-century sample originating from a Kaingang funerary context (Kaingang_burial_100BP), we reached out for approval to the indigenous community at TI Vanuíre, an Indigenous Land recognized by the 1988 Brazilian Constitution that is located ~65 km from the archaeological burial mound (Supplementary Information, ‘Kaingang’). The Kaingang spiritual leader who was in charge of our solicitation requested two members of our research group to engage in dialogue with the Kaingang community, including a slide presentation detailing all aspects of the present study. After internal community consultation, the research group members were informed that the data generated from the Kaingang sample could be included in the present study. The contact of our research group with the Kaingang community was mediated by M. X. Cury (MAE-USP), an expert in decolonizing curatorial processes in Brazilian museums.

The present study is part of a collaborative agreement between the Max Planck Institute for Evolutionary Anthropology (MPI) and the University of São Paulo. The collaboration includes the training of Brazilian students by the MPI staff in techniques of extraction and analysis of ancient DNA. The agreement also includes the establishment of an ancient DNA laboratory at USP under the technical guidance of the MPI and financed by the Fundação de Amparo à Pesquisa do Estado de São Paulo. A Max Planck Partner Group was established by A. Strauss to fund early career researchers working in the ancient DNA laboratory at USP.

### Ancient DNA processing

All human skeletal elements used in this study were introduced into the clean room facilities at the Max Planck Institute for the Science of Human History in Jena, Germany. The material was photographed and stored in new plastic bags. Petrous portions of the temporal bone and teeth were exposed for one hour to ultraviolet radiation on both sides to reduce surface DNA contamination before any sampling procedures were performed. Between 28 and 60 mg of tooth or bone powder were obtained. Teeth were cut along the enamel–dentin junction and drilled into the pulp chamber of the crown using a dentist drill rotated at low speed. Petrous bones were sampled following the protocol described in Pinhasi et al.^84^. We sampled 82 skeletal elements from 24 sites: Capelinha (4), Cabeçuda (4), Cubatão I (5), Sambaqui do Limão (11), Estreito (1), Galheta IV (5), Hatahara (1), Jabuticabeira II (21), Jêrimum (2), Justino (4), Lapa do Santo (2), Laranjal (2), Loca do Suin (4), Moraes (3), Palmeiras-Xingu (2), Pavão XVI (1), São José II (1), Pedra do Alexandre (2), Marajoara Anthropomorphic Urn t-8 (1), Gruta das Caretas (1), Marabaixo-Macapá (AP) (1), Ramuse Nóbrega (GO-RS-01) (1), Kaingang burials (2) and Vau-Una (1) (Supplementary Data 1).

### DNA extraction and library preparation

The collected bone/dentin powders were digested using 25 μl of 0.25 mg ml^−1^ Proteinase K, 900 μl of 0.45 M EDTA (0.5 M, pH 8.0) and 75 μl of H_2_O and rotated for 14–16 hours at 37 °C. The extraction lysates were transferred into a new tube and mixed with 10 ml of binding buffer (GuHCl 5 M, isopropanol 40% and UV H_2_O) and 400 µl sodium acetate. The solution was spanned through into silica columns for high volumes (High Pure Viral Nucleic Acid Large Volume Kit; Roche) and purified using the wash buffer provided in the kit. The purified DNA was then eluted in 2 × 50 μl of Tris-EDTA-Tween (TE buffer and 0.05% Tween 20), and the DNA extracts were stored at −20 °C (ref. ^85^).

We produced double-stranded libraries treated with uracil-DNA glycosylase (UDG) using 25 μl of extract in 50 µl per reaction (UDG-half protocol)^86,87^. The libraries were indexed using a unique combination of two indexes that were incorporated into the library molecules as a sample-specific DNA barcode^88^. The indexed libraries of each sample were then amplified using different PCR cycles to reach 1.5 × 10^13^ copies. The amplified products were then purified using MinElute spin columns following the manufacturer’s protocol and quantified on the Agilent 4200 TapeStation System. The quantified indexed libraries were pooled equimolarly to reach 10 nM, and shallow shotgun sequencing was performed on Illumina NextSeq500 or HiSeq 4000 instruments.

### Sample selection for SNP targeted enrichment

The shallow shotgun sequencing data were used to estimate the preservation of ancient DNA extracted from the archaeological skeletal remains. A percentage of endogenous human DNA above 0.1% and DNA damage at the molecule termini of above 5% were used as authenticity criteria, estimated using the software EAGER v.1.92.55 (ref. ^89^). Sequencing quality filtering (min. 20), length filtering (min. 30 bp) and adapter clipping (min. 1 bp) were performed with AdapterRemoval v.2 (ref. ^90^). The resulting reads were mapped against the human genome reference hg19 with the Burrows–Wheeler aligner^91^, duplicated reads were masked using MarkDuplicates (Picard) and damage patterns were calculated with mapDamage2.0 (ref. ^92^).

After shallow shotgun screening, libraries with values above the previously described thresholds were re-amplified and captured for ~1.24 million SNPs across the human genome (1240k SNP capture) and the entire mtDNA^67^. The enriched libraries were sequenced on Illumina NextSeq500 or HiSeq 4000 instruments. After sequencing, the capture data were demultiplexed using bcl2fastq v.2.17.1.14 (Illumina conversion software) and dnaClust v.3.0.0 (ref. ^93^).

### Ancient DNA authentication and genome-wide data processing

A total of 49 individuals were enriched for the 1240k SNPs. The captured individuals were aligned against the human reference genome hg19 using the Burrows–Wheeler aligner^91^. Damage pattern, coverage depth and DNA capture efficiency were estimated using published tools integrated within the EAGER pipeline^89^. We measured the level of X-chromosome contamination using ANGSD^68^ for male individuals and mtDNA contamination using schmutzi^94^ for all individuals (Supplementary Data 1). We excluded 11 individuals showing more than 4% human DNA contamination for at least one of the performed tests.

Genotype calls were performed using pileupCaller^95^ (v.1.4.0.2). We trimmed three base pairs at both ends of the reads for the double-stranded UDG-half libraries. After independent calls on the untrimmed and trimmed sets, we combined the genotype calls, selecting transitions from the trimmed genotype files and transversions from the untrimmed ones. Since the published individuals from Lapa do Sumidouro were processed using a library protocol without UDG treatment^65^, we processed the bam files separately, calling only transversions from the untrimmed data. We excluded 4 individuals with less than 40,000 SNPs overlapping the 1240k panel for a total of 34 individuals with newly generated genome-wide data usable for further analyses.

A PCA was generated with present-day worldwide individuals to calculate the genetic variation onto which ancient samples were projected using smartpca^96^ on the 1240k dataset. All ancient individuals from Brazil fall in a cluster with present-day Native Americans, which also includes the ancient Central and South American individuals published in Posth et al.^63^ (Extended Data Fig. 1). A clustering analysis was performed with present-day worldwide populations and ancient South American individuals genotyped for the Human Origins dataset^69^ using ADMIXTURE^97^ in unsupervised mode (Extended Data Fig. 2).

### *f* statistics

We created three datasets for genome-wide analyses combining the newly and previously generated data from ancient Brazilian individuals^62,63^ with (1) 1240k Allen Ancient DNA Resource v.32.7, (2) the Illumina panel^72^ and (3) the Human Origins panel^54,65,69,73,98^ (Supplementary Data 8). All *f* statistics were performed using the Mbuti population from Africa with diploid genotypes (.DG) as the outgroup.

To assess genetic affinities among ancient groups and between ancient groups and present-day indigenous populations from Brazil, we measured shared genetic drift using *f*_3_ outgroup statistics (inbreed, YES)^69^.

We computed *f*_4_ statistics (f4mode, yes)^69^ in the forms *f*_4_(Mbuti.DG, X; Ancient Brazilian A, Ancient Brazilian B) and *f*_4_(Mbuti.DG, X; present-day Brazilian group A, present-day Brazilian group B), where X represents the tested present-day or ancient Brazilian individuals/groups. The 1240k dataset was used to investigate the affinities among ancient Brazilian individuals/groups. The Illumina and Human Origins panels were used to describe the genetic affinities with present-day groups (Fig. 3). To minimize the impact on the analysis of ancestry introduced post-contact into the Americas, we used the masked version of the Illumina dataset^72^, while for the Human Origins dataset, we selected individuals carrying only Native American ancestry on the basis of PCA and ADMIXTURE analyses (Extended Data Figs. 1 and 2).

To investigate the proportion of Anzick-1-related ancestry (alpha) in the ancient South American genomes, we calculated *f*_4_-ratio statistics using qpF4Ratio (ref. ^70^) with the following formula:

$$\begin{array}{ll}f_4\;{\mathrm{ratio}}=1\;-\frac{f_4({{\mathrm{Mbuti}}}.{{\mathrm{DG}}},\,{{\mathrm{Anzick}}}.{{\mathrm{SG}}};\,{{\mathrm{Lauricocha}}}.8600{{\mathrm{BP}}},\,{{\mathrm{test}}})}{f_4({{\mathrm{Mbuti}}}.{{\mathrm{DG}}},\,{{\mathrm{Anzick}}}.{{\mathrm{SG}}};\,{{\mathrm{Lauricocha}}}.8600{{\mathrm{BP}}},\,{{\mathrm{Los}}}\,{{\mathrm{Rieles}}}.11900{{\mathrm{BP}}})}\end{array}$$

### qpWave analysis

We also tested the minimum number of streams of ancestry necessary to explain the genetic variation observed in the South American ancient genome-wide data. The tests were computed using qpWave software^69^ with the following settings: allSNPs, YES; significance threshold, ‘taildiff’ < 0.01. The left population was a combination of different pairs of ancient individuals/groups. The first set of right populations was previously used in Posth et al.^63^ and consisted of Mbuti.DG, Onge.DG, French.DG, Han.DG, Russia_MA1.SG and USA_Anzick.SG. Furthermore, we included additional shotgun data (Chile_Ayayema_5100BP.SG, E_San_Nicolas.SG, Mainland_Chumash.SG, San_Francisco_May.SG, LSCI.SG, SanClemente-SantaCatalina_800BP, Chipewyan.DG and Russia_Karelia_HG.SG) and present-day Mexican groups (Zapotec and Mixe). To identify an informative set of right populations, we prepared an array of comparisons using different combinations. We started with a set of outgroups composed of non-Native Americans and Anzick-1 (outgroup 1) and progressively added one individual or group at the time to this growing list, estimating each time the minimum number of streams of ancestry. The most informative combination of right populations to distinguish the genetic ancestry of the analysed ancient individuals/groups is presented in Supplementary Data 3.

### Uniparental markers and genetic diversity

To gain an overview of the mtDNA diversity of ancient individuals from Brazil, we produced mtDNA capture data for each sample, and we assigned mtDNA haplogroups using Haplogrep 2.0 and Haplofind^99,100^. To reconstruct the mtDNA consensus sequences, we applied four quality thresholds (q0, q10, q20 and q30) to the likelihood estimated for each position by schmutzi. We used the YhaploCaller^101^ to assign Y-chromosome haplogroups followed by manual checking to verify the called SNPs for each male individual.

The pairwise *F*_ST_ presented in Extended Data Fig. 6 was performed using the mtDNA aligned using MUSCLE v.3.8 (ref. ^102^) and manually inspected/edited. The mtDNA indels and mutational hotspots under the nucleotide positions 309.1C(C), 315.1C, AC indels at 515–522, 16182C, 16183C, 16193.1C(C) and C16519T (ref. ^103^) were removed from the alignment.

Heterozygosity was estimated using pileupCaller^95^ to produce pseudo-diploid genotype calls and calculated using the ratio between the number of sites in heterozygosity and the total number of covered sites, multiplied by two. The individual values were grouped in three broad regions/temporal intervals. To investigate the magnitude of the differences in the heterozygosity levels, we performed the non-parametric Kruskal–Wallis test. Conover’s post-hoc analysis was performed to determine the differences between groups using a correction for multiple comparisons (R v.3.6.0 tidyverse^104^ and conover.test^105^ packages) (Fig. 5b).

### ROH and kinship analysis

We used hapROH with the default parameters^80^ to estimate the length of segments in homozygosity for individuals with coverage higher than 190,000 SNPs (Fig. 5a). To investigate the degree of genetic relatedness between the ancient individuals, we applied READ^106^ and calculated the pairwise mismatch rate^107^ (Supplementary Data 9 and Extended Data Fig. 7). For population genetic analyses of the Jabuticabeira II_~2400BP group, we excluded the first-degree relationships, retaining in such pairs the individuals with the highest SNP coverage.

### Direct radiocarbon dating

We produced new radiocarbon dates for 23 individuals among the 34 with usable ancient genomic data analysed in this study. The direct dates of the other seven individuals were obtained from previous studies, while four individuals were not directly dated (Supplementary Data 1). In addition, we produced new radiocarbon dates for eight individuals without sufficient ancient DNA quality for population genomic analyses (Supplementary Information and Supplementary Data 1). A rib fragment from the Capelinha individual ‘Luzio’ was pretreated at the Department of Human Evolution, Max Planck Institute for Evolutionary Anthropology, Leipzig, Germany, using the method described in refs. ^108,109^ and the resulting collagen was sent for dating to the Curt-Engelhorn-Zentrum Archäometrie gGmbH in Mannheim, Germany. Instead, bone fragments from the other dated individuals were directly sent to the Mannheim dating lab. Collagen was extracted and purified by ultrafiltration (fraction, >30 kDa), freeze-dried and combusted to CO_2_ in an elemental analyser. The CO_2_ was catalytically converted to graphite, and the dating was performed using a MICADAS-AMS machine. The resulting ^14^C ages were normalized to d^13^C = −25‰ and calibrated using OxCal v.4.4 software^110^ with the SHCal20 curve^111^ (Supplementary Data 1 and 10). The calibrated dates were not corrected for marine radiocarbon reservoir effect, which could influence age estimations for individuals with strong marine diets.

### Strontium isotope analysis

Strontium isotopic analysis (^87^Sr/^86^Sr) of skeletal material is commonly used to detect geographic provenance and mobility among mammals, including humans^112,113^. The tooth enamel records the isotopic signal of when it was formed during the earliest stages of life, whereas the bone isotopic signal reflects a period closer to the time of death of the individual^114^. Since the radiogenic isotope ^87^Sr forms by radioactive decay from rubidium (^87^Rb), the ^87^Sr/^86^Sr signature of a specific location is determined by the underlying bedrock age and its content of Rb^115^. A specific geological strontium signature is incorporated into the hard body tissues by direct substitution for calcium^116^ since strontium enters the ecosystem without fractionation^117^.

We measured the ^87^Sr/^86^Sr ratios from enamel samples of ten individuals from the Jabuticabeira II site (Extended Data Fig. 9 and Supplementary Table 16). Sample preparation and analysis were done in dedicated isotope facilities at the University of Cape Town (South Africa), as described below. Prior to analysis, an enamel sample was taken from along the longitudinal axis of the crown, thus representing a single average value for the years while the crown was developing. This portion of enamel (ca. 20 mg) was cleaned by abrasion and possible dentine remains were removed using a Dremel 3500 drill bit, rinsed and ultrasonicated for 20 minutes in MilliQ water. Diamond drill bits were cleaned with ethanol and ultrasonicated in MilliQ water between samples to avoid cross-contamination^118^. After this, the cleaned enamel sample was digested with 2 mL bi-distilled distilled 65% HNO3 in a closed Teflon beaker placed on a hotplate at 140 °C for an hour. Digested samples were then dried and redissolved in 1.5 mL of bi-distilled 2M HNO3. These redissolved samples were centrifuged at 4000 rpm for 20 minutes, and the supernatant was collected for strontium separation chemistry. A separate fraction for each sample in this step was used to calculate the concentration with 88Sr intensity (V) regression equation built with SRM987 standard from NIST (National Institute of Standards and Technology, Gaithersburg, MD, USA). Strontium was isolated with 200μl of Eichrom Sr.Spec resin loaded in Bio-Spin Disposable Chromatography Bio-Rad Columns following the method of (ref. ^119^). The separated strontium fraction for each sample was dried down, dissolved in 2 ml 0.2% bi-distilled HNO3 and diluted to 200 ppb Sr concentrations for isotope analysis. 87Sr/86Sr ratios were measured using a NuPlasma HR multicollector inductively-coupled-plasma mass spectrometer (MC-ICP-MS). Sample analyses were referenced to bracketing analyses of SRM987, using a 87Sr/86Sr reference value of 0.710255 from NIST. All strontium isotope data are corrected for isobaric rubidium interference at 87 amu using the measured signal for 85Rb and the natural 85Rb/87Rb ratio. Instrumental mass fractionation was corrected using the measured 86Sr/88Sr ratio and the exponential law, and a true 86Sr/88Sr value of 0.1194 (ref. ^120^). Results for repeat analyses of an in-house carbonate standard (87Sr/86Sr = 0.708936; 2*σ* 0.000041; *n* = 33) and an in-house ocean island basalt standard (87Sr/86Sr = 0.704888; 2*σ* 0.000027; *n* = 33) processed and measured with the batches of samples in this study are in agreement with long-term results for these two in-house standards (87Sr/86Sr; 0.708915; 2*σ* 0.000047; *n* = 125) (87Sr/86Sr; 0.704902; 2*σ* 0.000035; *n* = 67). For every two batches one blank was added to assess the cleanness of the process; there was no peak and, thus, no contamination from external Sr in any of the batches. The ^87^Sr/^86^Sr values from the Jabuticabeira II individuals have an average of 0.710 ± 0.006 (1*σ*), with minimum and maximum values of 0.709 and 0.711. These values are within the range of sea water and within the range previously established for other shellmound individuals from the southern coast of Brazil^44,121^, indicating that all analysed individuals grew up in a coastal environment. Nevertheless, the average ^87^Sr/^86^Sr ratios differ for the three genetic groups identified at Jabuticabeira II. The ^87^Sr/^86^Sr ratio increases over time with averages of 0.7095 ± 0.000096 (*n* = 7) for the JabuticabeiraII_~2400BP group, 0.7104 ± 0.00025 (*n* = 2) for JabuticabeiraII_111/112_~2200BP and 0.7111 (*n* = 1) for JabuticabeiraII_1300BP.

### Terminology used to describe ancient individuals and groups

The terminology used here to classify ancient Brazilian societies does not represent the entire diversity of indigenous peoples in the country, nor should it be understood as reflecting a shared identity. The archaeological information indicates a complex demographic history (see Supplementary Information for a detailed description of each archaeological site analysed in this study). The complexity and contextual diversity of pre-Columbian indigenous peoples prevents a classification system that could successfully capture the genetic diversity in Brazil during the Holocene. To connect the archaeological assemblages with the genetic information, we used a combination of the following classifications: foraging strategy (hunter-gatherers, fisher-hunter-gatherers and horticulturists), time scale (early Holocene, ~10,000–7,000 yr bp; middle Holocene, ~7,000–4,000 yr bp; and late Holocene, ~4,000–0 yr bp), cultural assemblages (riverine sambaquis, coastal sambaquis and the ceramic traditions Taquara-Itarare, Una, Koriabo and Tupiguarani) and geographical regions (southern and southeastern Atlantic coast, Lagoa Santa region, central Brazil, northeastern Brazil and lower Amazon).

The cultural assemblages are part of a diverse record of pre-Columbian material culture and help contextualize the settlement of the southern and southeastern Brazilian coast. The term ‘tradition’ is applied in Brazilian archaeology to refer to common technological and stylistic traits in the production of ceramics with chronological persistence in the archaeological record. The ceramic traditions from the late Holocene are directly associated with present-day ethnolinguistic groups and represent a putative connection between ancient individuals and present-day indigenous peoples. However, the present-day populations in our dataset represent only a small fraction of the diversity of indigenous peoples currently living in Brazil.

### Reporting summary

Further information on research design is available in the Nature Portfolio Reporting Summary linked to this article.

## Supplementary information

Supplementary Information Supplementary Discussion, Figs. 1–15 and Tables 1–40.

Reporting Summary

Supplementary Data 1 Archaeological information, radiocarbon dating and ancient DNA summary statistics.

Supplementary Data 2 *f*_4_ and *f*_3_ outgroup statistics 1240k dataset.

Supplementary Data 3 qpWave analysis.

Supplementary Data 4 Anzick-1-related affinity.

Supplementary Data 5 *f*_4_ statistics Human Origins and Illumina datasets.

Supplementary Data 6 Zoro-related affinity.

Supplementary Data 7 Population Y tests.

Supplementary Data 8 1240k and Human Origins comparative datasets.

Supplementary Data 9 Pairwise mismatch rate results.

Supplementary Data 10 Details radiocarbon dating.

## Data Availability

The alignment files of the nuclear DNA and mtDNA sequences for the newly reported individuals are available at the European Nucleotide Archive under the accession number PRJEB51863.
