# Supplementary material for: Genomic history of coastal societies from eastern South America
Source: Nat Ecol Evol. 2023 Jul 31;7(8):1315–30. doi: 10.1038/s41559-023-02114-9 (PMC10406606; doi:10.1038/s41559-023-02114-9)
Supplement: Supplementary file 1 — Supplementary Discussion, Figs. 1–15 and Tables 1–40. [file 41559_2023_2114_MOESM1_ESM.pdf]

nature ecology & evolution

Article https://doi.org/10.1038/s41559-023-02114-9

Genomic history of coastal societies from eastern South America

In the format provided by the authors and unedited

Archaeological sites with preserved ancient DNA

Sambaqui Cabeçuda

Analyzed Samples

Table S1: Samples from Cabeçuda analyzed for aDNA in this study.

IPHAN ID	MPI ID	Context	Bone part	DNA
CABE-01	CBE001	Burial 11	pars petrosa	No
CABE-02	CBE002	Burial 12	pars petrosa	No
CABE-03	CBE003	Burial 16	pars petrosa	Yes
CABE-04	CBE004	Burial 15	pars petrosa	Yes

Chronology

Table S2: Published radiocarbon dates on human skeletons from Cabeçuda.

IPHAN ID	MPI ID	LAB ID[1]	Context	^{14}C age [BP]	Source
n/a[*]	n/a	383565	Burial 5	2990 ± 30	Kneip et al. 2018
CABE-04	CBE004	383566	Burial 15	3030 ± 30	Kneip et al. 2018
n/a[*]	n/a	383567	Burial 20	2920 ± 30	Kneip et al. 2018
n/a[*]	n/a	297831	MN, nº1682[**]	2030 ± 30	Scheel-Ybert et al. 2020
n/a[*]	n/a	237832	MN, nº1750[**]	1990 ± 30	Scheel-Ybert et al. 2020
n/a[*]	n/a	297833	MN, nº1749[**]	1800 ± 30	Scheel-Ybert et al. 2020
n/a[*]	n/a	297834	MN, nº1798[**]	2290 ± 30	Scheel-Ybert et al. 2020
n/a[*]	n/a	280009	Burial 6 (*Locus 1*)	3870 ± 40	Scheel-Ybert et al. 2020

[1]All dates done at Beta Analytics. (*) Samples without IPHAN-ID were not processed for aDNA as part of this study. (**) Samples obtained from human burials excavated by Luis Castro Faria during the 1920s and housed at the National Museum - Federal University of Rio de Janeiro (NM). (n/a) Not available.

Table S3: New radiocarbon date for a human skeleton from Cabeçuda, this study. Calibrated using Oxcal 4.4 (Bronk Ramsey 2021) - SHCal20 (Hogg et al. 2020).

IPHAN ID	MPI ID	LAB ID[1]	Context	^{14}C age [BP]	cal BP	C [%]	C:N	Collagen [%]
CABE-03	CBE003	43997	Burial 16	3352 ± 27	3685-3487	19.5	3.5	0.2

[1]Curt-Engelhorn-Centre for Archaeometry, Mannheim. Sample 43997 has a very low collagen yield (below 0,5%). Although these types of samples are not suitable for dating, the laboratory reported a C:N ratio that is at normal rate and proceeded with the dating. However, the low collagen yield makes this sample more prone to contamination and degradation, affecting the ^{14}C age.

Table S4: Published radiocarbon dates on non-skeletal material from Cabeçuda.

LAB ID	Context	^{14}C age [BP]	Material	Source
280005[1]	2010's excavation, 25-30cm	3640 ± 50	Shell (*Anomalocardia flexuosa*)	Kneip et al. 2018
280006[1]	Locus 1, northwest wall	4180 ± 60	Shell (*Anomalocardia flexuosa*)	Kneip et al. 2018
280007[1]	Locus 3, southwall, top	4020 ± 50	Shell (*Anomalocardia flexuosa*)	Kneip et al. 2018
280008[1]	-	4180 ± 60	Shell (*Anomalocardia flexuosa*)	Kneip et al. 2018
Hannover167	-	4120 ± 220	Charcoal	Mello Alvim et al. 1984

[1] Samples dated at Beta Analytics.

Archaeological context

Cabeçuda is a shellmound (ca. 4200-1800 non-cal BP) located at the municipality of Laguna in the state of Santa Catarina, Southern coast of Brazil. This site was once amongst the largest shellmounds of the world with a diameter of ca. 100 meters, ca. 25 meters in height and an estimated volume of 53,000 m^3 [3,5,7–9]. However, since the 19th century construction work has severely impacted the archaeological site leaving a mound with no more than 4 meters in height [10]. Between 1950 and 1951 Luis Castro Faria, an archaeologist from the National Museum at Rio Janeiro, excavated 140 square meters of the site reaching up to 8.5 meters deep in the stratigraphy and exhuming 191 human skeletons – Figure S1 [4,6,7,9,11–14]. These skeletons were not sampled for the present study.

Is the last decade new excavations were conducted at Cabeçuda by a series of research projects coordinated by Dr. Paulo de Blasis from the Museum of Archaeology and Ethnology of the University of São Paulo, and Dr. Maria Dulce Gaspar, Dr. Rita Scheel-Ybert and Dr. Claudia Rodrigues-Carvalho from the National Museum of Rio de Janeiro [3]. In 2012, the duplication of a national highway resulted in further excavations of the site by GRUPEP/Unisul, under the coordination of Dr. Deisi Scunderlick de Farias [2], including the excavation of eight human skeletons, some of which were sampled for the present study.

Formation process analysis suggests that Cabeçuda was mainly used to bury the dead with small shellmounds being deposited on top of individual graves [15]. The archaeological matrix is composed of

sandy layers with non-fragmented bivalve shells (mainly *Anomalocardia flexuosa*). The presence of fish bones, charcoal, and lithic material is also common [1–3,6,16].

The ubiquitous presence of human skeletons through the mound's excavations and stratigraphic profiles suggests that dozens of thousands of interments took place in the locality [17]. However, in spite of the demographic success implied by the large number of skeletons, the analysis of osteological markers points to impaired developmental conditions. Analysis of 155 crania from Cabeçuda indicates a high incidence of *cribra orbitalia,* particularly among infants. It is not likely that populations inhabiting a coastal environment were nutritionally deprived, the systemic anemia probably developed from severe parasite mediated infections [18,19]. While reported for other shellmounds from Santa Catarina in Southern Brazil, treponematosis was not detected among individuals from Cabeçuda [20]. Enamel hypoplasia, Harris lines, and porotic hyperostosis also support a scenario of developmental stress among this population [7,21]. On the other hand, analysis of trauma suggests that in spite of the large demographic density, interpersonal violence was not common [22]. The individuals from Cabeçuda are unique in presenting virtually no caries lesions[23] and a pattern of tooth loss concentrated in the lower incisors that was proposed to reflect the use of labrets [24]. Craniometric studies have shown that individuals from Cabeçuda are morphologically homogeneous and, in a regional perspective, are strongly associated with other shellmound populations from Southern Brazil [14,25,26].

The chronology of the site is based on four radiocarbon dates on shells – ranging from 4180 ± 60 to 3640 ± 50 BP – and eight radiocarbon dates on human bone – ranging from 3870 ± 40 to 1800 ± 30 BP. Both individuals analyzed in the present study were directly dated: Burial 15 (3030 ± 30 BP [5]) and Burial 16 (3352 ± 27 BP, this study).

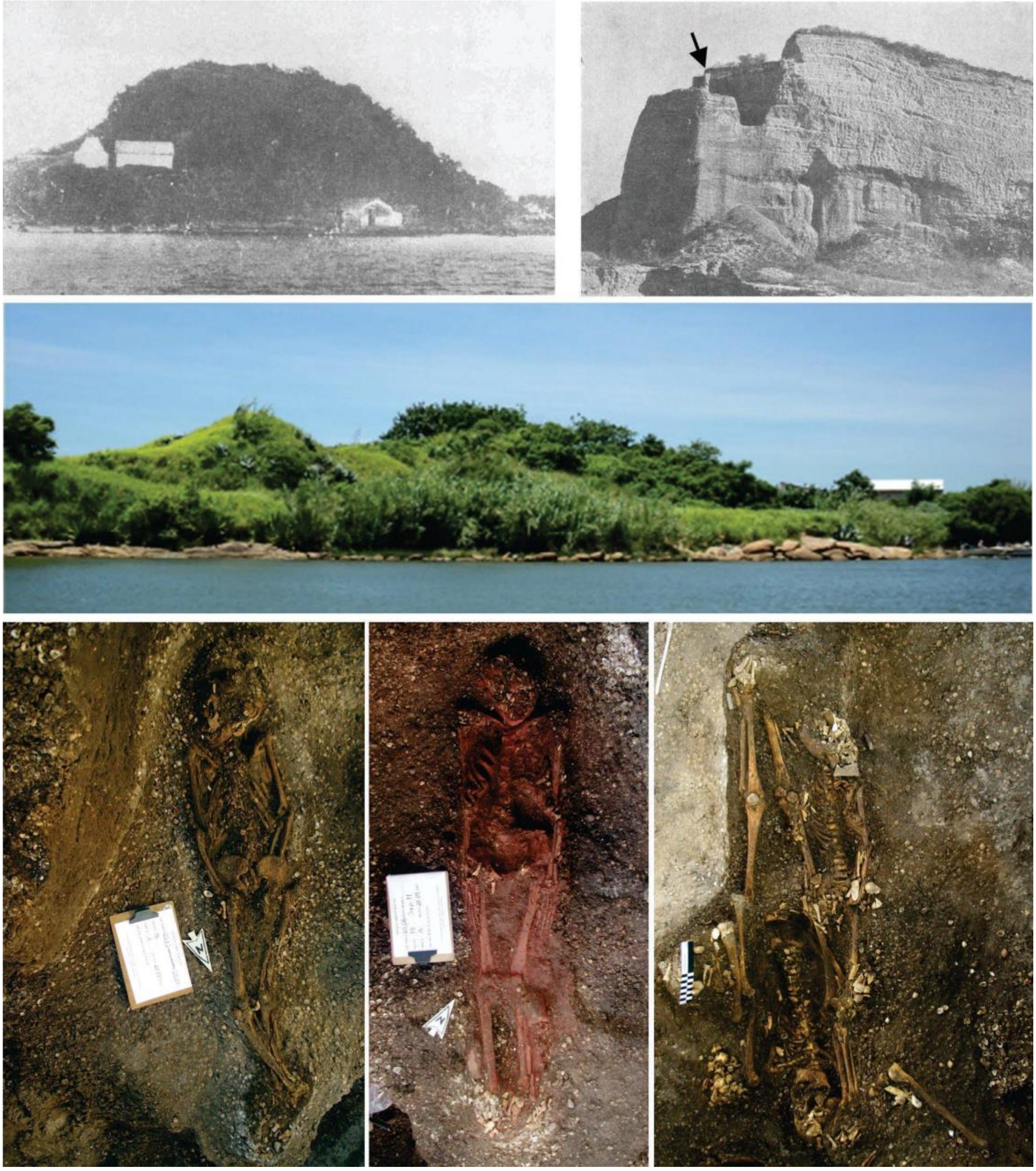

Figure S1: Sambaqui Cabeçuda. A) Cabeçuda was a massive shellmound estimated to be 40 meters height before degradation started (Photo taken in 1929, reproduced from Luis Castro Faria); B) Construction work exposed the interior of the shellmound, black arrow points to a man for scale (Photo taken in 1950, reproduced from 1959); C) Today not much is left from Cabeçuda, besides a mound on the side of a federal highway; D-F Field photographs of human skeletons during recent excavations.

Fishmound Galheta IV

Analyzed Samples

Table S5: Samples from Galheta IV analyzed for aDNA in this study.

IPHAN ID	MPI ID	Context	Bone part	DNA
GALHE4-01	GA4001	Burial 01	pars petrosa	No
GALHE4-03	GA4002	Burial 03b	pars petrosa	Yes
GALHE4-05	GA4003	Burial 04	pars petrosa	No
GALHE4-07	GA4004	Burial 05	pars petrosa	No
GALHE4-09	GA4005	Burial 07	pars petrosa	No

Chronology

Table S6: Published radiocarbon dates on human skeletons from Galheta IV.

IPHAN ID	LAB ID	Context	^{14}C age [BP]	Source
n/a	Beta 211734	Burial 1	980 ± 40	DeBlasis et al. 2014
n/a	Beta 280010	Burial 3	1360 ± 40	DeBlasis et al. 2014
n/a	UGAMS 30089	Burial 4	830 ± 43	Cardoso 2019
n/a	UGAMS 30090	Burial 6	990 ± 44	Cardoso 2019
n/a	Beta 280012	Burial 7	950 ± 40	DeBlasis et al. 2014
n/a	Beta 280011	Unit 112/93, level 3*	1070 ± 40	DeBlasis et al. 2014

(n/a) Not available. *Pinniped bone

Table S7: New radiocarbon dates for human skeletons from Galheta IV, this study. Calibrated using Oxcal 4.4 (Bronk Ramsey 2021) - SHCal20 (Hogg et al. 2020).

IPHAN ID	MPI ID	LAB ID[1]	Context	^{14}C age [BP]	cal BP	C [%]	C:N	Collagen [%]
GALHE4-05	GA4003	40645	Burial 4	899 ± 17	793-728	40.2	3.0	4.3
GALHE4-07	GA4004	40646	Burial 5	1105 ± 17	1046-926	41.8	3.2	6.9

Archaeological context

Galheta IV is a small fishmound on the Southern coast of Brazil, occupied from 1360 ± 40 to 830 ± 43 BP, and composed of a single archaeological layer made of organic-rich black sediments with fish bones and no shells [27,28]. The site is located in a 50 meters height granitic promontory (Pontão da Galheta) on top of which two shellmounds are also located. Galheta I is dated to 3090 ± 70 BP and is a large mound ca. 20 meters tall and a diameter of ca. 250 meters. Galheta II is dated from 4530 ± 70 to 4400 ± 60 and is a small shellmound with ca. 3 meters in height and a diameter of ca. 20 meters [29,30].

Between 2005 and 2007 an area of 33 m^2 was excavated by a team coordinated by Dr. Paulo DeBlasis from the Museum of Archaeology and Ethnology of the University of São Paulo and Dr. Deisi Farias from GRUPEP/Unisul[2,27] – Figure S2. The presence of eight human burials indicates the primary use of the location as a mortuary site. Excavations also returned a large collection of polished and chipped stone artifacts.

Importantly, Galheta IV registered the appearance of pottery in this region of the Atlantic coast in the form of dark, thin-walled shreds that sometimes include plastic decoration in the form of small pits. This type of ceramics is attributable to the Taquara-Itararé Tradition [27], which is associated with the southern proto-Jê populations that inhabited the highlands of Southern Brazil from approximately 3000 years BP [31]. The time of appearance of the Taquara-Itararé pottery on the coast coincides with the ceasing of shellmound construction around 2000 years ago. This drastic transformation of the archaeological record could reflect a process of population substitution (complete or partial – with or without admixture) or the initiation/intensification of cultural contacts – symmetric or not - between inland and coastal populations [27, 28].

The inhabitants of Galheta IV, although using pottery that is typically found in the highlands associated with processing of vegetable foods, had a mostly maritime diet. Faunal remains from the site included pelicans, seals, sharks, turtles, cetaceans and pelagic fish [29,32]. Isotopic analysis characterizes a diet mainly focused on maritime resources [33]. Analysis of biomarkers from the ceramics found in Galheta IV shows that they were not used to cook vegetables but instead marine animals, likely fish [33].

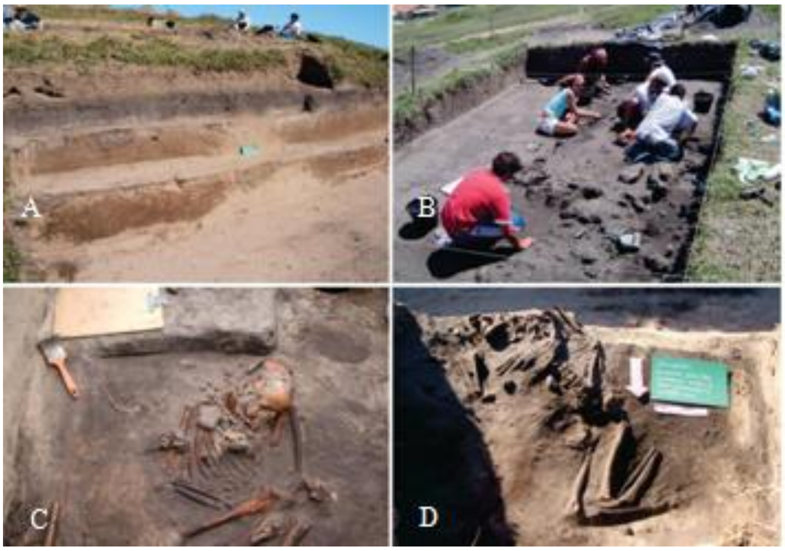

Figure S2: Sambaqui Galheta IV. A - B) Panoramic view of the site during archaeological excavation; C - D) Human burials found at the site.

Sambaqui Cubatão I

Analyzed Samples

Table S8: Samples from Cubatão I analyzed for aDNA in this study.

IPHAN ID	MPI ID	Context	Bone part	DNA
CUBA – 1	CUB002	Burial 01A	Tooth	Yes
CUBA – 2	CUB003	Burial 01B	Tooth	No
CUBA – 4	CUB005	Burial 5	Tooth	No
CUBA – 6	CUB001	Burial 7	Tooth	No
CUBA – 8	CUB008	Burial 11	Tooth	Yes

Table S9: Published radiocarbon dates on human skeletons from Cubatão I.

IPHAN ID	LAB ID	Context	^{14}C age [BP]	Source
n/a	Beta 259823	Upper sector, Burial 1a	2430 ± 40	Fossile et al. 2019
n/a	Beta 268523	Upper sector, Burial 11	2460 ± 40	Fossile et al. 2019
n/a	Beta 268525	Upper sector, Burial 14	2460 ± 40	Fossile et al. 2019
n/a	Ly 4524	Upper sector, Burial 1b	2460 ± 30	Fossile et al. 2019
n/a	Ly 4527	Upper sector, Burial 6	2495 ± 30	Fossile et al. 2019
n/a	Beta 268524	Upper sector, Burial 12	2510 ± 40	Fossile et al. 2019
n/a	Beta 259519	Upper sector, Burial 4	2520 ± 40	Fossile et al. 2019
n/a	Ly 4528	Upper sector, Burial 7	2520 ± 40	Fossile et al. 2019
n/a	Ly 4526	Upper sector, Burial 5	2620 ± 30	Fossile et al. 2019
n/a	Beta 259821	Upper sector, Burial 9	2630 ± 40	Fossile et al. 2019
n/a	Beta 259820	Upper sector, Burial 8	2670 ± 40	Fossile et al. 2019

(n/a) Not available.

Table S10: New radiocarbon dates for human skeletons from Cubatão I, this study. Calibrated using Oxcal 4.4 (Bronk Ramsey 2021) - SHCal20 (Hogg et al. 2020).

IPHAN ID	MPI ID	LAB ID[1]	Contexto	^{14}C age [BP]	cal BP	C [%]	C:N	Collagen [%]
CUBA-8	CUB008	38996	Burial 11	2602 ± 21	2756-2725	39.9	2.9	6.2
CUBA-1	CUB002	45781	Burial 1A	2492 ± 22	2721-2490	43.4	3.2	7.4

[1] Curt-Engelhorn-Centre for Archaeometry, Mannheim.

Table S11: Published radiocarbon dates on non-skeletal material from Cubatão I.

LAB ID	Context	^{4}C age [BP]	Material	Source
Beta 259823	Upper sector, Grid N05E01	2560 ± 40	Shell (*Anomalocardia flexuosa*)	Fossile et al. 2019
Beta 268526	Upper sector, Grid N09W12	2250 ± 40	Charcoal	Fossile et al. 2019
Beta 259824	Upper sector, Grid N17E02	2660 ± 40	Shell (*Anomalocardia flexuosa*)	Fossile et al. 2019
Beta 259827	Lower sector, Survey S49E08	2890 ± 60	Charcoal	Fossile et al. 2019
Beta 259825	Lower sector, Grid N20W33	2970 ± 60	Charcoal	Fossile et al. 2019
Ly 4525	Lower sector, Center base	2975 ± 30	Charcoal	Fossile et al. 2019
Beta 259829	Lower sector, Survey	3040 ± 60	Charcoal	Fossile et al. 2019

	S55E20 F			
Beta 259826	Lower sector, Center base 1	3110 ± 70	Charcoal	Fossile et al. 2019
Beta 259828	Lower sector, Survey S55E20 C	3480 ± 60	Shell (*Anomalocardia flexuosa*)	Fossile et al. 2019

Archaeological context

Cubatão I is a shellmound located in the Southern coast of Brazil, near the city of Joinville, northern coast of Santa Catarina State. Built on the lagoon-estuarine system of Babitonga Bay, near the mouth of the Cubatão River, the elevated portion of the shellmound presents an oval shape and reaches approximately 8 meters in height, 80 meters in length on the north-south axis and 70 meters on the east-west axis. Its stratigraphy is complex with at least 20 major depositional layers, each one formed by many distinct lenses, and composed mainly of mollusk shells and fish bones, with birds and mammals bones in lower quantities [34,35].

From 2007 to 2009 an area of 44m^2 at the top of the mound was excavated by a team of archaeologists led by Dr. Levy Figuti from the Museum of Archaeology and Ethnology of the University of Sao Paulo, on a continuous surface and up to a maximum depth of 1.10 m. A series of exploratory pits (1m^2) were also opened at various locations in the base of the shellmound. Radiocarbon dates on charcoal fragments from the pits indicated that the construction of the mound began between 3110 ± 70 AP and 2975 ± 30 BP, and continued until the site stopped to be used at about 2250 ± 40 BP (charcoal from a hearth in the main excavation area) [34].

Zooarchaeological studies indicated that fish accounted for 99% of the faunal remains recovered, and that despite the predominance of marine catfish (*Siluriformes*) and puffer fish (*Tetraodontidae*), the builders of Cubatão I exploited at least 38 different fish taxa [36]. These findings are indicative of a diet based on marine resources, and are supported by stable isotope analysis, which indicated the inclusion of significant amounts of plants in diet [37]. The bottom layers are permanently drenched by the Cubatão River, which favored the preservation of baskets, ropes and cords, many showing a variety of knots. In addition to the artifacts made from *Philodendron corcovadensis* roots, wood stakes were also recovered made of at least six different arboreal species, some of which were tied to each other with cords forming a large and horizontal grid-like structure [38–41].

In the main excavation area, the skeletons of 22 individuals were found in 21 primary burials. All adult skeletons (6 males, 5 females and 1 undetermined sex) were directly radiocarbon dated. The chronology indicates that the funerary area was built between 2670 ± 40 BP and 2430 ± 40 BP, and is formed from three burial concentrations, two of them contemporary and another slightly older – Figure S3. The majority of the individuals were children under the age of 12 months at the time of death. Among the adults, there was no significant distinction in the treatment of the deceased: they were deposited on the surface hyperflexed and in lateral decubitus (a grave pit was identified in only one

case), hands over the face, elbows touching knees, and were later covered by mounds built with fauna remains, mainly mollusk shells. Red ochre was used for adults in different amounts and no other funerary goods were recovered. In contrast, children presented a greater variation of burial positions and a greater amount of ochre, as well as a distinctive feature: all children under one year-old had necklaces made of shell beads and bone pendants as funerary goods[34].

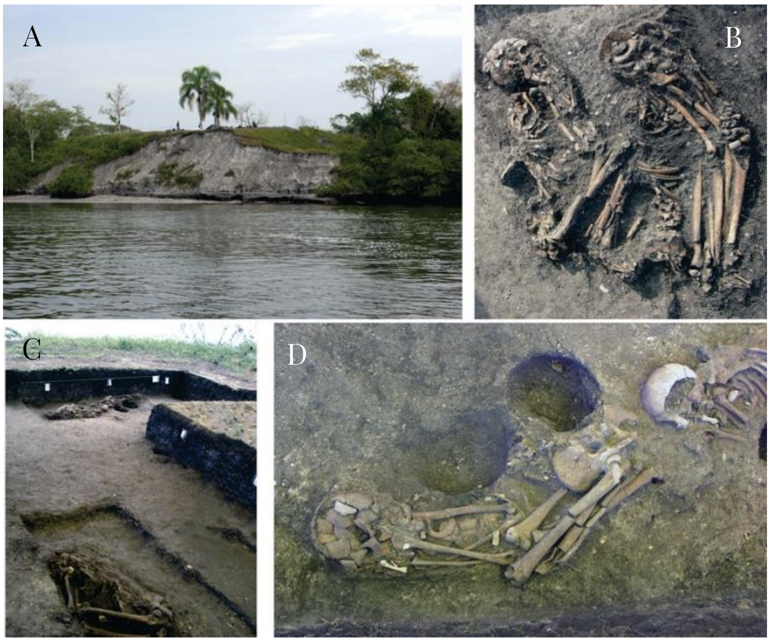

Figure S3: Sambaqui Cubatão I. A) View of the site at the margins of the Cubatão river, currently threatened by fluvial erosion; B - D) Photographs of some of the human burials found at the site.

Sambaqui Jabuticabeira II

Analyzed Samples

Table S12: Samples from Jabuticabeira II analyzed in this study.

IPHAN ID	MPI ID	Context	Bone part	DNA
JAB-18	JBT008	Burial 2A (L6-E3)	pars petrosa	No
JAB-19	JBT009	Burial 38 (L2.05)	pars petrosa	Yes
JAB-20	JBT010	Burial 12A (L1-25-E1)	pars petrosa	Yes
JAB-21	JBT011	Burial 12C (L1.25-E1)	pars petrosa	Yes

JAB-22	JBT012	Burial 43 (L1.77-E3)	pars petrosa	Yes
JAB-23	JBT013	Burial 110 (L2)	pars petrosa	Yes
JAB-24	JBT014	Burial 111/112A (Pathological individual TL1/L2)	pars petrosa	Yes
JAB-25	JBT015	Burial 111/112B (Intrusive Individual - TL1/L2)	pars petrosa	Yes
JAB-26	JBT016	Burial 114-L6	pars petrosa	No
JAB-27	JBT017	Burial 17A	pars petrosa	No
JAB-28	JBT018	Burial 34 (L2.05 - E4)	pars petrosa	Yes
JAB-29	JBT019	Burial 102 (L1-75 E5)	pars petrosa	Yes
JAB-30	JBT020	Burial 15 (L1.05-E1)	pars petrosa	Yes
JAB-31	JBT001	Burial 10B (L1.25 - E1)	pars petrosa	Yes
JAB-32	JBT021	Burial 37 (L2.05 - E4)	pars petrosa	Yes
JAB-33	JBT022	Burial 12B (L1.25 - E1)	pars petrosa	Yes
JAB-34	JBT023	Burial 25 (L2.65 E3)	pars petrosa	Yes
JAB-35	JBT002	Burial 11 (L1.25 - E1)	pars petrosa	Yes
JAB-36	JBT024	Burial 115B (L6)	pars petrosa	No
JAB-37	JBT025	Burial 17A (L2.05 - E3)	pars petrosa	Yes
JAB-38	JBT026	Burial 41A (L2.05)	pars petrosa	Yes

Table S13: Published radiocarbon dates on human skeletal from Jabuticabeira II.

IPHAN ID	MPI ID	LAB ID	Context	^{14}C age [BP]	Source
n/a	-	Beta 188382	Lócus 2.15.13 -, Burial 38	2320 ± 50	DeBlasis et al. 2007
n/a	-	Beta 188381	Lócus 2.15.13 -, Burial 26	2340 ± 50	DeBlasis et al. 2007
n/a	-	Beta 234201	Lócus 3, Burial 131	1400 ± 40	Kneip et al. 2018
n/a	-	Az AA77106	Locus 6, burial 2A	2028 ± 44	Kneip et al. 2018
n/a	-	Az AA77105	Locus 6, burial 114	2004 ± 44	Kneip et al. 2018
n/a	MPI 19	MAMS 28362	Lócus 1.77-E3, Burial 43	2348 ± 23	Posth et al. 2018
n/a	MPI 24	MAMS 28358	Lócus 2, Burial 110	2425 ± 23	Posth et al. 2018
n/a	MPI 22	MAMS 28363	TL1/L2, Burial 111/112A (pathological)	2145 ± 23	Posth et al. 2018
n/a	MPI 21	MAMS 28365	TL1/L2, Burial 111/112 (intrusive individual)	2203 ± 24	Posth et al. 2018
n/a	MPI 26	MAMS 28360	Lócus 2.05 - E4, Burial 34	2386 ± 23	Posth et al. 2018
n/a	MPI 23	MAMS 28359	Lócus 1.75 - E4, Burial 102	1498 ± 23	Posth et al. 2018
n/a	MPI 25	MAMS 28361	Lócus 1.25 - E1, Burial 10B	2482 ± 23	Posth et al. 2018
n/a	MPI 27	MAMS 28364	Lócus 1.25 - E1, Burial 11	2436 ± 23	Posth et al. 2018

(n/a) Not available

Table S14: New radiocarbon dates for human skeletons from Jabuticabeira II, this study. Calibrated using Oxcal 4.4 (Bronk Ramsey 2021) - SHCal20 (Hogg et al. 2020).

IPHAN ID	MPI ID	LAB ID[1]	Context	^{14}C age [BP]	cal BP	C [%]	C:N	Collagen [%]
JAB-38	JBT026	40643	Burial 41A	2310 ± 19	2355-2214	38.9	2.9	3.4
JAB-32	JBT021	40640	Burial 37	2323 ± 19	2357-2329	44.1	3.0	1.8
JAB-30	JBT020	40644	Burial 15	2331 ± 19	2359-2332	33.6	2.9	1.3
JAB-31	JBT001	38997	Burial 10B	2401 ± 21	2607-2351	34.1	2.6	3.6
JAB-37	JBT025	40642	Burial 17A	2407 ± 19	2491-2353	44.1	3.0	2.9
JAB-35	JBT002	38998	Burial 11	2411 ± 20	2667-2353	39.7	2.9	4.5
JAB-34	JBT023	40641	Burial 110	2420 ± 19	2675-2355	43.1	3.0	1.9
JAB-20	JBT010	40639	Burial 12A	2440 ± 20	2697-2360	39.2	2.9	3.6

[1] Curt-Engelhorn-Centre for Archaeometry, Mannheim.

Table S15: Published radiocarbon dates on non-skeletal material from Jabuticabeira II.

LAB ID	Context	^{14}C age [BP]	Material	Source
Az 09892	Locus 1, feature 1.15.6 (ou 1.05)	1895 ± 185	charcoal	DeBlasis et al. 2007
Beta 195250	Locus 1.75, perfil leste, camada 14	1950 ± 70	charcoal	DeBlasis et al. 2007
Beta 195249	Locus 1.75, perfil leste, camada 4	1970 ± 40	charcoal	DeBlasis et al. 2007
Az 09900	Locus 1.10, camada 3	1975 ± 95	charcoal	DeBlasis et al. 2007
Beta 195240	Locus 1.75, perfil leste, camada 16	2020 ± 40	charcoal	DeBlasis et al. 2007
Az 09897	Locus 1.45, camada 36	2060 ± 85	charcoal	DeBlasis et al. 2007
Az 10637	Locus 1.10, Trincheira 19, camada 11, FS 304	2165 ± 75	charcoal	DeBlasis et al. 2007
Az 09895	Locus 1.35, contato da camada 44 e a base (fim do perfil)	2170 ± 95	charcoal	DeBlasis et al. 2007
Az 09896	Locus 1 Sepultamento 12. Base do perfil	2170 ± 45	charcoal	DeBlasis et al. 2007
Az 10635	Locus 1, Trincheira 17, camada 2, feature 1.05.3, FS 303	2180 ± 105	charcoal	DeBlasis et al. 2007
Az 09893	Locus 1.35, estrato 36 pareada Az10244	2210 ± 60	charcoal	DeBlasis et al. 2007
Az 10634	Locus 1.75, Trincheira 18, camada 10, FS 312	2280 ± 80	charcoal	DeBlasis et al. 2007
Az 09894	Locus 1, camada 7, Sepultamento 3	2500 ± 155	charcoal	DeBlasis et al. 2007
Az 10636	Locus 1, Trincheira 17, camadas 5 a 6, FS 302	2655 ± 105	charcoal	DeBlasis et al. 2007
Az 09899	Locus 2.15 pareada Az10246	2115 ± 65	charcoal	DeBlasis et al. 2007
Az 09898	Locus 2.60	2270 ± 75	charcoal	DeBlasis et al. 2007
Az 09890	Locus 2.15, camada acima do estrato 10, posthole level near burials - pareada Az10245	2285 ± 45	charcoal	DeBlasis et al. 2007
Az 09891	Locus 2, camada 31	2295 ± 90	charcoal	DeBlasis et al. 2007
Az 09889	Locus 2.15 camada 20. Perto da base do perfil	2345 ± 105	charcoal	DeBlasis et al. 2007
Az 09885a	Locus 3, Trincheira 10. Camada superior preta	1850 ± 40	charcoal	DeBlasis et al. 2007
Beta 228507	Locus 3, Trincheira 11. Estrato superior de "preenchimento".	1550 ± 60	charcoal	DeBlasis et al. 2007
Az 09884	Locus 3, Trincheira 11. Camada superior preta	1805 ± 65	charcoal	DeBlasis et al. 2007
Beta 228506	Locus 3, Trincheira 11. Estrato inferior, contato entre camada de conchas e camada escura	1930 ± 50	charcoal	DeBlasis et al. 2007
Az 10632	Locus 5, Trincheira 13, Estrato 7, FS 1 - On the boundary between archaeological and non-archaeological	2310 ± 70	charcoal	DeBlasis et al. 2007
Az 09880	Trincheira 1. Base do sambaqui	2880 ± 75	charcoal	DeBlasis et al. 2007

Az 09881	Trincheira 4. Base do sambaqui	2075 ± 65	charcoal	DeBlasis et al. 2007
Az 09882	Trincheira 5. Base do sambaqui pareada Az10247	2470 ± 55	charcoal	DeBlasis et al. 2007
Az 09883	Trincheira 8. próximo à base do sambaqui	2240 ± 170	charcoal	DeBlasis et al. 2007
Beta 195239	Locus 1.75, perfil leste, camada 2	2070 ± 60	shell	DeBlasis et al. 2007
Az 10243	Locus 1.25, Sepultamento 12. Base do perfil	2365 ± 45	shell	DeBlasis et al. 2007
Az 10244	Locus 1.35, estrato 36 pareada Az9893	2490 ± 35	shell	DeBlasis et al. 2007
Az 10246	Locus 2.15/F2.20.1 pareada Az9899	2335 ± 35	shell	DeBlasis et al. 2007
Az 10245	Locus 2.15, camada acima do estrato 10, posthole level near burials - pareada com Az9890	2370 ± 35	shell	DeBlasis et al. 2007
Az 10631	Locus 5, Trincheira 13, estrato 8, FS 4 - Non-archaeological level	2855 ± 105	shell	DeBlasis et al. 2007
Az 10633	Locus 5, Trincheira 13, Estrato 7, FS 2 - Non-archaeological level	2890 ± 55	shell	DeBlasis et al. 2007
Az 10247	Trincheira 5. Base do sambaqui pareada Az9882	2795 ± 35	shell	DeBlasis et al. 2007
Beta 253672	JABIIG8L05.5 BASE	3200 ± 50	shell (*Anomalocardia flexuosa*)	DeBlasis et al. 2007
Az AA77106	Locus 6, burial 2A	2028 ± 44	human bone	Kneip et al. 2018
Az AA77105	Locus 6, burial 114	2004 ± 44	human bone	Kneip et al. 2018

Stable isotopes of analyzed bones

Table S16: $^{87}Sr/^{86}Sr$ values for Jabuticabeira II skeletons, this study.

Lab ID	Burial ID	DNA	Corr 87Sr/86Sr	Error	84Sr/86Sr	Sr conc (ppm)	88Sr (V)
17973	15	A	0.7094	10	0.05642	158.00	8.11
17979	34	A	0.7094	11	0.05651	174.30	8.17
17977	110	A	0.7095	10	0.05655	223.30	7.48
17980	11a	A	0.7095	9	0.05656	390.30	9.11
17981	2a	A	0.7095	11	0.05654	243.90	8.52
17978	10b	A	0.7095	12	0.05652	219.40	9.25
17972	43	A	0.7097	8	0.05650	145.20	11.21
17974	111/112a	B	0.7103	13	0.05649	233.00	7.82
17975	111/112b	B	0.7106	12	0.05655	167.70	6.92
17976	102	C	0.7111	10	0.05652	296.40	9.14

Archaeological context

Jabuticabeira II is a medium sized shellmound (dimensions: 400×250×10 m) located in Southeastern Brazil and (UTM 22J - 0699479 E; 6835488 S), dated to 3137 – 2794 to 1860 – 1524 cal BP - 2σ[42] – Figure S4. Work at this site was initiated by a binational team including Brazilian and North American members in the late 1990's. Jabuticabeira II is one of the most extensively studied shellmound in Brazil.

This site is the result of incremental complex funerary and feasting rituals, accumulated over centuries[43]. Analyses of the formation processes involved in the construction of Jabuticabeira II showed that the site was built through the performance of feasts and elaborate funerary rituals, the resulting mound becoming part of the regional landscape. Archaeologists identified, along hundreds meters of profiles, several discrete funerary areas (characterized as darker lenses with evidence of postholes and hearths). Within these mortuary locales several individuals were interred. For example, burials 34, 37, 38 and 41 were found in the same funerary area (2.15.13). Similarly burials 10, 11 and 12 were also identified in the same funerary area (1.15.9). This study suggests that these individuals were genetically related. Interestingly, results indicate that the individuals 34 and 43 could be second degree relatives, and the latter's grave is located in a funerary layer immediately below 2.15.13. The fact that a closer genetic affinity can be observed among these groups of individuals suggests that relatives were buried in proximity. - Figures S4 and S5.

Burial 102 caught the team's attention because it was not interred in a flexed position and its grave was located within a shell layer. The deviation from the mortuary pattern along with the individual's genetic affinity with Kaigang groups suggest possible connections between these communities post-2000 BP.

Craniometric studies at this and many other shellmounds in Brazil reveal a morphological pattern different from the earlier groups that inhabited the inland, suggesting genetic drift, as well as coastal migration among coastal populations [45]. Bioarchaeological analyses from Jabuticabeira individuals revealed an abundant and stable marine diet, evidence of some plant domestication, a quite high population density with weaning ages around 2,3 years, presence of infectious diseases but rare violent trauma, [33,42,43,46–49]. The temporal bones of the individuals studied herein (one male, and 4 females), one of the latter possibly affected with treponematosis were contaminated – Figure S5.

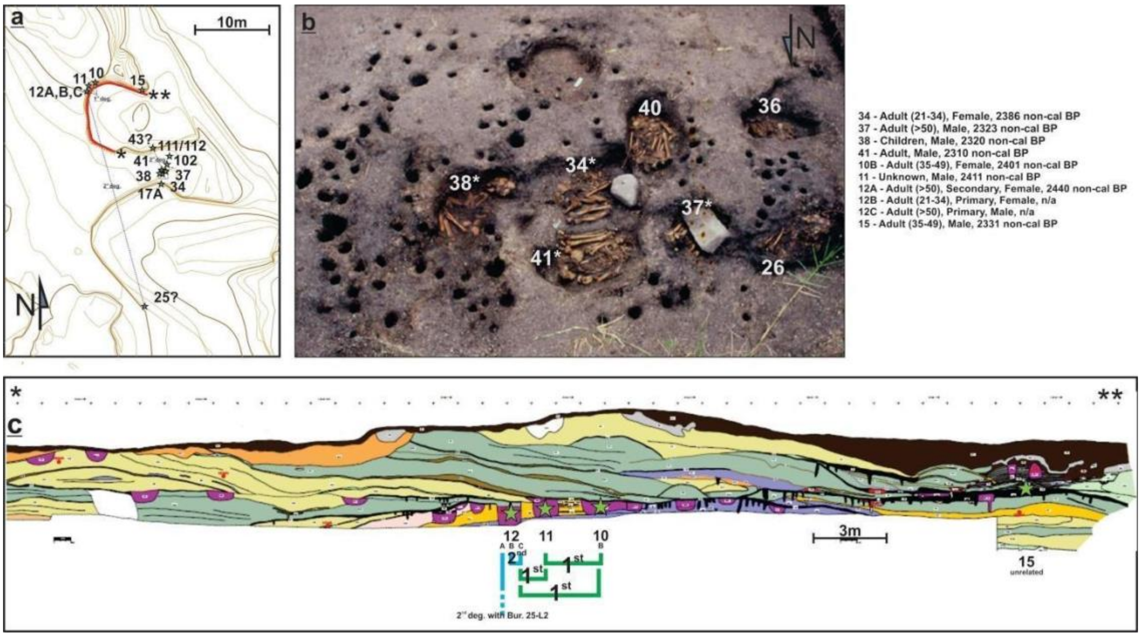

Figure S4: Sambaqui Jabuticabeira II. A) Topography of the funerary areas in Lócus 1 (L1), Lócus 2 (L2), and TL1/L2 with location of the individuals analyzed in this study; B) Photograph of the funerary area in TL1/L2 with location of human burials and information about sex, estimated age, and radiocarbon age; C) Stratigraphic profile of L1 after Simões (2007), with location of individuals analyzed in this study and identification of the ones that are first degree relatives (green star).

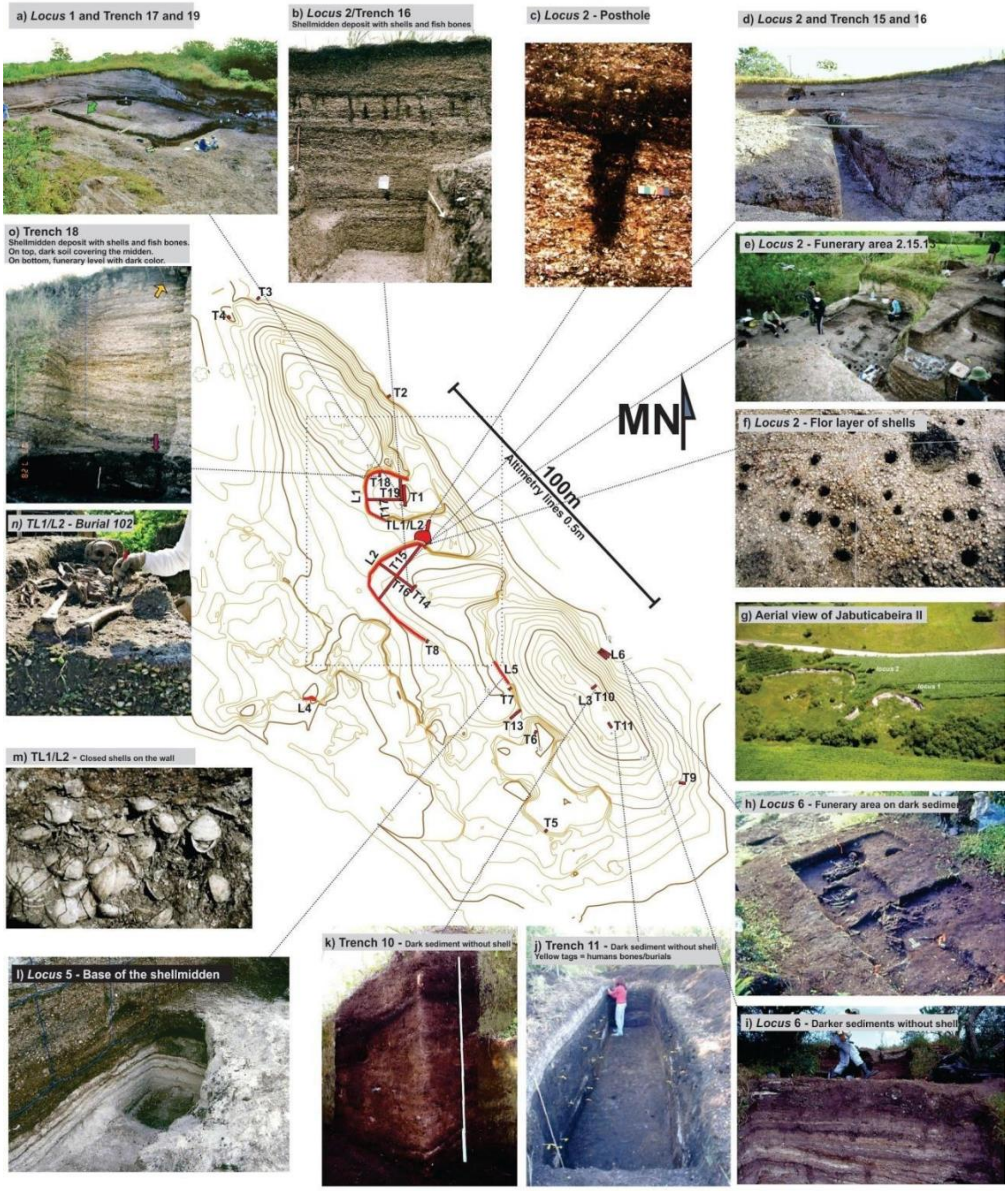

Figure S5: Sambaqui Jabuticabeira II. Site topography with location of excavated areas and profiles (lócus = L, and trenches = T), photographs of selected areas representative of the site's compositional diversity, and details of funerary areas with location of the human burials analyzed in this study.

Vale Do Ribeira De Iguape - Capelinha, Estreito, Laranjal and Pavão

Analyzed Samples

Table S17: Samples from Vale do Ribeira de Iguape region analyzed for aDNA in this study.

IPHAN ID	Site	MPI ID	Context	Bone part	DNA
Capelinha-1 Luzio	Capelinha	CAP007	Burial 2 (Luzio)	pars petrosa	Yes
Capelinha-2	Capelinha	CAP004	Burial 3	tooth	No
Capelinha-3	Capelinha	CAP001	Burial 4	pars petrosa	No
Capelinha-5	Capelinha	CAP002	Burial 5a	pars petrosa	No
Estreito-1	Estreito	EST001	n/a	tooth	No
Laran-1	Laranjal	LAR003	Burial 2	tooth	No
Laran-2	Laranjal	LAR004	Burial 3	tooth	No
Pavao-1	Pavão 16	PVA001	Burial 1	tooth	No
MORAES - 1	Moraes	MOS002	Burial 42	Tooth	No
MORAES - 3	Moraes	MOS003	Burial 25	Tooth	No
MORAES - 4	Moraes	MOS004	Burial 41 A	tooth	No

Chronology

Table S18: Published radiocarbon dates on human skeletons from Vale do Ribeira de Iguape region.

IPHAN ID	LAB ID	Site	Context	^{14}C age [BP]	Source
n/a	Beta 153988	Capelinha I	Burial 2 - Luzio	8860 ± 60	Neves et al. 2005
n/a	Beta 184619	Capelinha I	Burial 5	6090 ± 40	Figuti et al. 2013
n/a	KIA 15561	Moraes	Burial 13	5895 ± 45	Figuti et al. 2013
n/a	KIA 20843	Moraes	Burial 37	5420 ± 30	Figuti et al. 2013
n/a	KIA 15562	Moraes	Burial 5	4985 ± 35	Figuti et al. 2013

n/a	KIA 20844	Moraes	Burial 25	4511 ± 32	Figuti et al. 2013
	MAMS 34575	Moraes	Burial 5	5092 ± 30	Posth et al. 2018
n/a	KIA 20845	Estreito	Burial 1	3655 ± 26	Figuti et al. 2013
n/a	KIA 20846	Estreito	Burial 26	4124 ± 27	Figuti et al. 2013
n/a	KIA 20840	Pavão III	Burial 1	1219 ± 24	Figuti et al. 2013
n/a	KIA 20842	Pavão XVI	Burial 1	1571 ± 24	Figuti et al. 2013
	MAMS 34572	Laranjal	Burial 2	5796 ± 32	Posth et al. 2018
	MAMS 34573	Laranjal	Burial 3	6009 ± 32	Posth et al. 2018

(n/a) not available

Table S19: New radiocarbon dates for human skeletons from Vale do Ribeira de Iguape region, this study. Calibrated using Oxcal 4.4 (Bronk Ramsey 2021) - SHCal20 (Hogg et al. 2020).

IPHAN ID	MPI ID	LAB ID[1]	Context	^{14}C age [BP]	cal BP	C [%]	C:N	Collagen [%]
Capelinha I	CAP007	17754	Burial 2 (Luzio)	9253 ± 25	10552-10291	n/a	n/a	n/a
Capelinha II	CAP004	38995	Burial 3	9254 ± 32	10499-10251	32.7	2.9	1.8
Pavao I	PVA001	39012	Burial 1	1552 ± 22	1469-1314	30.5	2.8	3.2

[1]Curt-Engelhorn-Centre for Archaeometry, Mannheim.

Table S20: Published radiocarbon dates on non-skeletal material from Vale do Ribeira de Iguape region.

LAB ID	Site	^{4}C age [BP]	Material	Source
Beta 189331	Capelinha I	9250 ± 50	shell	Figuti et al. 2013
A 11239	Capelinha I	8795 ± 105	charcoal	Figuti et al. 2013
A 11236	Capelinha I	8500 ± 70	shells	Figuti et al. 2013
Beta 189329	Batatal	9050 ± 100	shell	Figuti et al. 2013
Beta 189337	Laranjal	6980 ± 90	shell	Figuti et al. 2013
Beta189339	Timbuva	5740 ± 50	shell	Figuti et al. 2013
Beta 189330	Alecrim I	5310 ± 50	shell	Figuti et al. 2013
Beta 189332	Capelinha II	5000 ± 70	shell	Figuti et al. 2013
Beta 189333	Capelinha III	4530 ± 50	shell	Figuti et al. 2013
Beta 189334	Capelinha III	4500 ± 40	charcoal	Figuti et al. 2013
Beta 184623	Tatupeva	3990 ± 70	shell	Figuti et al. 2013
Beta 178127	Pavão II	3530 ± 70	shell	Figuti et al. 2013

Beta 189336	Itaoca I	1730 ± 40	charcoal	Figuti et al. 2013
Beta 178126	Itaoca I	1460 ± 60	shell	Figuti et al. 2013
Beta 189335	Gurutuba IV	1650 ± 40	charcoal	Figuti et al. 2013
KIA 20829	Caraça	1607 ± 24	charcoal	Figuti et al. 2013
Beta178125	Caraça	1300 ± 60	shell	Figuti et al. 2013
Beta 178128	Lageado IV	1460 ± 60	shell	Figuti et al. 2013
Beta 184621	Guaracuí	1270 ± 70	charcoal	Figuti et al. 2013

Archaeological context

In the 2000s more than 40 riverine sambaquis were located in the Ribeira de Iguape Valley (southeast São Paulo State) in three geographic areas: Jacupiranguinha Basin; middle-high Ribeira de Iguape Basin (Itaoca area); and Juquiá Basin. Analysis showed a homogeneous life-style, from a cultural point of view [50,51] and similar subsistence strategies [33,53,54], despite occupying distinct environments of the Atlantic forest during almost 9,000 years of cultural continuity.

The riverine shellmound Capelinha is situated at Cajati county (UTM 22J 0778967 E / 7249040 S), in the high portion of the valley (310-320m) at the upper Capelinha river basin, one of the Jacupiranguinha river's affluent, southern tributaries of the Ribeira de Iguape river. The site is settled in a small terrace in a mountainous landscape, with a low slope to the east and an abrupt slope to a headwater stream. The site does not exhibit a mounded shape, it is flat and composed by shell layers that have filled the concavities and irregularities of the slope. According to previous owners, the shellmound was mostly razed. Initial prospections and pit-tests indicated it was occupied in three different periods by distinct cultures: 1) Umbu tradition hunter-gatherers; 2) riverine sambaqui builders; 3) Itararé-Taquara ceramists. Around the shell deposits there is a thin archaeological soil (less than 20 cm) with small pockets of shells. Site chronology starts at 9250 ± 50 BP until 6090 ± 40 BP. From the excavation area (40m^2) five recognizable burials and a few dispersed human bones were recovered.

The Estreito site is located in Adrianópolis county, Paraná State (UTM 22J 0716887 / 7269427). This site is situated on a river terrace near the Ribeira de Iguape river, medium-high basin, at 150 m.a.s.l. It presents a rounded morphology, with 800 m^2 of estimated area 2 m high. The site was cut by a road exposing a large stratigraphic profile. Analysis of the site's stratigraphy indicated four layers: three upper layers combining dark-brown sediments made of sand, shells and charcoal; and a bottom layer made of yellow sandy-loam sediment. Eight human burials were described in the stratigraphic profile and surface surveys, dispersed in the four stratigraphic layers. Six burials were recovered from the site, with ages between 4124 ± 27 BP and 3655 ± 26 BP.

The site Pavão XVI is located in the Itaoca county, state of São Paulo (UTM 22J 718349 / 7273199), at altitude 150 m.a.s.l. The site is within the urban area and it was partially razed with few houses built on top. Surveys located a shallow archaeological deposit, of 40 cm thickness, made of sand and shells of land snails. Three human burials were described, but only one was recovered and dated to 1571 ± 24 BP.

Capelinha is the earliest riverine sambaqui in the Ribeira de Iguape Valley, representing the initial phase of shellmound settlement, and already showing cultural characteristics that persisted in other sites over the 9000 years.

Sambaqui do Limão

Analyzed Samples

Table S21: Samples from sambaqui do Limão analyzed for aDNA in this study.

IPHAN ID	MPI ID	Context	Bone part	DNA
ES- 01	EPS001	Burial id not available. Box 8.680 kg, cabinet 3, bookshelf, shelf 4, left temporal bone, weight 40.18 gr	pars petrosa	Yes
ES-02	EPS002	Burial id not available. Box 8.680 kg, cabinet 3, bookshelf 4, shelf 4, left temporal bone, weight 40.15 gr	pars petrosa	Yes
ES-03	EPS003	Burial 3. Box 1,354 kg, cabinet 2, bookshelf 3, shelf 3, right temporal bone, weight 39.19 gr	pars petrosa	Yes
ES-04	EPS004	Burial id not provided. Box 8.680 kg, cabinet 3, bookshelf 3, shelf 4, left temporal bone, weight 53.21 gr	pars petrosa	Yes
ES-05	EPS005	Burial id not available. Box 8.680 kg, cabinet 3, bookshelf 4, shelf 4, temporal and occipital bone, weight 122.13 gr	pars petrosa	Yes
ES-06	EPS006	Burial id not available. Box 8.860 kg, cabinet 3, bookshelf 4, shelf 3, maxilla, weight 109,79	tooth	No
ES-07	EPS007	Burial id not available. Box 8.580 kg, cabinet 3, bookshelf4, shelf 3, mandible, weight , 31,75 gr	tooth	No
ES-08	EPS008	Burial id not available. Box 1.354 kg, cabinet 2, bookshelf 3, shelf 3, mandible, weight 127,10 gr	tooth	No
ES-09	EPS009	Burial id not available. Box 8.680 kg, cabinet 3, bookshelf 4, shelf 4, mandible, weight 38,68	tooth	No
ES-10	EPS010	Burial id not available. Box 8.580 kg, cabinet 3, bookshelf 4, shelf 3, mandible, weight 48,75 gr	tooth	No
ES-11	EPS011	Burial id not available. Box 8.680 kg, cabinet 3, bookshelf 4, shelf 4, maxilla, weight 30,61 gr	tooth	Yes

Table S22: New radiocarbon dates for human skeletons from sambaqui do Limão, this study. Calibrated using Oxcal 4.4 (Bronk Ramsey 2021) - SHCal20 (Hogg et al. 2020).

IPHAN ID	MPI ID	LAB ID[1]	Context	^{14}C age [BP]	cal BP	C [%]	C:N	Collagen [%]
ES-01	EPS001	34571	n/a	448 ± 25	528-479	29.1	2.8	5.1
ES-04	EPS004	40649	n/a	1914 ± 19	1883-1744	37.2	3.2	3.5
ES-03	EPS003	40648	n/a	1976 ± 18	1985-1835	47.0	3.2	1.8
ES-05	EPS005	40650	n/a	2025 ± 18	2002-1892	42.1	3.2	1.7
ES-02	EPS002	40647	n/a	2617 ± 19	2760-2729	28.7	3.3	0.5

[1] Curt-Engelhorn-Centre for Archaeometry, Mannheim. (n/a) not available

Archaeological context

Discovered during the 1950/60s, the sambaqui do Limão (a.k.a. Sambaqui da Ilha do Limão) is located in the northern area of the Vitória Bay (Espírito Santo State), within the hydrographic basin of the Santa Maria River (UTM 24 K 365312 / 7761479; WGS84). With almost 10 meters height, this shellmound was located at the edge of a mangrove, turning it into a dry refugium in the landscape during flooding episodes (Figure S6). In 1982 a transmission line for electric energy was constructed on top of the site, leading to its almost complete destruction. As part of an emergency rescue, several skeletal remains were collected from the site. Field descriptions indicate complete skeletons of single individuals buried with grave goods including projectile points and lip ornaments (tembetás), both produced with polished bone, as well as shell beads. One individual was located at the bottom of the midden and was properly documented in the field, however, the skeletal collection includes many other individuals without field descriptions. Early descriptions of the site mentioned a layer of 'black earth', but it is uncertain whether it is analogous to the dark layers made of fish bones (without shells) that are commonly found on top shellmound in southern Brazil (like Jabuticabeira II), or if it corresponds to soil development on top of the shellmound.

The skeletal remains from sambaqui do Limão were never curated, individualized, sexed or analyzed. The remains are stored at the IPHAN-ES, the federal agency for cultural heritage. The radiocarbon ages in this study are the first obtained in human remains not just from sambaqui do Limão, but for the entire state of Espírito Santo. The site chronology indicates an occupation beginning at ca. 2700 cal BP until ca. 500 cal BP, encompassing both the typical sambaqui horizon and the post-2000 BP horizon of cultural change that is described for the southern coast. However, there is no description of pottery at the site.

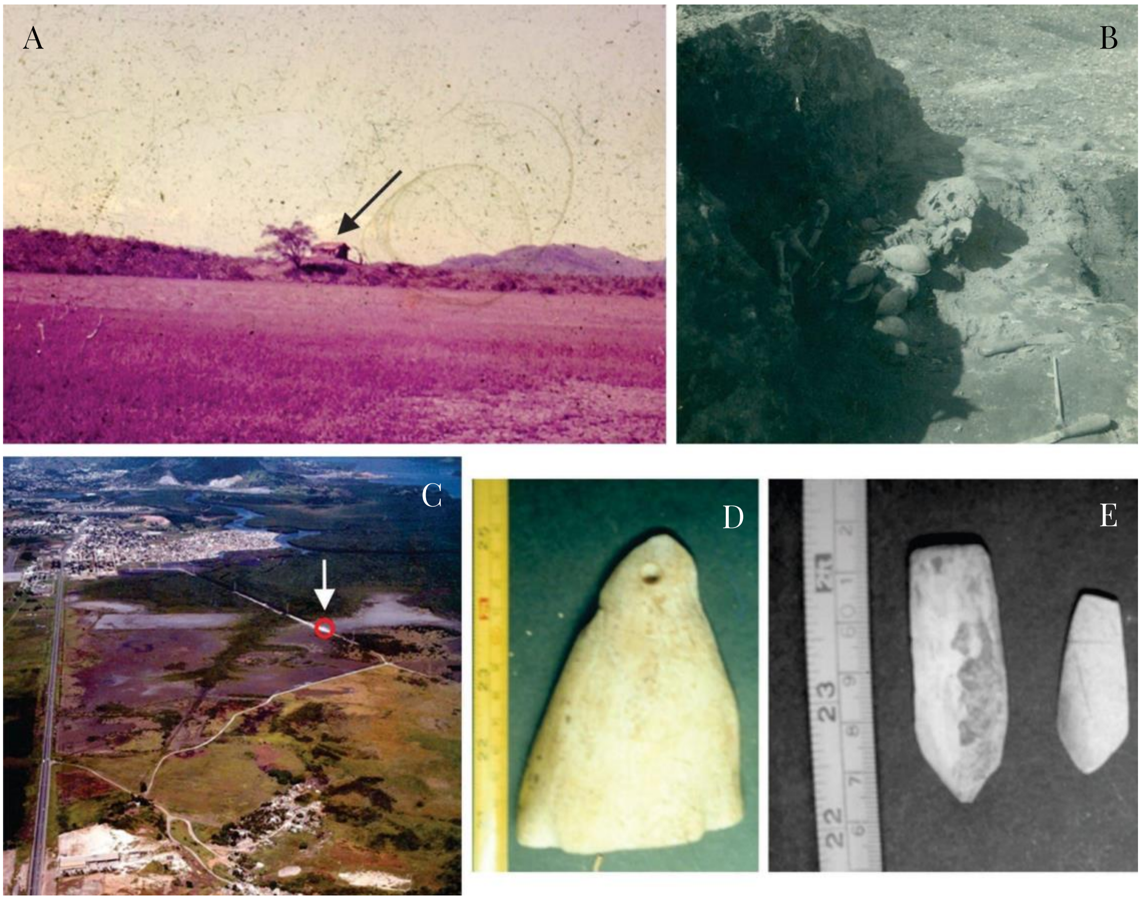

Figure S6: Sambaqui do Limão. A) Photograph of a house built on top of the site (Celso Perota collection, 1969); B) Sambaqui do Limão – Burial 1. The complete skeleton of an adult individual was found in left lateral decubitus during salvage excavations in 1982 in what was left of the shellmound (Photograph by Celso Perota, 1982); C) Aerial view with location of sambaqui do Limão (red circle) before it was almost completely destroyed by the construction of an energy line (visible as a diagonal white line) (Photograph by Celso Perota, 1982); D) Necklace bead made of gastropod shell (Photograph by Celso Perota, 1982); E) Lip ornaments on bone (Photograph by Celso Perota, 1982).

Region of Pains: Loca do Suin

Analyzed Samples

Table S23: Samples from Loca do Suin analyzed for aDNA in this study.

IPHAN ID	MPI ID	Site	Context	Bone part	DNA
Pains - 04	LDS001	Loca do Suin	Burial 1A	pars petrosa	No
Pains - 05	LDS002	Loca do Suin	Burial 1B	tooth	No
Pains - 06	LDS003	Loca do Suin	Burial 1A	tooth	No
Pains - 19	PAI001	Loca do Suin	Burial 01C #19	pars petrosa	Yes

Chronology

Table S24: Published radiocarbon dates on human skeletons and non-skeletal material from Loca do Suin.

IPHAN ID	LAB ID	Context	^{14}C age [BP]	Material	Source
n/a	Beta 210401	Burial 2	7530 ± 50	human bone	Strauss et al. 2011
n/a	Beta 210400	Burial 1	7440 ± 50	human bone	Strauss et al. 2011
n/a	Beta 210726	n/a	1270 ± 40	charcoal	Koole 2007

(n/a) not available

Table S25: New radiocarbon dates for human skeletons from Loca do Suin, this study. Calibrated using Oxcal 4.4 (Bronk Ramsey 2021) - SHCal20 (Hogg et al. 2020).

IPHAN ID	MPI ID	LAB ID[1]	Context	^{14}C age [BP]	cal BP	C [%]	C:N	Collagen [%]
Pains-5	1705	28708	LocaSuin - Burial 1B	8172 ± 32	9264-8996	32.1	3.3	1.75
Pains-6	1706	28709	LocaSuin - Burial 1A	8126 ± 36	9257-8778	34.6	3.3	3.80

[1] Curt-Engelhorn-Centre for Archaeometry, Mannheim.

Archaeological context

Loca do Suin is a small rockshelter located in the region of Pains, state of Minas Gerais (UTM 23K 417314 / 7752410). Excavations in 2003 and 2004 identified two human burials, lithics, pottery and animal bones [55,56] (Figure S7). Burial 1 was a multiple interment including two adults, one child and one stillborn. One of the adult individuals was cremated. Burial 2 included the complete and highly

fragmented skeleton of an adult individual. An unburned fragment of a rib bone from one of the adult individuals in Burial 1 was directly dated to 7440 ± 50 BP (Beta-210400). A rib fragment from Burial 2 was directly dated to 7530 ± BP (Beta-210401).

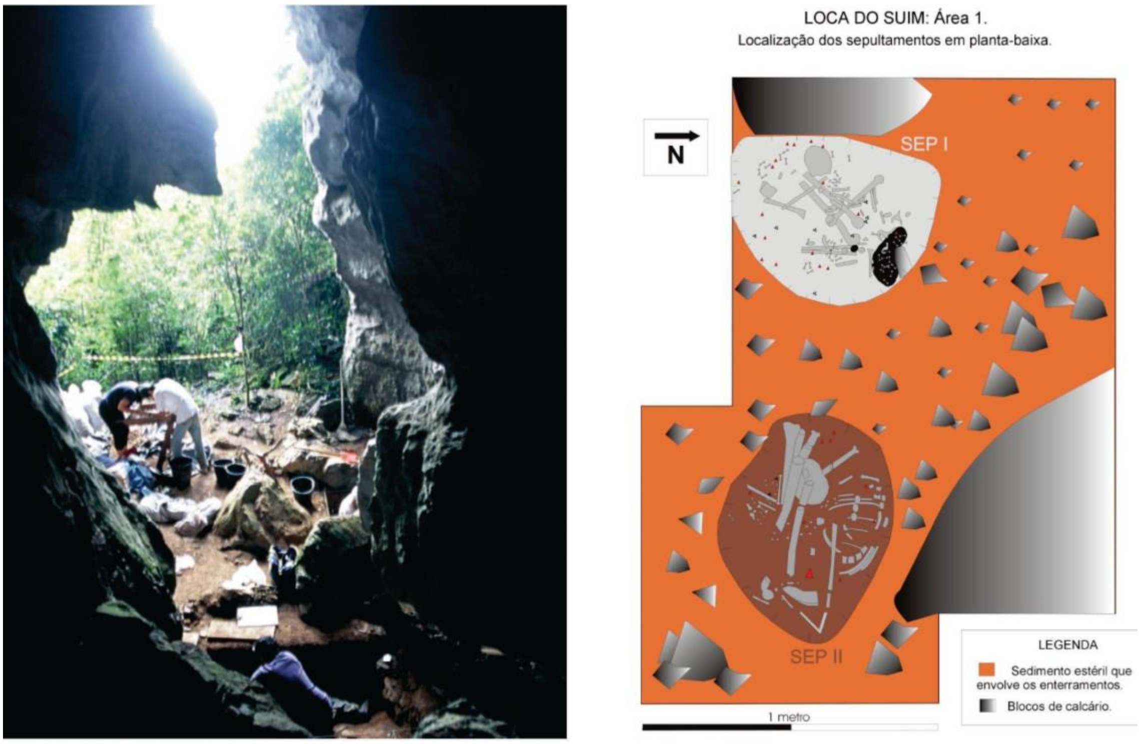

Figure S7: Loca do Suin. Excavations at Loca do Suin (left) (Photograph by Cyro Soares), and schematic representation of Burial 1 and Burial 2 (right) (Drawing by Edward Koole).

Pedra do Alexandre

Analyzed Samples

Table S26: Samples from Pedra do Alexandre analyzed for aDNA in this study.

IPHAN ID	MPI ID	Context	Bone part	DNA
ALE - 01	TDA002	Burial 2	pars petrosa	Yes
ALE - 02	TDA005	Burial 7a	pars petrosa	No

Table S27: Published radiocarbon dates on non-skeletal material from Pedra do Alexandre.

LAB ID	Context	^{14}C age [BP]	Material	Source
CSIC-1061	Burial 7	2620 ± 60	charcoal	Silva & Solari 2020
CSIC-0966	Burial 9	2890 ± 25	charcoal	Silva & Solari 2020
CSIC-0945	Burial 9	2860 ± 60	charcoal	Silva & Solari 2020
CSIC-1054	Burial 2	4160 ± 70	charcoal	Silva & Solari 2020
CSIC-0943	Burial 1	4710 ± 25	charcoal	Silva & Solari 2020
CSIC-1060	Burial 6	5790 ± 60	charcoal	Silva & Solari 2020
CSIC-1052	Burial 1	6010 ± 60	charcoal	Silva & Solari 2020
CSIC-0965	Burial 4	8280 ± 30	charcoal	Silva & Solari 2020
CSIC-0967	Burial 3	9400 ± 35	charcoal	Silva & Solari 2020
CSIC-1051	Burial 3	9400 ± 90	charcoal	Silva & Solari 2020
Beta 327693	Burial 23	9449 ± 40	charcoal	Mafra 2020
Beta 327694	Burial 25	9100 ± 50	charcoal	Mafra 2020
Beta 327730	Combustion structure	2170 ± 30	charcoal	Mafra 2020
Beta 327695	Burial 26	9170 ± 40	charcoal	Mafra 2020
Beta 327729	Close to iron oxide with use marks	9140 ± 50	charcoal	Mafra 2020

Archaeological context

Pedra do Alexandre (a.k.a. Abrigo do Alexandre or Pedra do Chapéu) is a rockshelter located at Carnaúba dos Dantas in the state of Rio Grande Norte, northeastern Brazil (6˚32'43''S and 36˚31'10'' W) [57,58]. A total of 24 human burials were excavated from the site including single and multiple interments totalizing 48 individuals. Combustion structures, grave goods in the form of personal ornaments, knapped lithic artifacts, polished stone artifacts and pottery were all recovered from the site. In the absence of collagen, the chronology of the skeletons was established on ten radiocarbon dates of charcoal samples, indicating a long sequence of occupation starting at ca. 9400 until ca. 2600 BP. Burial 2, from which DNA was successfully extracted, is dated by association to 4160 ± 70 BP (CSIC-1054)

(Figure S8). This particular burial consists of a single complete skeleton of a young adult male deposited in left lateral decubitus. Grave goods accompanied the interment.

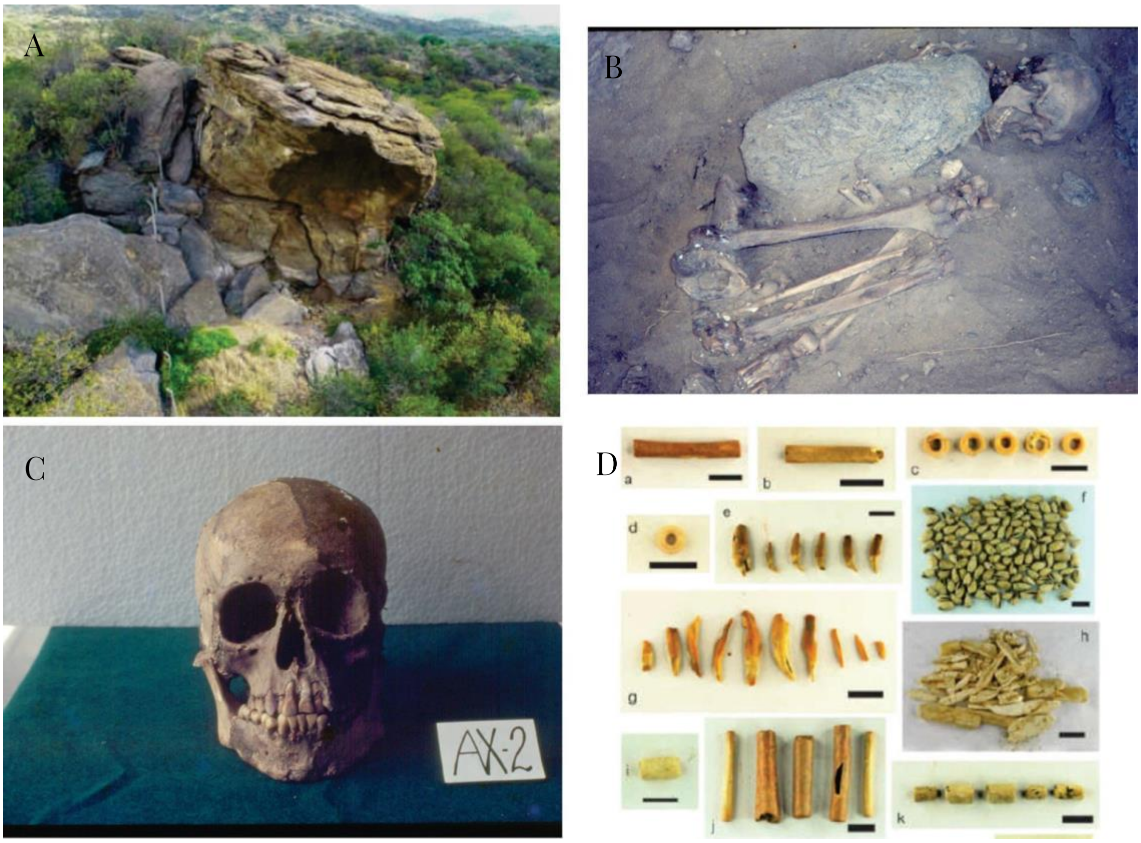

Figure S8: Pedra do Alexandre. A) Aerial view of Pedra do Alexandre Rockshelter; B) Burial 2 during excavation; C) Cranium of Burial 2; D) Grave goods commonly found in association with the skeletons at Pedra do Alexandre, although not specifically with Burial 2.

Palmeiras - Xingu

Analyzed Samples

Table S28: Samples from Palmeiras Xingu analyzed for aDNA in this study.

IPHAN-ID	MPI-ID	Context	Bone part	DNA
Palmeira 100	PLM002	Burial 1	tooth	No
Palmeira 121	PLM001	Burial 2	pars petrosa	Yes

Chronology

Table S29: New radiocarbon date for human skeleton from Palmeiras Xingu, this study. Calibrated using Oxcal 4.4 (Bronk Ramsey 2021) - SHCal20 (Hogg et al. 2020).

IPHAN ID	MPI ID	LAB ID[1]	Context	^{14}C age [BP]	cal BP	C [%]	C:N	Collagen [%]
Palmeira 121	PLM001	43998	Burial 2	477 ± 21	535-500	30.6	3.3	0.8

[1] Curt-Engelhorn-Centre for Archaeometry, Mannheim.

Archaeological context

The Palmeiras-Xingu archaeological site is located on the left margin of the lower Xingu river (Volta Grande do Rio Xingu) in northeast Amazonia, Brazil (UTM SAD69 22M 352550 / 9617460) [59]. Excavations at the site were carried out within the environmental license process for the construction of the Belo Monte Hydroelectric Power Plant, and revealed three human burials. Two were excavated and consisted of primary single burials of adult males with flexed legs in right lateral decubitus and no funerary goods. The site occupies an area of approximately 256.700 m² and has a layer of Anthropogenic Dark Earth (ADE) up to 60 cm thick from the surface. The spatial distribution of the ADE mounds indicates that a ring village existed at the location. The three burials were found within the ADE layer. The site was occupied multiple times by ceramist groups from 2,290 to 477 BP (unpublished). The ceramic collection presents a rich variety in the morphology of the pottery and in the plastic and painted decoration, with characteristic attributes that relate to the known Koriabo and Tupiguarani ceramics (Figure S9).

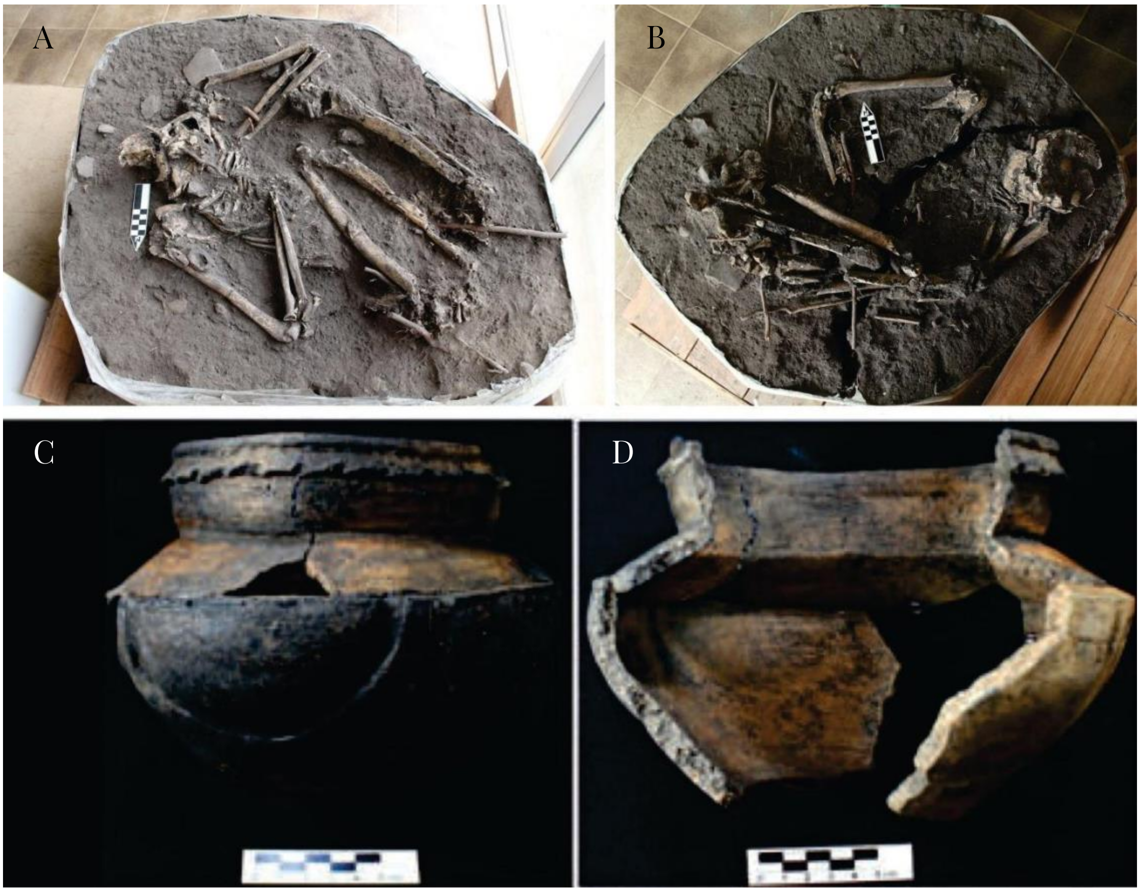

Figure S9: Palmeiras - Xingu. A) Burial 1; B) Burial 2; C) Koriabo tradition vessel from the nearby site of Villa Rica 2; D) Koriabo tradition vessel from the nearby site of Villa Rica 2.

The Koriabo tradition is a unique late precolonial/early colonial (c. 1200-1600 CE) archaeological culture characterized by a ceramic style found throughout northeastern South America (except from Venezuela), including the Guianas highlands, the Lesser Antilles (locally known as Cayo complex) and the middle/lower Xingu River, where Palmeiras-Xingu is located [60,61]. The Koriabo ceramics are characterized by ‘toric’ jars (necked pot with multiangular body) and ‘flower’ bowls (unrestricted mouths and carinated body with polylobed rim). The decoration exhibits curvilinear motives executed with two types of incisions (fine and scraped), appliqué nubbins with concentric punctuation made with hollow instruments and plainor finger pressed strips. Anthropomorphic and zoomorphic appendages are also common in the Koriabo pottery. Red and polychrome painting – black, red, yellow and/or orange – on white slip is found in some highly elaborated pieces. Although the majority of Koriabo pottery are shreds, complete vessels are not rare and were usually used as funerary urns (although not at Palmeiras - Xingu). Houses and village layouts (postholes) are also found associated with Koriabo pottery [62,63].

The ethnic/linguistic affiliation of the producers of Koriabo ceramics is highly debated. A Cariban-speaking affiliation has been proposed on the following basis: 1) geographical overlap; 2) similarities between Koriabo and Kalina pottery, a Caribbean-speaking group from the Guiana

Highlands [60]; 3) similarities between Cayo archaeological sites and Kalínago (Island Carib) houses and villages layouts, a Cariban-speaking group described in the 17th century [64].

The Koriabo-Karib association, however, is not universally accepted. Although the overall areas of occurrence of Koriabo pottery and Cariban-speaking groups do grossly coincide, there were also non-Caribam-speaking groups living in those areas [60,65]. Concerning stylistic similarities, it has been noted that Koriabo ceramics also share attributes with pottery produced by Arawakan-speaking groups (e. g. Lokono) [66]. Although the Kalina are a Cariban-speaking group, their pottery style seems to have emerged from the fusion and interaction of different native groups during colonial times. The Koriabo pottery may not, therefore, represent a distinct ethnic/linguistic group but instead multilinguistic and multiethnic communities [64,67].

The presence of non-Koriabo pottery in Koriabo sites is relatively common suggesting permeable frontiers, trade networks and interaction spheres across the entire region[66–68]. In Guayana, Koriabo sites often contain ceramics of the Kwatta and Barbakoeba, other archaeological cultures often associated with Cariban-speaking groups (Incised-Punctated tradition) [60]. In the Antilles, Cayo pottery is considered a hybrid style combining Koriabo elements with others from the Greater Antilles [69]. Cayo is an early colonial phenomenon related to the fusion of different peoples in the process of European colonization. In the Middle Xingu region, Palmeiras - Xingu included, Tupiguarani pottery is often found within Koriabo deposits and vessels [65]. Concerning pottery similarities, it has been noted that Koriabo also shares stylistic attributes with pottery produced by Arawak-speaking groups, with morphological convergences like rim flanges, carinate profiles and red-on-white painting) [59,70]

To the best of our knowledge, this study is the first to present genetic data for a skeleton coming from a Koriabo context. When compared to the Native American populations available on the database, the Koriabo individual shows stronger genetic affinities with the Arara, a Cariban-speaking group from the lower Amazon. However, more Koriabo individuals and contexts must be analyzed before solid conclusions can be drawn. Nevertheless, it is striking that the genetic data seems to align with the most common ethnic association proposed in the literature for the producers of the Koriabo ceramics. It seems that the spread of Koriabo pottery outside the Guianas Highlands was not a process entirely based on a 'shared ideology' [59,63], but instead included - at least to a certain extent - a demic component and the movement of individuals affiliated with Cariban-speaking groups.

Kaingang Mound

Analyzed Samples

Table S30: Samples from the Sakai collection analyzed for aDNA in this study.

IPHAN ID	MPI ID	Site	Context	Bone part	DNA
SK - 14	SAM014	Kaingang burial	Jaw Sk-hu-2026 - Jaw - Sk-hu-2026	tooth	Yes
SK - 15	SAM015	Kaingang burial	Upper-jaw Sk-hu-2091 - Jaw - Sk-hu-2091	tooth	No

Chronology

Table S31: New radiocarbon date for human skeleton of Sakai collection, this study. Calibrated using Oxcal 4.4 (Bronk Ramsey 2021) - SHCal20 (Hogg et al. 2020).

IPHAN ID	MPI ID	LAB ID	Context	^{14}C age [BP]	cal BP*	C [%]	C:N	Collagen [%]
SK-14	1709	28711	Kaingang - upper-jaw Sk-hu-2026	100 ± 22	251-6	42.3	3.3	16.74

[1] Curt-Engelhorn-Centre for Archaeometry, Mannheim. * Date probably out of range: 251BP (7.4%) 227BP, 140BP (21.0%) 110BP, 105BP (7.0%) 80BP, 74BP (58.8%) 20BP, 14BP (1.3%) 6BP.

Archaeological context

Kiju Sakai was a Japanese anthropologist and archaeologist who came to Brazil in 1934[71]. Throughout his life, he excavated many sites and formed an important archaeological collection. The “Archaeological Collection Kiju Sakai" includes the most distinct types of pre-colonial and colonial era archaeological material, including: ceramics of Tupiguarani and Jê groups; and flaked and polished stone artifacts, projectile points made of iron and medals of the Serviço Nacional de Proteção ao Índio (the National Bureau for the Protection of Native Brazilians). Of particular relevance for this study are the human skeletons that Sakai recovered from the riverine shellmound do Alecrim, located in the Vale do Ribeira de Iguape (same location as the riverine sambaquis included in this study, such as Capelinha, Estreito and Pavão I). Sakai also excavated human skeletons from late 19th century funerary mounds of the Kaingang, located in th western part of the state of São Paulo. The Kaingang are a thriving ethnic Jê-speaking group comprising more than 45000 individuals inhabiting large areas of southern Brazil. It

is assumed that the Kaingang descend from the populations that occupied the highlands of southern Brazil from ca. 3000 years onwards that are associated with pit houses, ‘danceiros’ (a.k.a. geoglyphs) and the Taquara-Itararé ceramics [31] (Figure S10).

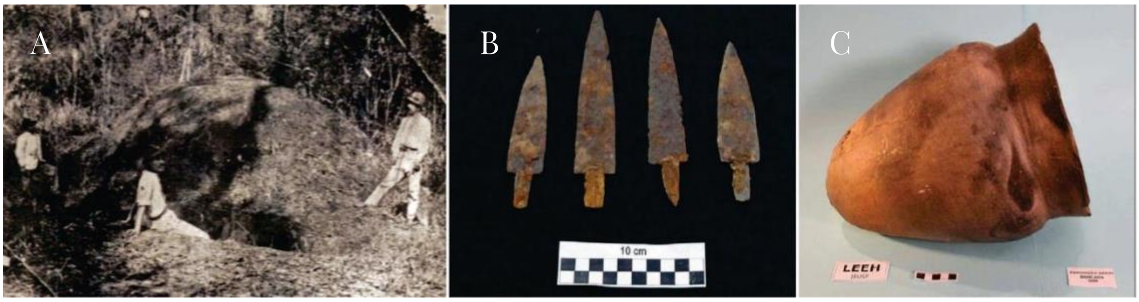

Figure S10: Kaingang Mound and Sambaqui do Alecrim. A) Excavation of a Kaingang burial mound by Kiju Sakai. B) Projectile points made of iron found within the burial mound. C) Pottery vessel typically produced by Kaingang groups.

Vau 1 - Una

Analyzed Samples

Table S32: Samples from the site Vau 1 analyzed for aDNA in this study.

IPHAN ID	MPI ID	Site	Context	Bone part	DNA
PUC_GO-24	VSM001	Vau 1	Individual ID not confirmed, it was located in the same box as Ind 23.	pars petrosa	Yes

Chronology

Table S33: New radiocarbon date for human skeleton of Vau 1, this study. Calibrated using Oxcal 4.4 (Bronk Ramsey 2021) - SHCal20 (Hogg et al. 2020).

MPI ID	LAB[1] ID	Context	14C age [BP]	cal BP	C [%]	C:N	Collagen [%]
VSM001	45782	No ID, but in the same box as Individual 23.	629 ± 21	655-554	35.1	3.3	2.2

[1]Curt-Engelhorn-Centre for Archaeometry, Mannheim.

Archaeological context

Vau 1 (BA-RC-55) is an open-air site located at the southeast region of the state of Bahia (UTM 23L 544307.75 / 8537761.66) that was identified by Public Archaeology activities during construction work at Vau, a community in the municipality of Santa Maria da Vitória [72]. The human skeletal remains of Vau 1 include individuals of both sexes and different ages. They were interred both directly in the soil and inside ceramic urns. The skeletons were accompanied by grave goods such as beads made of vegetable material and pottery objects[73]. The pottery from Vau 1 – urns included – is characterized by thin-walled small globular vessels with smudging polished (dark staining obtained from exposure to smoke and soot) on the outer walls and no plastic or polychrome painted decoration. In conjunction, these characteristics allow us to assign the pottery from Vau 1 to the Una tradition – (Figure S11).

The Una tradition marks one of the earliest introductions of ceramic technology in Brazil south of the Amazon by around ca. 2500 BP. Although domesticated plants were already present in this region by at least 4000 BP[74], the arrival of the Una tradition certainly reflects a significant change in subsistence strategies with an intensification of horticulturalism [75,76]. It is not known if pottery was brought from the Amazon by migrating groups or adopted by the local foragers that inhabited the region since the early Holocene [76]. Although pottery of the Una tradition occurs in large areas of Brazil, its techno-cultural particularities (i.e. simplicity and generalized technology) makes it unlikely to represent a single population/cultural phenomenon. Pottery vessels of the Una tradition are commonly found within rockshelters, and archaeological sites are typically small, suggesting low demographic density.

The Una tradition persisted until colonial times, co-existing with late ceramic traditions that appear in the same region from ca. 900 AD (e. g. Aratu tradition, Uru tradition). These later pottery traditions are similar to the Una ceramics, lacking complex morphologies and polychrome painting, in sharp contrast with their Amazonian counterparts and later derivatives (i.e. Tupiguarani tradiction). On the other hand, the ceramic vessels of the Aratu and Uru traditions are large in size - reaching up to 450 liters – and commonly associated with large ring villages that were inhabited by up to 1500 individuals [77]. The interactions between Una groups and the dwellers of the ring villages are poorly understood, although it is clear that no complete substitution took place as Una sites persisted until the colonial period.

The producers of the Aratu tradition were usually presumed to be the ancestral of the Jê-speaking populations that inhabited a large portion of central Brazil [76,78]. The ethno/linguistic affiliation of the producers of the Una tradition is less clear as this kind of pottery occurs in a wide range, both in time and space [79]. However, considering this is a non-Amazonian techno-cultural complex, it is not impossible that Una pottery was produced – at least in part – by ancestors of macro-Jê speaking groups. To the best of our knowledge, the present study is the first to present genetic data for a skeleton coming from an Una context. Accordingly, among the Native American populations available for comparison, the strongest similarities are with the Xavánte, a Jê-speaking group from Central Brazil. This is the first

direct evidence connecting groups producing simple non-decorated pottery with Jê-speaking related ancestry.

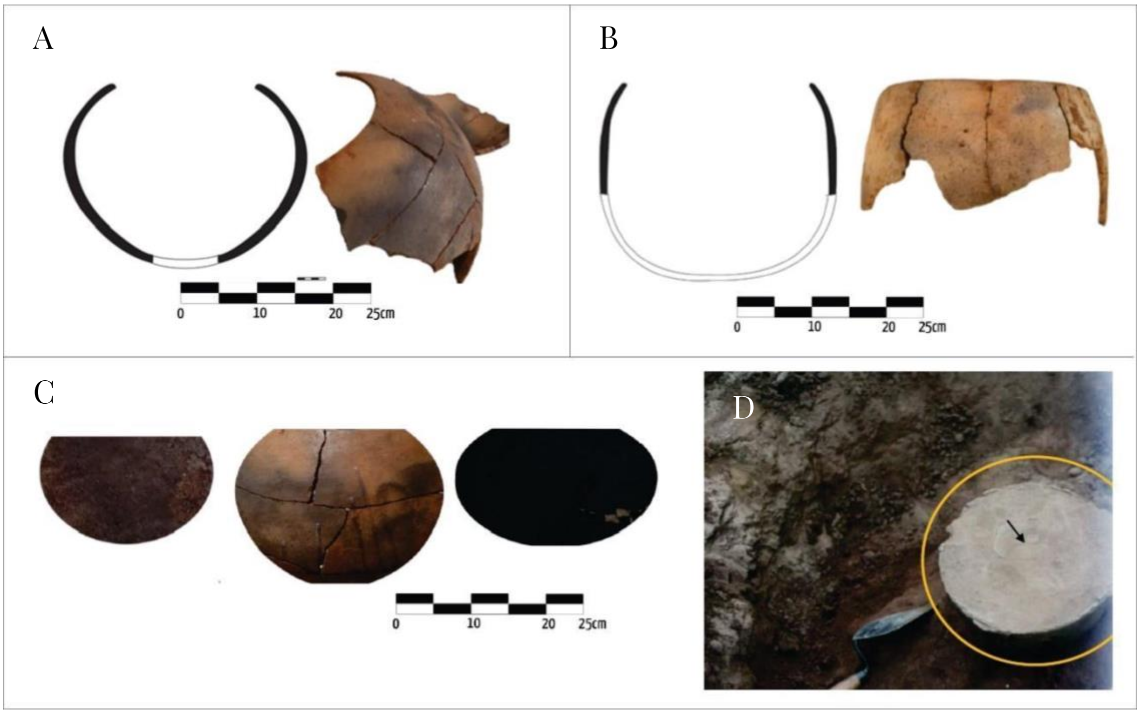

Figure S11: Vau 1 - Una. A-B) Una ceramics characterized by thin-walled, small, globular vessels without plastic or painted decoration, but with the presence of smudging polished; C) Reconstruction of funerary urns; D) field picture showing human bones within ceramic urn.

Sites without sufficient ancient DNA preservation

Xingó Cemeteries – Justino, São José II and Jerimum

Analyzed Samples

Table S34: Samples from the region of Xingó analyzed for aDNA in this study.

IPHAN ID	MPI ID	Site	Context	Bone part	DNA
MAX-06	JUS001	Justino	Bur. 161 – Graveyard D – Adult	pars petrosa	No
MAX-13	JUS002	Justino	Bur. 162 – Graveyard C – Sub-adult	pars petrosa	No
MAX-19	JUS003	Justino	Bur. 139 – Graveyard B – Adult	pars petrosa	No
MAX-23	JUS004	Justino	Bur. 53 – Graveyard A – Sub-adult	pars petrosa	No
MAX-25	SJ2001	São José II	Bur. 20 – Sub-Adult	pars petrosa	No
MAX-28	JER001	Jerimum	Bur. 4 – Adult	pars petrosa	No
MAX-32	JER002	Jerimum	Bur. 8 – Adult	pars petrosa	No

Archaeological context

Justino, São José II and Jerimum are cemetery sites which were identified in the Xingó region, Lower São Francisco River, North-eastern Brazil. Xingó is characterized by a landscape of granite and magnetite canyons, which are cut by the São Francisco River and its intermittent tributaries, in a region covered by the dry vegetation known as Caatinga. These sites are located at river terraces where the São Francisco join its small tributaries. The geomorphological location, at the base of escarpments, resulted in high content of sediments (alluvial and colluvial) at these sites: archaeological materials were found over 6m in depth in all three [80–82]. Unfortunately, Justino and São José II have been flooded by the construction of the Xingó Dam.

Since pre-colonial times up to the present, fluvial terraces have been the most sought places for human settlement in Xingó, and Justino possesses the earliest dates of the region: ca. 8.900 BP [82]. Although the end of the chronology of the site is uncertain, glass beads identified at some of the burials [83] provide evidence that the cemetery was still active during the beginning of colonial times. This site also presented the greatest number of burials in the region: a total of 167 individuals divided in four

hypothetical cemeteries [84]. Burials associated with ceramics have an early ^{14}C date of 5570 ± 70 BP [82]. Besides ceramic vessels and sherds, the primary and secondary burials were accompanied by an elaborated set of objects, such as bone pendants, bracelets, and musical instruments (i.e., flutes), animal burials, amazonite adornments, clay pipes and lithic artifacts [85].

São José II site has only two ^{14}C dates: 4140 ± 90 and 3500 ± 110 BP. The excavation of the cemetery yielded 29 burials. Besides human remains, the site presented lithic and ceramic artifacts, faunal remains (bones and shells), and is located close to a shelter with rock paintings [81]. The last site, Jerimum, is the only site which has no absolute dates. The excavation of this site presented ten graves with a total of eleven individuals. Lithics, ceramics and faunal remains (shells and bone) were recovered. The Jerimum burials, all primary, were described as having extremely poor preservation and a high level of fragmentation [86]. Some of the burials included adornments, such as bone and shell pendants. The presence of unmodified stones (isolated or in hearths), gastropod and bivalve shells within the burial features, and the similarity between bone pendants, seem to be elements that link these three cemetery sites [80]. Moreover, the predominance of Xingó ceramics in them also suggests a cultural linkage between sites [86].

Lapa do Santo

Analyzed Samples

Table S35: Samples from the site of Lapa do Santo analyzed for aDNA in this study.

IPHAN ID	MPI ID	Context	Bone part	DNA
Lapa 011	LAP013	Burial 11	pars petrosa	No
Lapa 003	LAP012	Burial 3 (Individual #2, PN-01.08.0637)	pars petrosa	No

Chronology

Table S36: New radiocarbon date for human skeletons from Lapa do Santo, this study. Calibrated using Oxcal 4.4 (Bronk Ramsey 2021) - SHCal20 (Hogg et al. 2020).

MPI ID	LAB ID[1]	Context	^{14}C age [BP]	cal BP	C [%]	C:N	Collagen [%]
LAP013	40652	Burial 11	8405 ± 42	9524-9150	25.5	3.5	0.3*
LAP012	40651	Burial 3	No collagen	-	-	-	-

[1]Curt-Engelhorn-Centre for Archaeometry, Mannheim. * Sample with very low collagen yield (0.3%) and susceptible to contamination.

Amazon - Hatahara

Analyzed Samples

Table S37: Samples from the site Hatahara analyzed for aDNA in this study.

IPHAN ID	MPI ID	Context	Bone part	DNA
MUAM-01	HTH001	Burial 27 (Child), Unit N1298W1308/9	pars petrosa	No

Archaeological context

The Hatahara site was discovered in 1997 by the Central Amazon Project, directed by Eduardo Góes Neves, James Petersen and Michael Hackenberger. The site is located on an elevated terrace near the city of Manaus, State of Amazonas, and became particularly known for: its size (approximately 20ha), with archaeological packages over 3 m deep; the identification of residential mounds; the stratigraphic complexity with at least four moments of occupation before the arrival of Europeans (going from the 4th century BCE to the 16th century CE); good preservation of organic material; presence of large amounts of Amazonian Dark Earth and ceramic fragments; and the presence of well-preserved burials. These characteristics encouraged a large number of studies to be carried out by an interdisciplinary team. To date, 28 burials and deposits with human bones have been found in the area of the mounds, totalizing at least 38 individuals. Twelve urns, with remains of human teeth were unearthed in the same site (Figure S12).

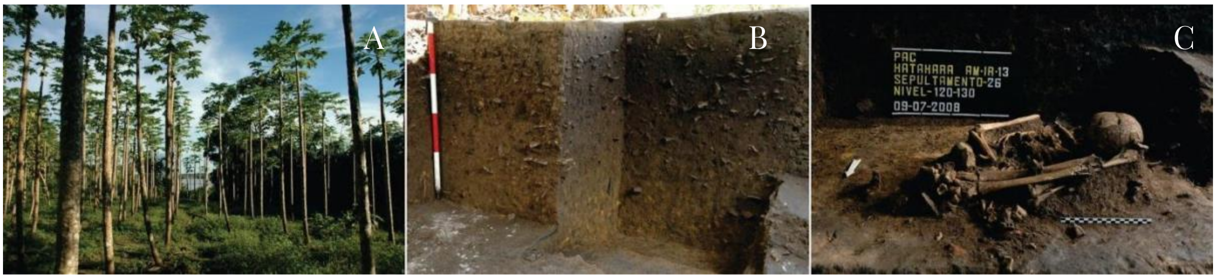

Figure S12: Hatahara. A) View of the site; B) Deep anthropogenic soil (ADE) with pottery; C) Burial 26.

Marabaixo IV

Analyzed Samples

Table S38: Samples from the site of Marabaixo IV for aDNA in this study.

IPHAN ID	MPI ID	Context	Bone part	DNA
Marabaixo-4	MRB001	Urna A sample #2	pars petrosa	No

Archaeological context

The archaeological site Marabaixo IV is located in Macapá, State of Amapá, north of Brazil. The site was identified in 2014, following the construction work conducted in a residential area of the city of Macapá. The rescue mission was carried out by members of the Núcleo de Pesquisa Arqueológica do Instituto de Pesquisas Científicas e Tecnológicas do Estado do Amapá (NuPArq/IEPA). Two urn-burials were discovered - both from shallow pits – along with a globular vessel (with no visible bones inside) and a circular pottery stool, which is consistent with other funerary contexts in the mouth of the Amazon (Figure S13). The site was registered as a burial context and the remains identified were associated with the Mazagão phase, as described by Meggers & Evans (1957) [87,88].

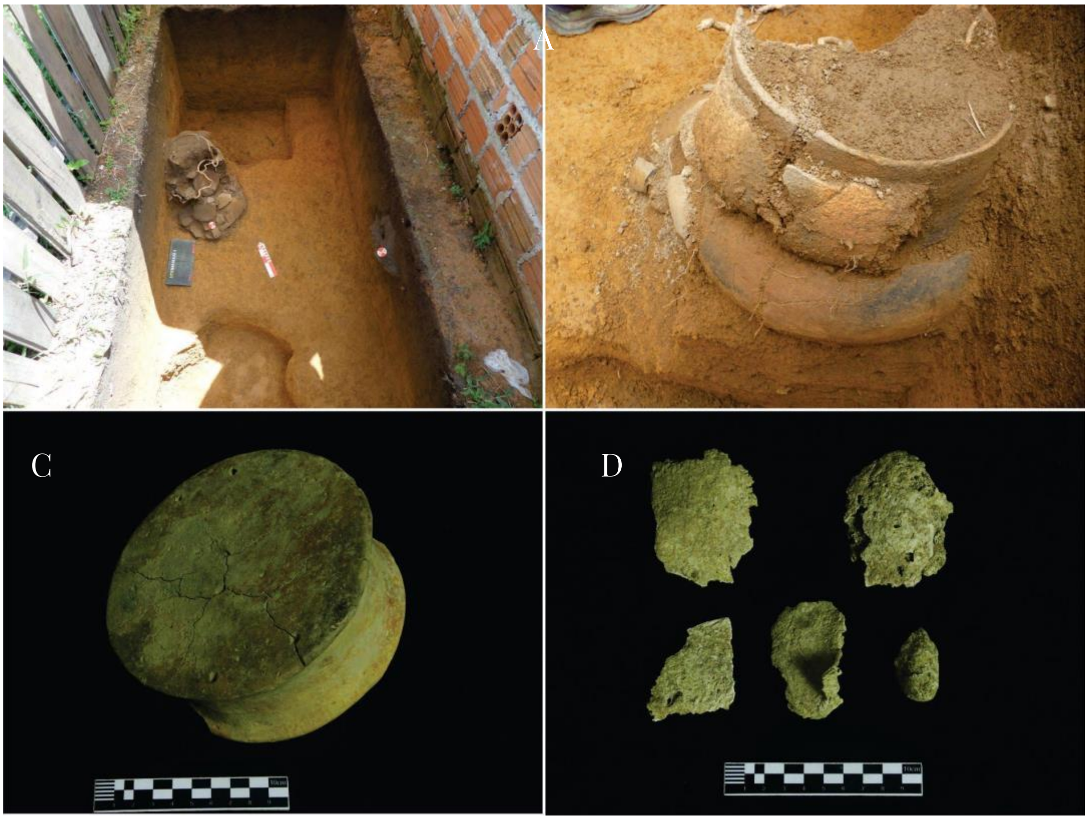

Figure S13: Marabaixo IV. A) View of the site**;** B) funerary urn recovered from the site; C - D) Artifacts associated with the urn.

Gruta das Caretas

Analyzed Samples

Table S39: Samples from the site *Gruta das Caretas* analyzed for aDNA in this study.

IPHAN ID	MPI ID	Context	Bone part	DNA
MPEG - 32	GRC001	PN 2206/291, GC 31 (box 18) (Cat.: 1150)	pars petrosa	No

Archaeological context

The Maracá society occupied the southeast of Amapá state (Amazonian estuary) during the late pre-Columbian and early colonial times. Among the most well-known features of this culture are the magnificent anthropomorphic funerary urns placed in caves and shelters, used as cemeteries. Most urns represent women and men sitting on benches, with their hands resting on their knees. These cylindrical clay sitting bodies are composed of the zoomorphic benches, and upper and lower limbs, with stylized human anatomical representations, including gender characteristics [89,90]. Originally sealed, they contain secondary burial of only one individual in each urn, of the same gender as indicated in clay [91].

The urn bodies were painted with various designs in black, red, white and yellow, along with body ornaments in modeled clay, like hoods or bows on the head, which is the lid; bracelets, bracelets, belts, and anklets. Other urns represent quadrupedal animals, similar to tortoises. The urns were not buried, dozens of them were arranged on the surface inside rockshelters. The distribution of Maracá sites in the regional topography evidences specific forms of landscaping that prioritize the areas closer to the water sources in open-air sites for habitation settlements, while the cemeteries situate on high bluffs in reserved places inside the caves [89].

Maracá sites became known to the scientific community as of the second half of the 19th century, by Ferreira Penna, the first director of the Goeldi Museum, during his first expeditions to Amapá (then the Brazilian Guiana). Since the first records of such caves [92], further research by Lima Guedes (1896) gave the initial rise to the archeology collection of the Goeldi Museum, and since then and have been safeguarded there for over 150 years.

Other prominent expeditions and studies took place in the municipality of Mazagão, highlighting the work of Vera Guapindaia [93] at the Gruta das Caretas site, from which the osteological material analyzed in this study – specifically CG-31 Urn – was recovered. Although incomplete and with some fractures, the urn preserved the human remains (Figure S14).

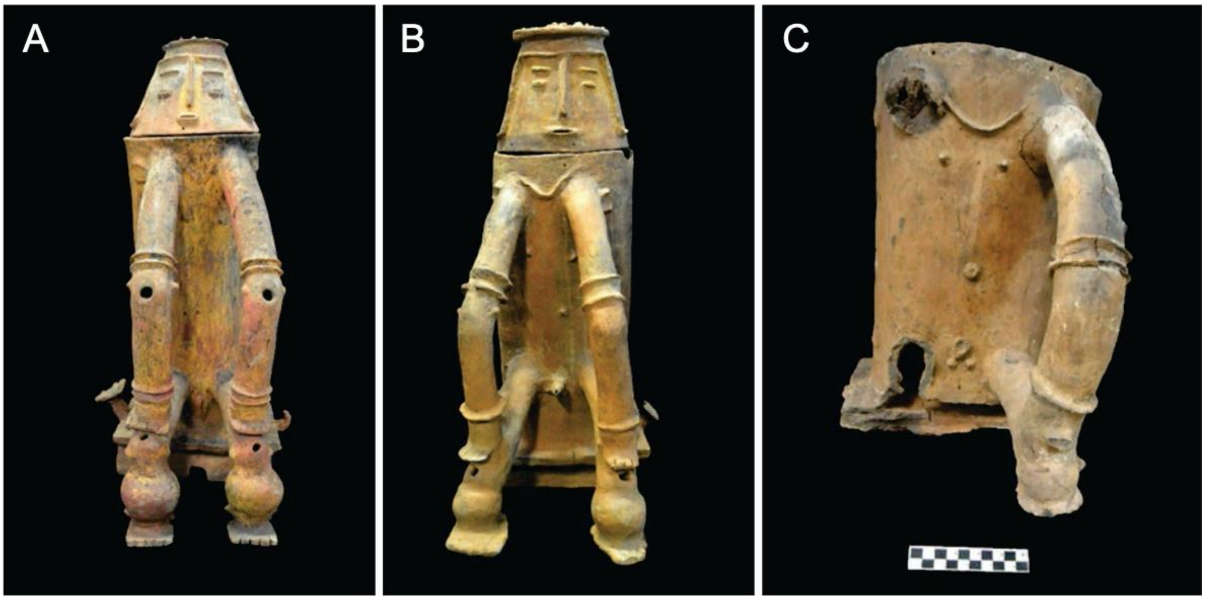

Figure S14. Gruta das Caretas. A-B) Maracá anthropomorphic funerary urns of female and male genders (GC-50 and GC-09, respectively), collected at Gruta das Caretas site in Mazagão/AP, by Guapindaia (1997). (Goeldi Museum Archeology Collection, photos by Lucas Araújo); C) CG-31 Urn, from which the osteological material analyzed in this study was collected, Gruta das Caretas site in Mazagão/AP, by Guapindaia (1997) (Goeldi Museum Archeology Collection, photo: *unknown author).

Marajoara Anthropomorphic Urn t-8 (Box: 29)

Analyzed Samples

Table S40: Samples from the site Gruta das Caretas analyzed for aDNA in this study.

IPHAN ID	MPI ID	Context	Bone part	DNA
MPEG-06	AMZ001	Urna Antropomorfa T-8 (box: 29)	pars petrosa	No

Archaeological context

Unlike the Maracá urns, in which the ceramic bodies hold the remains of a single and specific individual, the funerary urns of the Marajoara culture commonly received several individuals, or parts of them. This urn specifically, from Fazenda Teso' (Mound Farm) in the municipality of Salvaterra, eastern Marajo Island, was received by the Goeldi Museum as a donation and without detailed information about its context. Initial bioarchaeological studies of this urn, conducted by Rosa in 2018, indicated a MNI of five. Although fragmented, the urn preserved its internal content on the bottom. Its

morphology and decoration relate to the Marajoara culture, specifically the Pacoval style, where high-relief appliqués form the members of the human body, that is decorated with incisions, white and red painting (Figure S15).

The Marajoara culture thrived on the eastern part of Marajo Island for a thousand years, from ca. 400 to 1400 CE. It hasn't, though, lasted to European contact, unlike Maracá. This enormous time spam coupled with the huge internal variability of Marajoara ceramics, suggest this culture integrated several and different local communities around common livings and shared ideas, which included practices of water resources' management, mound-building, and intense social use of ceramics for ritual purposes [68,9495].

In the context of lower Amazonian archaeology, both Maracá and Marajó cultures, along with several others like Aruã, Aristé, Mazagão and Koriabo, are examples of the "mosaic of cultural diversity that proliferated in the lower Amazon before Europeans occupied the region in the seventeenth century" [96]. Bioarcheological and genetic research, like the one presented here, can be an important source to unravel populational ties that connected these mega-diversified networks of Amazonian indigenous cultures.

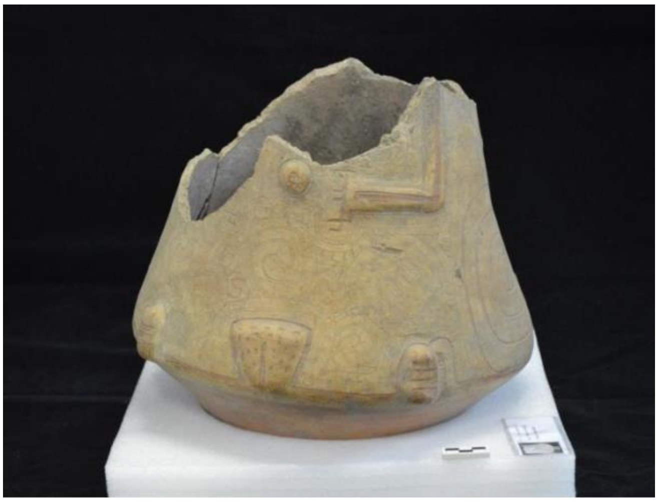

Figure S15: Marajoara Anthropomorphic urn. T-8 Marajoara anthropomorphic fragmented urn, from which the osteological sample was recovered and analyzed (Goeldi Museum Archeology Collection, source: Rosa 2018.

References

1. Carvalho, C. R. *et al.* Cabeçuda-II: um conjunto de amoladores-polidores evidenciado em Laguna, SC. *Rev. do Mus. Arqueol. e Etnol.* 401–405 (2011).
2. Farias, D. S. E. & DeBlasis, P. Programa de salvamento arqueológico e educação patrimonial na área de duplicação da BR-101 trecho ponte de Cabeçuda, Laguna/SC. *Relatório Final. Iphan (01510.000341/2007-72), Tubarão* (2014).
3. Saladino, A. A Morte Enfeitada: Um olhar sobre as práticas mortuárias dos construtores do Sambaqui Cabeçuda a partir de um sepultamento infantil. *Rev. Arqueol.* **30**, 255–257 (2017).
4. Giusto, M. N. Di. Os sambaquieiros e os outros: Estresse e estilos de vida na perspectiva da longa duração-o caso do litoral sul de Santa Catarina. (2017).
5. Kneip, A., Farias, D. & DeBlasis, P. Longa duração e territorialidade da ocupação sambaquieira na laguna de Santa Marta, Santa Catarina. *Rev. Arqueol.* **31**, 25–51 (2018).
6. Silva, R. E. da. (Re) Começando do princípio: o que a arqueografia de uma área funerária do Sambaqui de Cabeçuda pode nos ensinar sobre práticas funerárias sambaquieiras? (2020).
7. de Souza, M. & Ferraz, S. M. Estresse, doença e adptabilidade: estudo comparativo de dois grupos pré-históricos em perspectiva biocultural. (1995).
8. Rohr, J. A. Sítios Arqueológicos de Santa Catarina. in *Anais do Museu de Antropologia* 81–153 (1984).
9. Alvim, M. C. de M. E. & De Sousa, S. Relações biológicas entre populações indígenas atuais e pré-históricas do Brasil. *Clio A J. Lit. Hist. Philos. Hist.* **1**, (1990).
10. Kneip, A. O povo da lagoa: uso do SIG para modelamento e simulação na área arqueológica do Camacho. *Museu de Arqueologia e Etnologia* (2004).
11. Beck, A. A variação do conteúdo cultural dos sambaquis no litoral de Santa Catarina. 245 (1972).
12. Klokler, D. Adornos em concha do sítio Cabeçuda. *Rev. Arqueol.* **27**, 150–169 (2014).
13. Zamparetti, B. C. Sambaqui Cabeçuda 1: Um Território Resiliente. *English Language Teaching* **39**, (Universidade do Sul de Santa Catarina., 2014).
14. Mello e Alvim, M. C. de & Seyferth, G. Estudo morfológico do úmero na população do Sambaqui de Cabeçuda (Laguna, Santa Catarina). *Rev. do Mus. Paul.* **18**, 119–126 (1969).
15. Chiarini, J. A. Estudo sobre os processos formativos do Sambaqui de Cabeçuda Museu Nacional Universidade Federal do Rio de Janeiro JULIO ABREU CHIARINI Estudo sobre os processos formativos do Samb. (Universidade Federal do Rio de Janeiro, 2014).
16. Faria, L. D. C. *O problema da proteção aos sambaquis*. (1959).
17. Klokler, D. A fauna do sambaqui cabeçuda: 65 anos depois. *III Encuentro Latinoam. Zooarqueologia* (2016).
18. Alvim, M. C. de M. e & Gomes, J. C. Análise e interpretação da hiperostose porótica em crânios humanos do sambaqui de Cabeçuda (SC-Brasil). *Rev. Pré-História* **7**, 127–145 (1989).
19. Alvim, M. C. de M., Uchoa, D. P. & Gomes, J. C. de O. Cribra orbitalia e lesões cranianas congêneres em populações pré-históricas da costa meridional do Brasil. *Rev. do Mus. Arqueol. e Etnol.* **1**, 21–53 (1991).
20. Filippini, J. Treponematoses e outras paleopatologias em sítios arqueológicos pré-históricos do litoral sul e sudeste do Brasil. (2012).
21. Rodrigues, C. D. Patologias e processos dento-maxilares em remanescentes esqueletais de dois sítios pré-históricos no Brasil: o cemitério de Furna do Estrago (PE) e o Sambaqui de Cabeçuda (SC). (1997).
22. Lessa, A. & Medeiros, J. C. de. Reflexões preliminares sobre a questão da violência em populações construtoras de sambaquis: análise dos sítios Cabeçuda (SC) e Arapuan (RJ). *Rev. do Mus. Arqueol. e Etnol.* **11**, 77–93 (2001).
23. Pessanha, T. S. Uma investigação paleoepidemiológica sobre a cárie dentária em perspectiva epidemiológica e microbiológica. (2015).
24. Carvalho, C. R. & de Souza, S. M. Uso de adornos labiais pelos construtores do sambaqui de cabeçuda, Santa Catarina, Brasil: uma hipótese baseada no perfil dento-patológico. *Rev. Arqueol.* **11**, 43–55 (1998).
25. Alvim, M. C. de M., Vieira, M. I. & Cheuiche, L. M. Os construtores dos sambaquis de Cabeçudas, SC e de Piaçaguera, SP: estudo morfológico comparativo. *Arq. Anat. e Antropol.* **1**, 393–406 (1975).
26. Okumura, M. M. M. Diversidade morfológica craniana, micro-evolução e ocupação pré-histórica da costa brasileira. *Thesis* (2007).
27. DeBlasis, P., Farias, D. S. & Kneip, A. Velhas tradições e gente nova no pedaço: perspectivas longevas de arquitetura funerária na paisagem do litoral sul catarinense. *Rev. do Mus. Arqueol. e Etnol.* 109 (2014). doi:10.11606/issn.2448-1750.revmae.2014.109328
28. Villagran, X. S. O que sabemos dos grupos construtores de sambaquis? Breve revisão da arqueologia da costa sudeste do Brasil, dos primeiros sambaquis até a chegada da cerâmica Jê. *Rev. Do Mus. Arqueol. E Etnol.* **23**, 139–154 (2013).
29. Cardoso, J. M. O sítio costeiro Galheta IV: uma perspectiva zooarqueológica. (2018).
30. Assunção, D. Sambaquis da paleolaguna de Santa Marta: em busca do contexto regional no litoral sul de Santa Catarina. (2010).
31. Iriarte, J., DeBlasis, P., De Souza, J. G. & Corteletti, R. Emergent Complexity, Changing Landscapes, and Spheres of Interaction in Southeastern South America During the Middle and Late Holocene. *J. Archaeol. Res.* **25**, 251–313 (2017).
32. Cardooso, J. M., Júnior, J. A. M., Farias, D. S. E. de & Deblasis, P. Zooarqueologia do sítio Galheta IV: um enfoque nos vestígios do pinguin-de-magalhães (Spheniscus magellanucus, Spheniscidae). *Arqueofauna e Paisag.* 155–169 (2014).
33. Colonese, A. C. *et al.* Long-term resilience of late Holocene coastal subsistence system in southeastern South America. *PLoS One* (2014). doi:10.1371/journal.pone.0093854

34. Figuti, L. Construindo o sambaqui: a ocupação e os processos de construção de sítio na bacia do Canal do Palmital, Santa Catarina. Relatório final FAPESP, processo 08/01285-0 (2009).
35. Bandeira, D. da R., Oliveira, E. L. de & Santos, A. M. P. dos. Estudo estratigráfico do perfil nordeste do Sambaqui Cubatão I, Joinville/SC. *Rev. do Mus. Arqueol. e Etnol.* 119–142 (2009). doi:10.11606/issn.2448-1750.revmae.2009.89880
36. Fossile, T. et al. Pre-Columbian fisheries catch reconstruction for a subtropical estuary in South America. Fish Fish. 20, 1124–1137 (2019).
37. Pezo-Lanfranco, L., DeBlasis, P. & Eggers, S. Weaning process and subadult diets in a monumental Brazilian shellmound. *J. Archaeol. Sci. Reports* **22**, 452–469 (2018).
38. Peixe, S. P., de Melo Junior, J. C. F. & da Rocha Bandeira, D. Paleoetnobotânica dos macrorestos vegetais do tipo trançados de fibras encontrados no sambaqui Cubatão I, Joinville–SC. *Rev. do Mus. Arqueol. e Etnol.* 211–222 (2007).
39. Sá, J. C. Arqueologia Experimental: Desatando Informações Sobre Nós E Amarrações No Sambaqui Cubatão I. (2015).
40. Costa, R. L. & Lima, T. A. Artefatos trançados na Pré-história do Sul do Brasil: persistências e rupturas tecnológicas em tempos históricos. *Rev. do Mus. Arqueol. e Etnol.* 55–83 (2018).
41. Melo Júnior, J. C. F. de, Silveira, E. R. da & Bandeira, D. da R. Arqueobotânica de um sambaqui sul-brasileiro: integrando indícios sobre o paleoambiente e o uso de recursos florestais. *Bol. do Mus. Para. Emílio Goeldi. Ciências Humanas* **11**, 727–744 (2016).
42. DeBlasis, P., Kneip, A., Scheel-Ybert, R., Giannini, P. C. & Gaspar, M. D. Sambaquis e Paisagem - Dinâmica natural e arqueologia regional no litoral do sul do Brasil. *Arqueol. Suramericana* **3**, 29–61 (2007).
43. Klokler, D. Food for body and soul: mortuary ritual in shell mounds (Laguna-Brazil). 2008. (2008).
44. Simoes, C. B. O processo de formação dos sambaquis: uma leitura estratigráfica do sítio Jabuticabeira II, SC. (2007).
45. Hubbe, M., Okumura, M., Bernardo, D. V & Neves, W. A. Cranial morphological diversity of early, middle, and late Holocene Brazilian groups: Implications for human dispersion in Brazil. *Am. J. Phys. Anthropol.* **155**, 546–558 (2014).
46. Okumura, M. M. M. & Eggers, S. The people of Jabuticabeira II: reconstruction of the way of life in a Brazilian shellmound. *Homo* **55**, 263–281 (2005).
47. Fish, P. R. *et al.* Monumental shell mounds as persistent places in southern coastal Brazil. *Archaeol. Hist. Ecol. small scale Econ.* 120–140 (2013).
48. Boyadjian, C. H. C., Eggers, S. & Scheel-Ybert, R. Evidence of plant foods obtained from the dental calculus of individuals from a Brazilian shell mound. *Wild Harvest Plants hominine pre-agrarian Hum. world. Stud. Sci. Archaeol. Oxford Oxbow Books* 215–240 (2016).
49. Pezo-Lanfranco, L. *et al.* Middle Holocene plant cultivation on the Atlantic Forest coast of Brazil? *R. Soc. open Sci.* **5**, 180432 (2018).
50. Figuti, L., Plens, C. R. & DeBlasis, P. Small sambaquis and big chronologies: shellmound building and hunter-gatherers in Neotropical highlands. *Radiocarbon* **55**, 1215–1221 (2013).
51. Plens, C. R. Arqueologia Funerária: a materialidade da vida após a morte. *Rev. M. Estud. sobre a morte, os mortos e o morrer* **3**, 318–343 (2018).
52. Mafra, F. (2020). Lugares persistentes, práticas funerárias e tecnologia ceramista em caçadores-coletores pré-históricos sul-americanos: Uma proposta de interpretação para o sítio arqueológico Pedra do Alexandre, Carnaúba dos Dantas, RN, Brasil. Clio vol 35 (3): 60-116
53. PLENS, C. R. The Hunter-Gatherers' Riverine Mound Builders from the Brazilian Atlantic Forest in the Southeast. (2018).
54. Plens, C. R. Sıtio Moraes, uma biografia nao autorizada: análise do processo de formaçao de um sambaqui fluvial [PhD thesis]. *Univ. Sao Paulo Univ. Sao Paulo* (2008).
55. Koole, E. K. M. Pré-história da província Cárstica do Alto São Francisco, Minas Gerais: A indústria lítica dos caçadores-coletores arcaicos. (2007).
56. Strauss, A. *et al.* Two directly dated early Holocene Archaic burials from Pains, state of Minas Gerais, Brazil. *Curr. Res. Pleistocene* **28**, 123–125 (2011).
57. Mafra, F. lugares persistentes, práticas funerárias e tecnologia ceramista em caçadores-coletores pré-históricos sul-Americanos: uma proposta de interpretação para o sítio arqueológico Pedra do Alexandre, Carnaúba dos Dantas, RN, Brasil,. 60–116 (2020). doi:10.20891/clio.
58. Da Silva, S. F. S. M. & Solari, A. O Sítio arqueológico Pedra do Alexandre, Seridó, Rio Grande do Sul: Principais resultados de estudos, os sepultamentos e perspectivas para o futuro. *Clio Arqueol.* 117–169 (2020). doi:10.20891/clio.
59. Castro, A. M., Müller, L. M., Heinen, I. L. S. & Kipinis, R. The Koriabo pottery in the Volta Grande do Rio Xingu. in *Koriabo: from the Caribbean Sea to the Amazon River* 203–226 (2021).
60. Rostain, S. Koriabo Pottery In The Guianas. in *Koriabo: from Caribbean Sea to the Amazon River* 55 (2021).
61. de Souza Barreto, B. Understanding Jari and Koriabo Ceramics from Southern Amapá. in *Koriabo: from Caribbean Sea to the Amazon River* 121 (2021).
62. Van den Bel, M. A Koriabo site on the Lower Maroni River: results of the preventive archaeological excavation at Crique Sparouine, French Guiana. *Arqueol. Amaz.* **1**, 61–93 (2010).
63. van den Bel, M., Hildebrand, M. & Mestre, M. Koriabo in French Guiana: Cultural Expansion in the Guianas During Late Prehistoric Times. in *Koriabo: from Caribbean Sea to the Amazon River* 81 (2021).
64. Hofman, C., Garcia, L., Hildebrand, M. & Hofman, C. L. Halfway between the Guianas and Lower Amazon: Archaeology in Trombetas basis. in *Koriabo: from the Caribbean Sea to the Amazon Rive* (2021).
65. de Moura Saldanha, J. D. & Cabral, M. P. On Change and Exchange: a Review of Koriabo Contexts and Concept in

the Eastern Guianas. *Koriabo from Caribb. Sea to Amaz. River* 97 (2021).
66. Boomert, A. The Cayo complex of St. Vincent: Ethnohistorical and archaeological aspects of the Island Carib problem. *Antropológica* **66**, 3–68 (1986).
67. Lima, H. P. *et al.* Koriabo Ceramics, Carib Multiethnic Interaction Spheres and the Colonial Enterprise in the Lower Amazon. in *Koriabo: from Caribbean Sea to the Amazon River* 245 (2021).
68. Barreto, C. O que a cerâmica Marajoara nos ensina sobre fluxo estilístico na Amazônia? in 115–124 (2016).
69. Hofman, C. L., Hoogland, M. L. P., Jacobson, K., Manem, S. & Boomert, A. Cayo in the Lesser Antilles: a Network of Peoples, Places and Practices in the Late 15th to Early 17th Century. in *Koriabo: from Caribbean Sea to the Amazon River* 33 (2021).
70. Garcia, L. Tupi-Carib Histories in the Middle-Lower Xingu. in *Koriabo: from the Caribbean Sea to the Amazon Rive* 227 (2021).
71. Hattori, M. L. & Strauss, A. Kiju Sakai: o antropólogo japonês que dedicou sua vida a estudar o Brasil na primeira metade do século XX. *Bol. do Mus. Para. Emílio Goeldi. Ciências Humanas* **11**, 715–726 (2016).
72. Barbosa, A. S. *Andarilhos da claridade: os primeiros habitantes do cerrado*. (Universidade Católica de Goiás, Instituto do Trópico Subúmido, 2002).
73. Janine Carvalho, R. Novas perspectivas a partir das coleções osteológicas do sítio Vau 1 no município de Sta. Maria Vitória -BA. *Ind. High. Educ.* **3**, 1689–1699 (2021).
74. Shock, M. P., Kipnis, R., Bueno, L. & Silva, F. M. A chronology of the introduction of domesticated plants in Central Brazil. *Tipití J. Soc. Anthropol. Lowl. South Am.* **11**, 52–59 (2013).
75. Schmitz, P. I., Sales Barbosa, A., Jacobus, A. L. & Barberi Ribeiro, M. Arqueologia nos cerrados do Brasil Central: Serranópolis I. *Pesqui. Antropol.* (1989).
76. Wüst, I. Etnicidade e tradições ceramistas: algumas reflexões a partir das antigas aldeias Bororo do Mato Grosso. *Rev. do Mus. Arqueol. e Etnol. Supl.* 303–317 (1999).
77. Wüst, I. *Aspectos da ocupação pré-colonial em uma área do Mato Grosso de Goiás: tentativa de análise espacial*. (UNESC, 1983).
78. Schmitz, P. I., Wust, I., MOEHLECKE COPE, S. & ELFRIEDE THIES, U. M. Arqueologia do Centro-Sul de Goias. Uma fronteira de horticultores indigenas no Centro do Brasil. *Pesqui. Antropol.* 1–281 (1982).
79. de Souza, J. G., Mateos, J. A. & Madella, M. *Archaeological expansions in tropical South America during the late Holocene: Assessing the role of demic diffusion*. *PLoS ONE* **15**, (2020).
80. de Carvalho, O. A. & Oliveira, C. Sítio Jerimum, Xingó, Brasil: Primeira abordagem paleoantropológica. *Canindé* **2**, 103 (2002).
81. CARVALHO, O. A. de & VERGNE, C. Estudo paleodemográfico e tafonômico na população pré-histórica da necrópole de São José II (Delmiro Gouveia, Alagoas, Brasil). *Rev. Canindé* **1**, 101–116 (2001).
82. Fagundes, M. Entendendo a dinâmica cultural em Xingó na perspectiva inter-sítios: indústrias líticas e os lugares persistentes no baixo vale do rio São Francisco, nordeste do Brasil. *Arqueol. Iberoam.* **6**, 3–23 (2010).
83. Silva, J. A. Ambientes funerários e a contribuição para novas leituras arqueológicas: adornos em sepulturas humanas do sítio Justino/SE, como evidência do contato Nativo Americano/Europeu. (2017).
84. Vergne, M. C. de S. & Morais, J. L. de P. P.-S. P. Arqueologia do Baixo São Francisco: estruturas funerárias do Sítio Justino - região de Xingó, Canindé do São Francisco, Sergipe. (2004).
85. Oliveira, L. & Klokler, D. Corpos, oferendas, rituais e gênero no Sítio Justino, Baixo São Francisco. *Habitus Rev. do Inst. Goiano Pré-História e Antropol.* **16**, 103–124 (2018).
86. Oliveira, C. A. & Cisneiros, D. Grupos pré-históricos do Sítio Jerimum região de Xingó–Canindé do São Francisco, SE. *Aracaju MAX* (2005).
87. Saldanha, J. & Cabral, M. Estruturas rituais pré-coloniais na costa do Amapá. *Rev. Habitus-Revista do Inst. Goiano Pré-História e Antropol.* **14**, 73–86 (2016).
88. Meggers, B. & Evans, C. Archaeological Investigations at the Mouth of the Amazon. Washington. *Smithson. Institution, Bur. Am. Ethnol.* (1957).
89. Augusto Palheta Barbosa, C. As iconografias das urnas funerárias antropomorfas Maracá (Amapá): a coleção Gruta das Caretas. *Rev. Arqueol.* **24**, 140–143 (2011).
90. GUAPINDAIA, V. L. C. Encountering the Ancestors: the Maracá funerary urns. *Unkn. Amaz. Nat. Cult. Anc. Brazil* 156–174 (2001).
91. Souza, S. M. de, Guapindaia, V. L. C. & Carvalho, C. R. A necrópole Maracá e os problemas interpretativos em um cemitério sem enterramentos. (2001).
92. Ferreira Penna, D. S. Apontamentos sobre os cerâmios do Pará. *Arch. do Mus. Nac. do Rio Janeiro* **2**, 47–67 (1877).
93. Guapindaia, V. L. C. & Machado, A. L. da C. O potencial arqueológico da região do Maraca/Igarapé do Lago (AP). (1997).
94. Schaan, D. P. The nonagricultural chiefdoms of Marajó Island. in *The handbook of South American archaeology* 339–357 (Springer, 2008).
95. Roosevelt, A. Late Amazonian. in *Encyclopedia of Prehistory* 195–199 (Springer, 2001).
96. Guapindaia, V. Prehistoric funeral practices in the Brazilian Amazon: the Maraca urns. in *The handbook of South American archaeology* 1005–1026 (Springer, 2008).
