# Supplementary material for: Genomic history of coastal societies from eastern South America
Source: Nat Ecol Evol. 2023 Jul 31;7(8):1315–30. doi: 10.1038/s41559-023-02114-9 (PMC10406606; doi:10.1038/s41559-023-02114-9)
Supplement: Supplementary file 2 — Reporting Summary [file 41559_2023_2114_MOESM2_ESM.pdf]

nature research

Corresponding author(s): Tábita Hünemeier, André Strauss and Cosimo Posth

Last updated by author(s): 04/21/2023

Reporting Summary

Nature Research wishes to improve the reproducibility of the work that we publish. This form provides structure for consistency and transparency in reporting. For further information on Nature Research policies, see our Editorial Policies and the Editorial Policy Checklist.

Statistics

For all statistical analyses, confirm that the following items are present in the figure legend, table legend, main text, or Methods section.

n/a	Confirmed	
☐	☒	The exact sample size (*n*) for each experimental group/condition, given as a discrete number and unit of measurement
☐	☒	A statement on whether measurements were taken from distinct samples or whether the same sample was measured repeatedly
☐	☒	The statistical test(s) used AND whether they are one- or two-sided<br>*Only common tests should be described solely by name; describe more complex techniques in the Methods section.*
☐	☒	A description of all covariates tested
☐	☒	A description of any assumptions or corrections, such as tests of normality and adjustment for multiple comparisons
☐	☒	A full description of the statistical parameters including central tendency (e.g. means) or other basic estimates (e.g. regression coefficient) AND variation (e.g. standard deviation) or associated estimates of uncertainty (e.g. confidence intervals)
☐	☒	For null hypothesis testing, the test statistic (e.g. *F*, *t*, *r*) with confidence intervals, effect sizes, degrees of freedom and *P* value noted<br>*Give P values as exact values whenever suitable.*
☒	☐	For Bayesian analysis, information on the choice of priors and Markov chain Monte Carlo settings
☒	☐	For hierarchical and complex designs, identification of the appropriate level for tests and full reporting of outcomes
☒	☐	Estimates of effect sizes (e.g. Cohen's *d*, Pearson's *r*), indicating how they were calculated

Our web collection on statistics for biologists contains articles on many of the points above.

Software and code

Policy information about availability of computer code

Data collection	EAGER v.1.92.56<br>fastqc v.0.11.4<br>BWA v.0.7.12<br>circularmapper v.1.93.5<br>AdapterRemoval v.2.3.1<br>dedup v.0.12.2<br>mapDamage v.2.0.9<br>samtools v.1.3<br>pileupCaller v.1.4.0.2<br>PMDtools v.0.6<br>EIGENSOFT v.7.2.1 (convertf)<br>PLINK v.1.9

Data analysis

schmutzi
Haplofind
Haplogrep
mafft v.7.305
MEGA v.10.1.5
EIGENSOFT v.7.2.1 (smartpca)
AdmixTools 5.1 (qp3Pop, qpDstat, qpWave, qpF4ratio)
RStudio v.1.2.1335
RColorBrewer v.1.1.2

For manuscripts utilizing custom algorithms or software that are central to the research but not yet described in published literature, software must be made available to editors and reviewers. We strongly encourage code deposition in a community repository (e.g. GitHub). See the Nature Research guidelines for submitting code & software for further information.

Data

Policy information about availability of data

All manuscripts must include a data availability statement. This statement should provide the following information, where applicable:

- Accession codes, unique identifiers, or web links for publicly available datasets
- A list of figures that have associated raw data
- A description of any restrictions on data availability

Alignment files of the nuclear and mitochondrial DNA sequences for the newly reported individuals will be available upon publication at the European Nucleotide Archive (ENA) under the accession number PRJEB51863.

Field-specific reporting

Please select the one below that is the best fit for your research. If you are not sure, read the appropriate sections before making your selection.

☒ Life sciences ☐ Behavioural & social sciences ☐ Ecological, evolutionary & environmental sciences

For a reference copy of the document with all sections, see nature.com/documents/nr-reporting-summary-flat.pdf

Life sciences study design

All studies must disclose on these points even when the disclosure is negative.

Sample size

We performed all population genomics analyses on 34 human individuals, starting from the extraction of ancient DNA from the dense part of the pars petrosa in the temporal bone, teeth or long bones. The skeletal remains studied covered different Brazilian regions, archaeological contexts and time periods.

Data exclusions

We excluded 61 human individuals from the population genomics analysis. Those samples did not fulfill our quality control criteria (human DNA proportion, ancient DNA damage and modern-day DNA contamination levels).

Replication

Replication is achieved by performing analyses on genome-wide single nucleotide polymorphisms (SNPs) and merging the newly produced ancient DNA data with different SNP panels (1240K, Human Origins, Illumina datasets).

Randomization

We applied different quality controls including the authentication of ancient DNA (human DNA proportion, ancient DNA damage and contamination estimate) and clustering methods (MDS, PCA and Unsupervised Admixture). After the application of those quality controls, we grouped individuals based on date, archaeological context, and their genetic affinities based on f3- and f4-statistics.

Blinding

No blinding was performed. The genotypes of the single- and double-stranded libraries were combined after confirmation of similar statistical behaviour.

Reporting for specific materials, systems and methods

We require information from authors about some types of materials, experimental systems and methods used in many studies. Here, indicate whether each material, system or method listed is relevant to your study. If you are not sure if a list item applies to your research, read the appropriate section before selecting a response.

Materials & experimental systems

n/a	Involved in the study	
☒	☐	Antibodies
☒	☐	Eukaryotic cell lines
☐	☒	Palaeontology and archaeology
☒	☐	Animals and other organisms
☒	☐	Human research participants
☒	☐	Clinical data
☒	☐	Dual use research of concern

Methods

n/a	Involved in the study	
☒	☐	ChIP-seq
☒	☐	Flow cytometry
☒	☐	MRI-based neuroimaging

Palaeontology and Archaeology

Specimen provenance	Permits for exporting the material for aDNA analysis were obtained from the Instituto do Patrimônio Histórico e Artístico Nacional (IPHAN) and sampling access was granted by the local curators at the following housing institutions: Museu de Arqueologia e Etnologia da Universidade de São Paulo (MAE-USP), Instituto de Biociências da Universidade de São Paulo (IB-USP), Superintendência no Espírito Santo do Instituto do Patrimônio Histórico e Artístico Nacional (IPHAN-ES), Universidade Federal do Amapá (UNIFAP), Museu Amazônico da Universidade Federal do Amazonas (UFAM), Museu Paraense Emílio Goeldi (MPEG), Scientia Consultoria Científica, Museu de Arqueologia do Xingó da Universidade Federal de Sergipe (MAX-UFS), Museu Arqueológico do Carste do Alto São Francisco (MAC), Grupo de Pesquisa em Educação Patrimonial e Arqueologia (GRUPEP), Instituto Goiano de Pré-História e Antropologia da Pontifícia Universidade Católica de Goiás (IGPA-PUCGO), Museu Histórico de Lins (MHL), Universidade Federal de Pernambuco (UFPE).
Specimen deposition	A portion of the human skeletal elements analyzed in this study as well as DNA extracts and genetic libraries are stored at Department of Archaeogenetics of the Max Planck Institute for Evolutionary Anthropology, Germany.
Dating methods	We directly radiocarbon-dated 25 individuals to estimate the time-frame of the newly reported ancient genomes. One bone fragment from each individual was sent to Curt-Engelhorn-Zentrum Archäometrie GmbH in Mannheim and the resulting dates are presented in Data S1 and S8. Pretreatment processes, quality control protocols, and dating methods performed by the Mannheim lab are provided in the Methods section.

☒ Tick this box to confirm that the raw and calibrated dates are available in the paper or in Supplementary Information.

Ethics oversight	No ethical approval or guidance was required as the research did not involve present-day human samples.

Note that full information on the approval of the study protocol must also be provided in the manuscript.
